# A CIA strategy with eminent drug-loading capacities for tumor ferroptosis-gas synergistic therapy

**DOI:** 10.7150/thno.99295

**Published:** 2024-10-28

**Authors:** Jiaoyang Zhu, Lin Huang, Jing Yang, Zongheng Li, Lihe Wu, Wei Xiong, Jie Feng, Chenggong Yan, Chaomin Chen, Yan Li, Zheyu Shen

**Affiliations:** 1 Biomaterials Research Center, School of Biomedical Engineering, Southern Medical University, 1023 Shatai South Road, Baiyun, Guangzhou, Guangdong 510515, China.; 2 Medical Imaging Center, Nanfang Hospital, Southern Medical University, 1023 Shatai South Road, Baiyun, Guangzhou, Guangdong 510515, China.; 3 Institute of Medical Instruments, School of Biomedical Engineering, Southern Medical University, 1023 Shatai South Road, Baiyun, Guangzhou, Guangdong 510515, China.

**Keywords:** controlled ideal aggregation (CIA), magnetic resonance imaging (MRI), contrast agent (CA), tumor ferroptosis-gas synergistic therapy, tumor microenvironment (TME) responsiveness

## Abstract

**Rationale**: A common challenge of drug loading and delivery using magnetic resonance imaging (MRI) contrast agents (CAs) is the tendency of aggregation and precipitation at high drug loading conditions. Herein, we propose a generic strategy of controlled ideal aggregation (CIA) to restrict the tendency.

**Methods**: Fe^2+^, β-Lapachone (LAP), brequinar (BQR), or Sorafenib (SOR) was respectively loaded onto Gd poly (acrylic acid) macrochelate (GP), an MRI CA, in the hollow core of nitrite-modified hollow mesoporous organosilica nanoparticles (HMON-SNO). The aggregation of FeGP, LAPGP, BQRGP, and SORGP was controlled to be ideal without precipitation by the fixed space of the HMON-SNO hollow core. The sizes of the ideal aggregates are larger than the mesopore size of HMON-SNO, which prevents premature drug leakage and release.

**Results**: After the accumulation of FeGP@HMON-SNO in tumors, the presence of glutathione (GSH) in the tumor microenvironment (TME) triggers the HMON-SNO degradation to release NO, Fe^2+,^ and GP. The released Fe^2+^ reacts with endogenous hydrogen peroxide (H_2_O_2_) to generate Fe^3+^ and hydroxyl radical (**•**OH). The -SNO groups on the surface of HMON-SNO react with GSH, enabling sustained NO generation. The elevated NO level induces mitochondrial dysfunction, down-regulates lipid droplets through the alleviation of hypoxia and consequently promotes the accumulation of lipid peroxidation (LPO) under excess **•**OH to induce tumor cell ferroptosis. Moreover, the released GP facilitates high contrast *T*_1_-weighted MRI of tumors due to its high *r*_1_ value, enabling real-time monitoring for the *in vivo* delivery of FeGP@HMON-SNO.

**Conclusions**: The proposed strategy of CIA with universality was successfully utilized to restrict the aggregation of MRI CAs at high drug loading conditions. The developed FeGP@HMON-SNO with eminent drug loading content were used for tumor ferroptosis-gas synergistic therapy with high efficacy.

## 1. Introduction

Magnetic resonance imaging (MRI) has emerged as a commonly used technique for tumor diagnosis due to its non-invasiveness, excellent tissue penetration, and high spatial resolution^1^-^5^. Contrast agents (CAs) of MRI play a crucial role in enhancing the contrast between normal and tumor tissues. Among the MRI CAs, *T*_1_-weighted CAs are particularly valuable for clinical applications as they significantly enhance the brightness of tumor regions^6^-^9^. Gd-based CAs (GBCAs), a kind of important *T*_1_-weighted CAs, include small molecular Gd chelates (widely used in clinics), Gd macrochelates, and Gd-based nanoparticles^10^, ^11^. Gd-based nanoparticles and Gd macrochelates exhibit higher longitudinal relaxivities (*r*_1_) compared with small molecular Gd chelates. Previously, we reported the MRI performance of ultra-small Gd oxide nanoparticles (GO) and Gd poly (acrylic acid) macrochelate (GP) with *r*_1_ values of 70.2 ± 1.8 mM^-1^ s^-1^ and 56.23 ± 1.69 mM^-1^ s^-1^^12^, which are much higher than that of the commercial small molecular Gd chelates (3-11 mM^-1^ s^-1^), making them promising for clinical applications (*e.g.*, tumor diagnosis).

Because of the tumor targetability including passive targeting and active targeting, nanomedicines bring hope for tumor treatment^13^-^15^. Pharmacokinetics and *in vivo* distribution play a crucial role in the therapeutic efficacy of nanomedicine. Normal tissues and tumors of animals are usually resected for analysis of *in vivo* distribution^16^, ^17^. However, it is hard to trace the *in vivo* distribution of nanomedicines for human beings. One of the best solutions is loading drugs on MRI CAs to make the nanomedicines, whose *in vivo* distribution could be visualized by MRI^18^-^20^. Nevertheless, a common challenge of drug loading and delivery using MRI CAs is the tendency of aggregation and precipitation at high drug loading conditions.

Ferroptosis is an emerging form of regulated cell death characterized by iron-dependent lipid peroxidation, resulting in cellular membrane damage^21^. Research indicates that tumor cells with a high mesenchymal phenotype often exhibit resistance to conventional cancer therapies but remain susceptible to ferroptosis. This sensitivity has generated considerable interest in ferroptosis as a pivotal strategy within nanomedicine for cancer treatment. Distinct from traditional cell death pathways such as apoptosis, necrosis, and autophagy, ferroptosis is primarily driven by elevated intracellular iron levels and the suppression of glutathione (GSH) synthesis, leading to oxidative damage^22^. Key mechanisms underlying ferroptosis include iron accumulation, free radical generation, and lipid peroxidation. Iron-based nanomaterials can release free iron ions upon degradation in the acidic tumor microenvironment (TME), thereby activating ferroptosis through the Fe^2+^-mediated Fenton reaction^23^. Furthermore, the released and generated Fe^3+^ ions can deplete intracellular GSH *via* redox reactions, resulting in the downregulation of GPX4 and compromising the tumor cells' antioxidant defenses^24^. Consequently, iron-based nanomaterials hold significant promise for enhancing tumor-specific ferroptosis.

Gas therapy has recently gained attention for its potential to enhance ferroptosis in cancer treatment, utilizing various gaseous agents such as H_2_, CO, H_2_S, SO_2_, and NO 25, 26. Among these, NO stands out for its role as an apoptosis inducer in cancer therapy. As a highly diffusible free radical, NO can interact with reactive oxygen species (ROS) to form peroxynitrite (ONOO⁻), a more potent reactive nitrogen species (RNS) that induces apoptosis more effectively than most ROS by peroxidizing and nitrifying biomolecules. Remarkably, the integration of tumor ferroptosis with NO-based therapies represents a novel frontier in cancer treatment, leveraging the unique mechanisms of ferroptosis alongside the therapeutic release of gaseous signaling molecules. For example, a research group led by Prof. Ji has developed an acid-activated prodrug designed to initiate an intracellular cascade that enhances ferroptosis therapy for melanoma 27, underscoring the potential of combining ferroptosis with NO therapies to improve therapeutic outcomes in oncology. However, a major concern regarding small NO donors is their specificity and safety, as they often exhibit poor stability, a short half-life, and uncontrollable release. These characteristics can result in off-target effects, potentially causing damage to normal tissues.

**Scheme 1:**
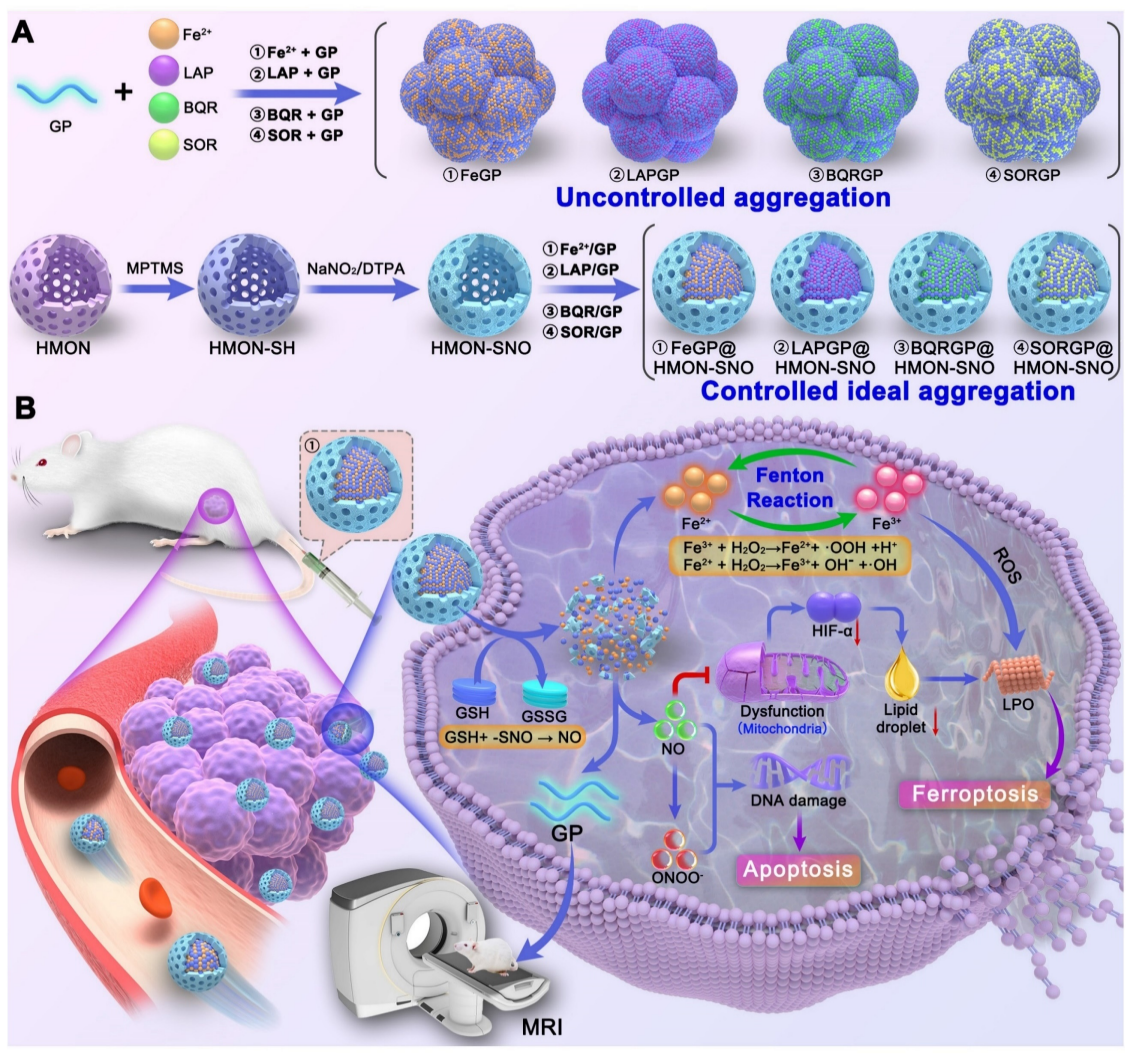
(A) Schematic illustration of the synthesis of uncontrollable FeGP, LAPGP, BQRGP, and SQRGP aggregates, along with the preparation of FeGP@HMON-SNO, LAPGP@HMON-SNO, BQRGP@HMON-SNO, and SQRGP@HMON-SNO using our proposed CIA strategy. (B) Schematic illustration for the MRI-guided ferroptosis-gas synergistic therapy of triple negative breast cancer using FeGP@HMON-SNO.

Herein, this study proposes a controlled ideal aggregation (CIA) strategy with eminent drug-loading capacities for tumor ferroptosis-gas synergistic therapy, potentially overcoming the aforementioned issues. Typically, hollow mesoporous organosilica nanoparticles (HMON) were synthesized, and modified with thiol groups (-SH) on the surface, which were then converted to -SNO groups using sodium nitrite, yielding HMON-SNO. After that, Fe^2+^, β-Lapachone (LAP), brequinar (BQR), or Sorafenib (SOR) was loaded on the above-mentioned GP in the hollow core of HMON-SNO. The aggregation of FeGP, LAPGP, BQRGP, and SORGP with very high drug loading contents (DLC) was controlled to be ideal without precipitation by the fixed space of the HMON-SNO hollow core. The sizes of the ideal aggregates are larger than the mesopore size of HMON-SNO, which prevents premature drug leakage. Based on the validation of these drugs, the CIA strategy is expected to be used for generic drug loading of MRI CAs at high drug loading conditions (Scheme 1A). Subsequently, we selected the classic Fenton reaction agent Fe^2+^ for further experiments.

After the accumulation of FeGP@HMON-SNO in tumors (Scheme 1B), the presence of GSH in the TME triggers the HMON-SNO degradation to release nitric oxide (NO), Fe^2+^, and GP. The released Fe^2+^ reacts with endogenous H_2_O_2_ to generate Fe^3+^ and hydroxyl radical (•OH). The -SNO groups on the surface of HMON-SNO react with GSH, enabling sustained NO generation^28^. Elevated levels of NO induce mitochondrial dysfunction. Mitochondria serve not only as the primary site for cellular energy production but also as crucial organelles for inducing hypoxia inducible factor-1 (HIF-1α) expression under hypoxic conditions. Impaired mitochondrial function can increase intracellular oxygen tension, leading to accelerated degradation of HIF-1α and subsequent reduction in its levels. This sequence alleviates hypoxia, decreases lipid droplet formation, and fosters the accumulation of lipid peroxides (LPO), thereby further promoting tumor cell ferroptosis. Moreover, the released GP facilitates high contrast *T*_1_-weighted MRI of tumors due to its high *r*_1_ value, enabling real-time monitoring for the *in vivo* delivery of FeGP@HMON-SNO. In summary, our study presents a multifaceted approach to cancer therapy that combines the precision of MRI-guided drug delivery with the potency of ferroptosis and NO gas therapy, offering a promising avenue for cancer treatment with enhanced efficacy and safety profiles.

## 2. Material and methods

### 2.1. Synthesis of HMON-SH

10.0 mL of HMON dispersion in anhydrous ethanol was pre-mixed with 300 µL of ammonia solution (28%) and 400 µL of 3-mercaptopropyltrimethoxysilane, and then stirred overnight at room temperature. The resulting HMON-SH products were obtained after centrifugation (20000 × g, 20 min), and subsequently washed three times with anhydrous ethanol solution.

### 2.2. Synthesis of HMON-SNO

To perform S-nitrosoglutathione modification, 7.0 mL of HMON-SH dispersion (10 mg/mL) was mixed with 8.0 mL of methanol. Subsequently, 4.0 mL of HCl (5.0 M) was added to the mixture, which was then kept for 1.0 h under magnetic stirring in an ice bath. After that, 320 mg of NaNO_2_ was dissolved in 4.0 mL of Diethylenetriaminepentaacetic acid (DTPA, 500 uM), which was added to the above reaction system. The obtained mixture was then kept for 1.0 h under magnetic stirring in an ice bath and darkness. The HMON-SNO product was collected *via* centrifuge (20000 × g, 20 min), washed twice with 10 mL of methanol, finally dispersed in 2.0 mL of methanol, and stored in the fridge for next use.

### 2.3. Synthesis of FeGP, LAPGP, BQRGP, SORGP

0.1 mL of GP (*C*_Gd_ = 13.1 mM, *C*_GP_ = 10.8 mg/mL) aqueous solutions was mixed with 0.02-0.32 mL of FeCl_2_ solution (4.0 mg/mL), resulting in mass ratios of Fe/GP at 0.074, 0.148, 0.296, 0.593, or 1.186. Additionally, 0.02-0.16 mL of LAP, BQR, and SOR solutions (4.0 mg/mL) were mixed with the GP solution, leading to mass ratios of ferroptosis agonist/GP at 0.074, 0.148, 0.296, or 0.593, respectively. The total volume of the mixtures was standardized to 1.0 mL, and the mixtures were further stirred for 24 h. The resulting samples were centrifuged and washed at 20000 × g for 15 min at 4.0 °C to obtain FeGP1-5, LAPGP1-4, BQRGP1-4, and SORGP1-4. The Fe and Gd loading content in the FeGP@HMON-SNO was determined using ICP-OES (iCAP PRO, Thermo Fisher Scientific, US), while the LAP and BQR loading content was analyzed using UV-Vis spectroscopy (Evolution 300, Thermo Fisher, US), and the SOR loading content was determined by high-performance liquid chromatography (HPLC, SPD-M20A, SHIMADZU, Japan).

### 2.4. Synthesis of FeGP@HMON-SNO, LAPGP@HMON-SNO, BQRGP@HMON-SNO SORGP@HMON-SNO

0.58 mL of HMON-SNO (10 mg/mL) dispersion was premixed with 0.1 mL of GP (*C*_Gd_ = 13.1 mM, *C*_GP_ = 10.8 mg/mL) aqueous solutions under ultrasonic conditions, followed by the addition of 0.02-0.32 mL of FeCl_2_, LAP, BQR, or SOR solution (4.0 mg/mL), resulting in mass ratios of ferroptosis agonist/GP at 0.074, 0.148, 0.296 and 0.593. The total volume of the mixtures was set to 1.0 mL, and the mixtures were further stirred for 24 h. Subsequently, the resulting samples were centrifuged and washed at 20000 × g for 15 min at 4.0 °C to obtain FeGP1-5@HMON-SNO, LAPGP1-4@HMON-SNO, BQRGP1-4@HMON-SNO, or SORGP1-4@HMON-SNO. The Fe and Gd loading content in the prepared samples was determined by ICP-OES (iCAP PRO, Thermo Fisher Scientific, US), the LAP and BQR loading content was analyzed using UV-Vis spectroscopy, and the SOR loading content was determined by HPLC.

## 3. Results and Discussion

### 3.1. Synthesis and characterization of the nanoplatforms

**Figure 1:**
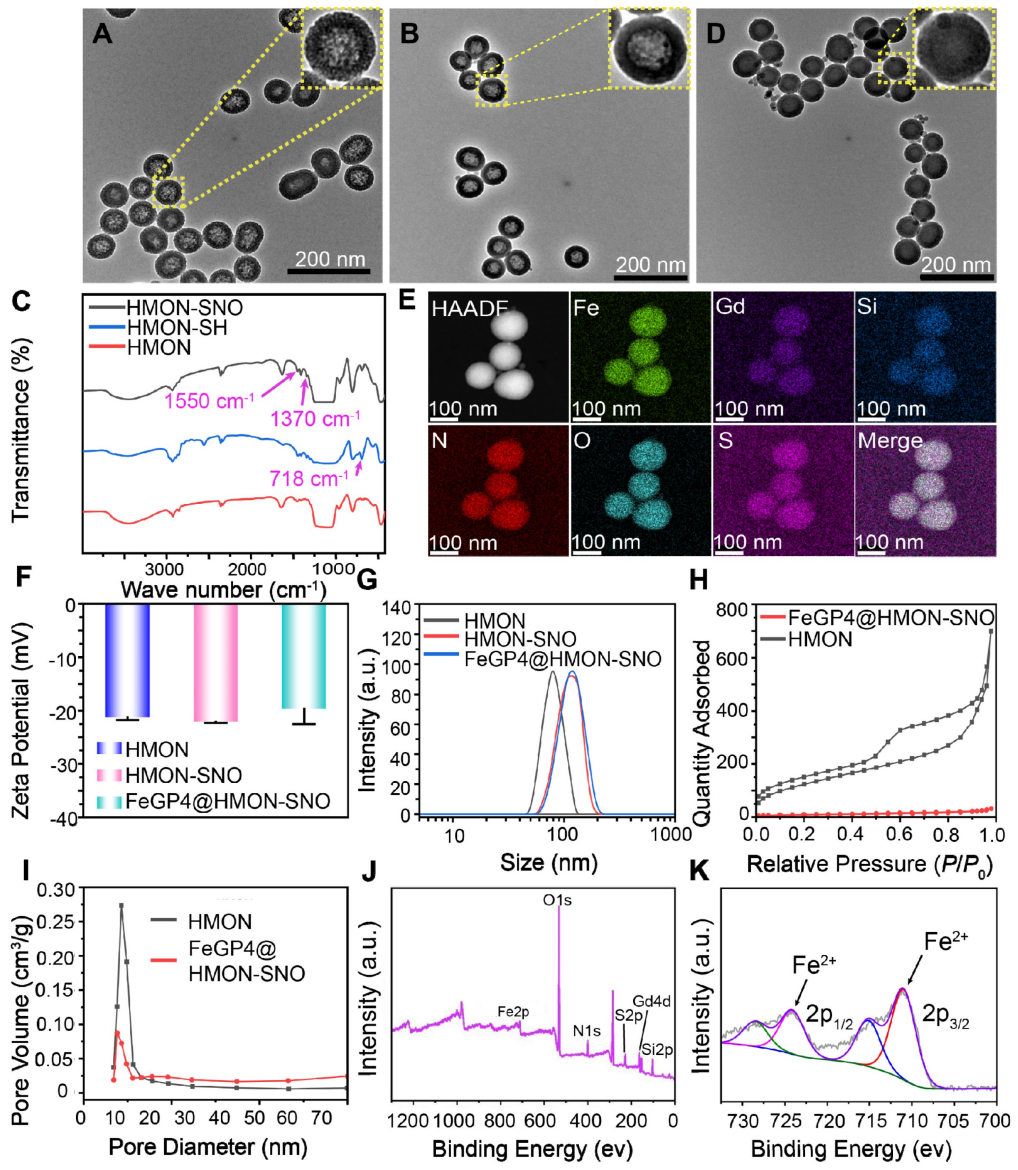
TEM images of HMON (A), HMON-SNO (B), and FeGP4@HMON-SNO (C). (D) Infrared spectrum of HMON, HMON-SH, and HMON-SNO. (E) Elemental mapping of FeGP4@HMON-SNO. (F) zeta potentials and (G) size distributions of HMON, HMON-SH, and HMON-SNO measured by DLS. (H) N_2_ adsorption-desorption isotherms, and (I) pore-size distributions of HMON and FeGP4@HMON-SNO. (J) Full range XPS spectra, and (K) corresponding high-resolution XPS spectra of Fe 2p for FeGP4@HMON-SNO.

The HMON which serving as a versatile drug delivery system, was initially synthesized using an “ammonia-assisted selective etching” approach as outlined in our previous publication 29. To endow HMON with additional therapeutic functions, the HMON nanoparticles underwent post-modification to introduce surface thiol groups, denoted as HMON-SH. Subsequently, these thiol groups within HMON-SH were converted to SNO through nitrosation chemistry involving sodium nitrite 30. Figure 1A-B show transmission electron microscope (TEM) images of HMON and HMON-SNO, respectively. The uniform spherical morphology, notable hollow structure, and excellent monodispersity observed in the prepared HMON and HMON-SNO suggest that the modification with the -SNO group has no impact on the morphological structure or dispersity of HMON. Therefore, the prepared HMON-SNO, with its additional therapeutic functions, is expected to leverage the cavities of HMON-SNO to achieve a CIA strategy, which restricts the aggregation of MRI CAs under high drug loading conditions.

Analysis of the ultraviolet-visible spectroscopy (UV-vis) curve reveals a distinct characteristic band at 336 nm in the HMON-SNO specimens, which is absent in the spectra of HMON (Figure S1). This observation aligns with the typical UV absorption pattern of the GSNO specimen, indicating the successful modification of -SNO onto the HMON-SNO. Figure 1C displays the Fourier-transform infrared spectroscopy (FT-IR) spectra of HMON, HMON-SH, and HMON-SNO, which further confirm the successful synthesis of HMON-SNO. Specifically, compared to the HMON sample, HMON-SH exhibits a new characteristic peak at 718 cm⁻¹ corresponding to the -SH group. The FT-IR spectrum of HMON-SNO reveals peaks at 1370 cm⁻¹ and 1550 cm⁻¹, attributed to the antisymmetric and symmetric stretching vibration absorption bands of the -SNO group, respectively. Both peaks are absent in the spectra of HMON and HMON-SH, indicating the effective incorporation of -SNO groups on the surface of HMON-SNO.

To validate the complete S-nitrosoglutathione modification of HMOS-SH, we performed a colorimetric assay utilizing 5,5'-dithio-bis-2-(nitrobenzoic acid) (DTNB). The reaction of -SH groups with colorless DTNB leads to the formation of colored thionitrobenzoate (TNB), which exhibits a characteristic UV absorption peak 30, 31. Our results indicate that the HMOS-SH group displays a pronounced UV absorption peak at 412 nm, which is indicative of TNB formation. In contrast, no significant absorption was observed in the HMON or HMON-SNO samples, suggesting the absence of -SH groups (Figure S2). These findings collectively confirm the successful and complete S-nitrosoglutathione modification of HMOS-SH. Furthermore, the -SNO content in the HMON-SNO sample was quantified to be 0.223 μM per mg of nanoparticles, as determined using a GSNO standard curve and measured by UV-visible spectrophotometry (Figure S3).

Previously, we have reported GP with high *r*_1_ values of 70.2 ± 1.8 mM^-1^ s^-1^ (1.5 T) 32 and 56.2 ± 1.7 mM^-1^ s^-1^ (3.0 T) 18, respectively, which are considered as a promising *T*_1_-weighted MRI CA. To impart therapeutic functionality to the MRI CAs, ferroptosis agonists such as Fe^2+^, LAP, BOR, or SOR drugs were attempted to be loaded onto GP *via* electrostatic interactions. The synthesis conditions and characterization outcomes for FeGP1-5, LAPGP1-4, BQRGP1-4, and SORGP1-4 are summarized in Tables S1-S4. As expected, significant visible precipitates resulting from extensive uncontrollable aggregation are found for the FeGP1-5, LAPGP3, 4, BQRGP4, and SORGP3, 4 (Table S1-S4 and Figure S4-S7). The maximum DLC (*i.e.*, the mass percentage of the loaded drugs including Fe^2+^, LAP, BOR, or SOR to the GP) for each formulation is 8.4%, 13.3%, 9.6%, and 10.3% respectively. These findings demonstrate that the uncontrollable aggregation observed in drug loading applications of MRI CAs is a common issue that urgently needs to be addressed 33.

To conquer this challenge, we propose a CIA strategy to restrict the aggregation of MRI CAs. Fe^2+^, LAP, BQR, or SOR were tried to be loaded on GP in the hollow core of HMON-SNO. Table S5-S8 summarize the synthesis conditions and characterization results of FeGP1-5@HMON-SNO, LAPGP1-4@HMON-SNO, BQRGP1-4@HMON-SNO, or SORGP1-4@HMON-SNO. Interestingly, there is no precipitation observed in the dispersions of FeGP1-4@HMON-SNO, LAPGP1-4@HMON-SNO, BQRGP1-4@HMON-SNO, or SORGP1-4@HMON-SNO (Figure S8-S11), presenting the controllable ideal aggregation. Importantly, the FeGP4@HMON-SNO, LAPGP4@HMON-SNO, BQRGP4@HMON-SNO, and SORGP4@HMON-SNO without precipitation exhibit a very high DLC (*i.e.*, 25.6, 20.6, 18.7, and 17.4%), which is much higher than that of FeGP4, LAPGP4, BQRGP4, and SORGP4 with serious precipitation (*i.e.*, 7.1%, 13.3%, 9.6%, and 10.3%). This result can be ascribed to the formation of large aggregates within the hollow core of HMON-SNO based on the CIA strategy, which is much larger than the mesopore size of HMON-SNO. Thus, the premature release of various ferroptosis agonists could be effectively impeded. Consequently, the CIA strategy can ensure high DLC while avoiding uncontrollable aggregation for MRI CAs. Based on the results above, we confirmed the feasibility and universality of the proposed CIA strategy to overcome a common issue in drug loading and delivery using MRI contrast agents: the tendency for aggregation and precipitation at high drug loading conditions. Next, we selected the classic Fenton reaction agent Fe^2+^ for subsequent experiments.

Figure 1D illustrates the uniform spherical morphologies and favorable monodispersity of the as-prepared FeGP4@HMON-SNO observed through TEM and scanning electron microscopy (SEM) results (Figure S12). High magnification TEM images reveal the structures of HMON, HMON-SNO, and FeGP4@HMON-SNO nanoparticles. The size and morphology of HMON and HMON-SNO exhibit minimal differences; however, the transmittance of FeGP4@HMON-SNO is significantly lower than that of HMON and HMON-SNO, which can be attributed to the incorporation of FeGP chelates within HMON. The elemental mapping generated through scanning transmission electron microscopy (STEM) illustrates the primary composition within FeGP4@HMON-SNO. Specifically, the Si element originates from HMON, the S is sourced from HMON or -SNO, the O is derived from -SNO or GP, the N comes from -SNO, and the Fe and Gd are attributed to the FeGP chelates (Figure 1E). Furthermore, energy dispersive spectrometer (EDS) analysis further confirms the successful construction of FeGP4@HMON-SNO (Figure S13).

The Zeta potentials of HMON, HMON-SNO, and FeGP4@HMON-SNO are determined as -21.2 ± 0.54, -22.0 ± 0.20, and -19.6 ± 2.91 mV (Figure 1F), respectively, based on the dynamic light scattering (DLS) measurements. It is noted that the presence of the -SNO functional group on HMON promotes resonance between the nitrogen and oxygen atoms, enhancing the electron-donating effect of nitrogen and increasing its electron density. In contrast, the oxygen atom experiences reduced electron density, resulting in a partial negative charge on nitrogen and a partial positive charge on oxygen^34^. Consequently, the -SNO group carries an overall negative charge, leading to a lower zeta potential for HMON-SNO compared to HMON. Moreover, the hydrodynamic diameter (*d*_h_) of HMON (78.4 nm), HMON-SNO (107.6 nm), and FeGP4@HMON-SNO (118.7 nm) increases in turn because of the surface modifications (Figure 1G). The favorable mono-dispersity of prepared nanoparticles also validates by their corresponding result of *d*_h_ determined by DLS. Additionally, the supplemented data on the *d*_h_ of FeGP4@HMON-SNO dispersed in culture medium (DMEM) + 10% fetal bovine serum (FBS) or FBS over 10 days show negligible changes, indicating the exceptional colloidal stability of FeGP4@HMON-SNO due to its negative charges (Figure S14-S15).

The specific surface area (462.3 m²/g) and the well-defined mesoporous structure (pore size ~ 9.4 nm) of HMON were characterized using a standard N_2_ adsorption-desorption method (Figure 1H-I). After the successful modification of -SNO and the loading of FeGP chelates, the specific surface area attenuates to 29.9 m²/g (Figure 1H), and the pore size reduces to approximately 6.5 nm (Figure 1I). Full X-ray photoelectron spectroscopy (XPS) analysis exhibits the characteristic peaks corresponding to Fe 2p, O 1s, N 1s, S 2p, Gd 4d, and Si 2p in the spectrum of FeGP4@HMON-SNO (Figure 1J). The high-resolution XPS spectrum of Fe 2p (Figure 1K) reveals prominent peaks at 710.1 eV (Fe 2p_3/2_) and 724.3 eV (Fe 2p_1/2_), indicating the presence of Fe^2+^ in FeGP4@HMON-SNO.

### 3.2. •OH generation, NO generation, GSH depletion, and GSH-responsive degradation of FeGP4@HMON-SNO

**Figure 2:**
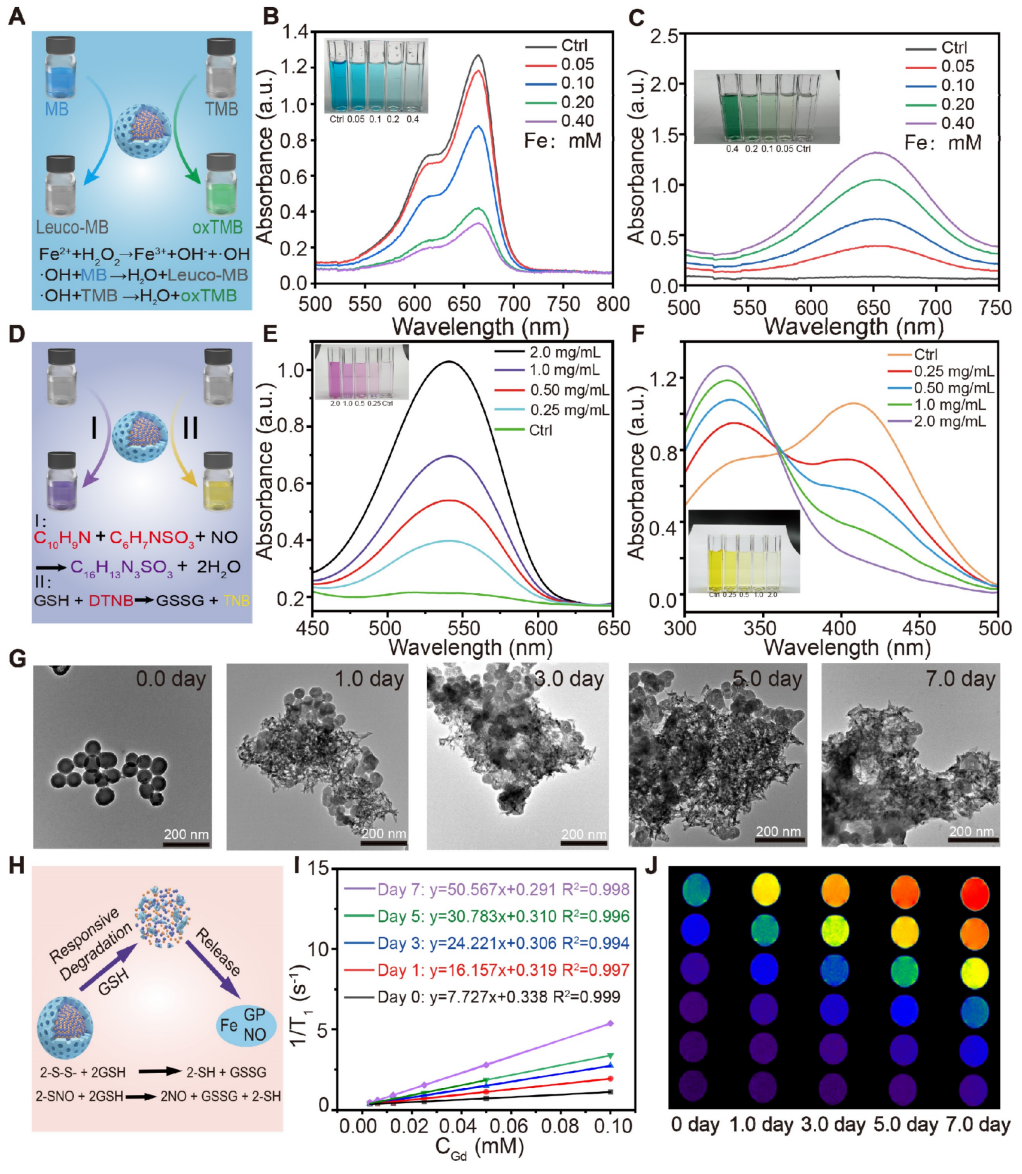
(A) Mechanisms for detection of ●OH generated from FeGP4@HMON-SNO *via* Fenton reaction utilizing the MB assay or TMB assay. UV-vis spectra and photographs (inset) of (B) MB solutions, or (C) TMB solutions incubated with FeGP4@HMON-SNO (C_Fe_ = 0-0.40 mM) in the presence of H_2_O_2_ (2.0 mM) at pH=6.8. (D) Schematic illustration for detection of the NO generation and GSH depletion capability of FeGP4@HMON-SNO utilizing the Griess assay and DTNB assays, respectively. UV-vis spectra and photographs (inset) of (E) Griess agents, and (F) DTNB solutions incubated with FeGP4@HMON-SNO (C_Fe_ = 0-0.40 mM) in the presence of GSH (10 mM). (G) TEM images of the biodegradable FeGP4@HMON-SNO after incubation in PBS containing 10 mM of GSH for 0-7.0 days. (H) Schematic illustration for GSH-responsive degradation of FeGP4@HMON-SNO and the corresponding release of theranostic agents. *T*_1_ relaxation rates plotted as a function of (I) Gd concentrations, and the corresponding (J) *T*_1_-weighted MR images for FeGP4@HMON-SNO solutions measured at a magnetic field of 3.0 T after incubation with 10 mM of GSH for 0-7.0 days.

The *in vitro* generation performance of •OH by FeGP4@HMON-SNO was assessed by monitoring the degradation of methylene blue (MB) in a fading experiment. Given that •OH produced by the Fenton reaction between Fe^2+/3+^ and H_2_O_2_ can oxidize the colored MB back to its colorless form (leuco-MB) (Figure 2A), the UV-vis absorbance of the MB gradually decreases with increasing concentrations of FeGP4@HMON-SNO (Figure 2B). Furthermore, the MB experiment also demonstrates the time-dependent remarkable •OH production capacity of FeGP4@HMON-SNO (Figure S16a).

The production efficiency of •OH was further examined through the quantification of the 3,3',5,5'-tetramethylbenzidine (TMB) measurement. The presence of •OH triggers the oxidation of colorless TMB, resulting in the formation of blue or green oxidized TMB (oxTMB) (Figure 2A). As the feeding concentration of FeGP4@HMON-SNO increases, the UV-vis absorbance of the TMB solution gradually enhances (Figure 2C). In addition, the TMB experiment also reveals the time-dependent generation of •OH, thereby affirming the sustained •OH production capacity of FeGP4@HMON-SNO (Figure S16b). The electron spin resonance (ESR) spectra also exhibit the enhanced characteristic signal of •OH for FeGP4@HMON-SNO when stimulated by GSH. This is attributed to the GSH-responsive degradation of FeGP4@HMON-SNO, leading to the release of more Fe^2+^ to enhance the Fenton catalytic reaction (Figure S17).

NO can be efficiently generated and released from -SNO groups in response to GSH 28, 35. Upon interaction with a GSH solution, NO produced from FeGP4@HMON-SNO can react with the Griess reagent, leading to the formation of a purple product (Figure 2D). The efficiency of NO production was evaluated using Griess reagent measurements (Figure 2E). Minimal absorbance of the Griess reagent at 540 nm is observed for FeGP4@HMON-SNO without GSH stimulation. In contrast, a noticeable increase in the absorption of the Griess reagent is observed with the increasing FeGP4@HMON-SNO concentration at the presence of GSH (10 mM). The temporal evolution of NO production from FeGP4@HMON-SNO in the presence of GSH is further elucidated in Figure S18, demonstrating a rise of the absorbance at 540 nm in correlation with the progression of the reaction time.

Furthermore, the GSH consumption capacity of FeGP4@HMON-SNO was assessed using the 5,5'-dithio-bis-2-(nitrobenzoic acid) (DTNB) assay (Figure 2D). The colorless DTNB solution reacts with GSH, resulting in the formation of a yellow TNB solution. As the concentration of FeGP4@HMON-SNO increases gradually, the color of the TNB solution lightens progressively, and the UV-vis absorption at 412 nm diminishes (Figure 2F), which indicates the efficient depletion abilities of GSH by FeGP4@HMON-SNO. Additionally, the GSH depletion ability of FeGP4@HMON-SNO shows a temporal dependence (Figure S19).

To visually confirm the GSH-responsive degradation, the synthesized FeGP4@HMON-SNO was incubated with PBS containing GSH (10 mM) for different durations, and then the structural changes were monitored by TEM. After the 1st day of degradation, the structure of FeGP4@HMON-SNO shows signs of damage. The structural breakdown and dissociated framework of HMON-SNO become more and more evident as the incubation time extends. After 7 days of GSH incubation, there are hardly any complete spherical nanoparticles left. (Figure 2G). These findings emphasize the significant biodegradation potential of FeGP4@HMON-SNO attributed to the existence of disulfide bonds within the framework that are extremely responsive to GSH.

Figure S20 shows the *in vitro* release behavior of Fe from FeGP4@HMON-SNO and Fe4@HMON-SNO, both in the presence and absence of GSH. The Fe release from both nanoparticles is significantly greater in the presence of GSH, as both are subject to degradation under GSH conditions. In the absence of GSH, the release of Fe from FeGP4@HMON-SNO is slower than that from Fe4@HMON-SNO, attributed to the larger size of the generated FeGP aggregates within the HMON-SNO hollow core, which exceeds the mesopore size of HMON-SNO, thereby limiting premature drug leakage. Conversely, in the presence of GSH, the Fe release from FeGP4@HMON-SNO is substantially higher than that from Fe4@HMON-SNO, owing to the increased Fe loading content (*i.e.*, the mass percentage of loaded Fe^2+^ relative to HMON-SNO, 4.8 %% *vs*. 1.4 %).

As expected, the GSH-triggered release characteristics of both Fe and GP were validated in PBS with or without 10 mM of GSH. Upon GSH-induced HMON-SNO degradation, a notably higher release of Fe (80.2%) or GP (75.1%) within 72 h is observed under the simulated TME condition with GSH, whereas their release does not exceed 20 % under normal physiological conditions without GSH (Figure S21). Furthermore, the release profiles of NO from FeGP4@HMON-SNO under varying concentrations of GSH specifically at 0, 5.0, or 10 mM were assessed (Figure S22). The results demonstrate that FeGP4@HMON-SNO exclusively releases NO in the presence of GSH. Notably, the amount of NO released is positively correlated with both the duration of incubation and the concentration of GSH (Figure S22), highlighting a TME-specific NO release mechanism. This specific GSH-triggered degradation of FeGP4@HMON-SNO leads to the controlled release of Fe, NO, and GP in the TME (Figure 2H), thereby potentially enhancing the theranostic efficacy of ferroptosis and reducing toxic effects on normal tissues and cells.

Based on the GSH-triggered degradation and release ability, the longitudinal/transverse relaxation time (*T*_1_/*T*_2_) of FeGP4@HMON-SNO with various *C*_Gd_ incubated with 10 mM GSH for 0-7.0 days was tested at 3.0 T. The FeGP4@HMON-SNO composite, with its ideal controlled FeGP aggregates, exhibits weak *T*_1_ signals, attributed to its relatively low *r*_1_ value (7.7 mM^-1^ s^-1^) and elevated *r*_2_/*r*_1_ ratio (14.1). This phenomenon arises from the formation of controlled GP aggregates within the HMON-SNO hollow core, which occurs under excessive Fe^2+^ loading. This process potentially leads to an enhancement in the saturation magnetization (*M*s) and corresponding increases in *r*_2_, as well as a reduction in the interaction between water protons and GP, resulting in corresponding decreases in *r*_1_. After incubation with 10 mM GSH to simulate the TME for a duration of 0 to 7 days, the *r*_1_ value of FeGP4@HMON-SNO increases from 7.7 to 50.6 mM^-1^ s^-1^ (Figure 2I), while *r*_2_ values decrease from 109.0 to 79.9 mM^-1^ s^-1^ (Figure S23A), and the *r*_2_/*r*_1_ ratio declines from 14.1 to 1.6 (Figure S23B). The observed increase in *r*_1_ and decrease in the *r*_2_/*r*_1_ ratio indicate a significant enhancement in *T*_1_ MRI signals (Figure 2J). This enhancement is primarily due to the reductive microenvironment-induced degradation of the HMON framework, which facilitates the release of MRI-active GP agents, thereby generating stronger *T*_1_-weighted MRI signals. It is noted that the enhancement in MRI signal responsiveness to the TME closely approximates the efficacy of the GP agents alone, underscoring the potential of GSH-activatable FeGP4@HMON-SNO for high-contrast tumor imaging.

### 3.3. Behaviors of FeGP4@HMON-SNO on cellular and lysosome escape

The uptake capability of FeGP4@HMON-SNO by 4T1 cells was assessed using laser scanning confocal microscopy (LSCM), flow cytometry, and MRI measurements. The FeGP4@HMON-SNO nanoparticles were labeled with rhodamine 6G (R6G) to facilitate the visual observation of their fluorescence. Due to its mesoporous structure and bigger surface area, HMON-SNO can effectively adsorb the small molecule dye R6G through capillary action, both on its surface and within its pore channels. Following multiple washes with deionized water, we isolated R6G-labeled FeGP4@HMON-SNO nanoparticles devoid of free R6G. It is noted that R6G, a water-soluble cationic fluorescent dye, preferentially binds to negatively charged biomolecules, such as DNA, within cells, resulting in red fluorescence observed in the nuclei^36^. However, upon cellular uptake, R6G-labeled FeGP4@HMON-SNO nanoparticles, approximately 118.7 nm in size, are unable to traverse the nuclear pore complex. This limitation results in the accumulation of red fluorescence in the cytoplasm, while the nuclei remain non-fluorescent.

**Figure 3:**
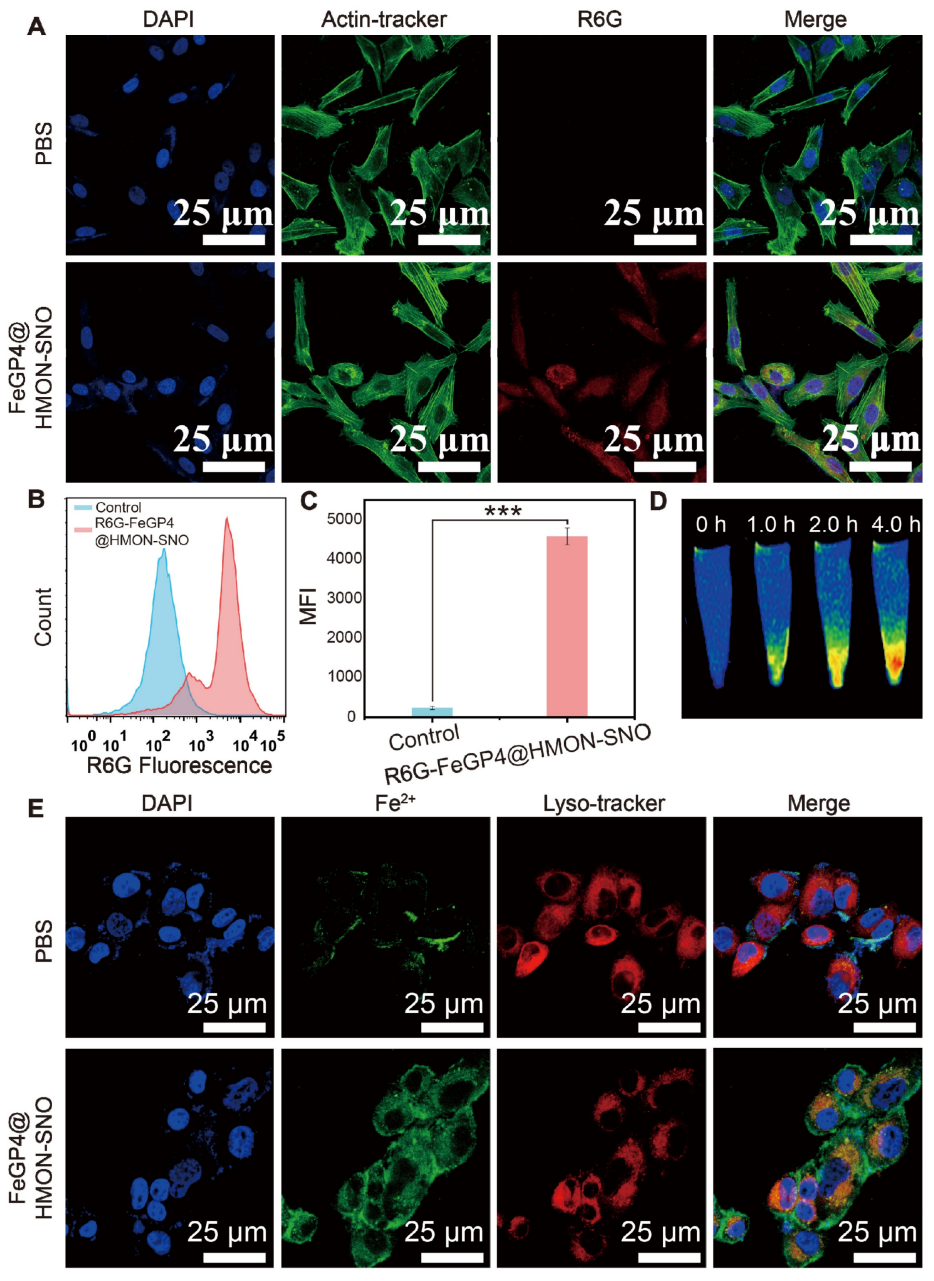
(A) LSCM images of 4T1 cells after incubation with PBS or R6G-labelled FeGP4@HMON-SNO, showing cellular uptake of the nanoparticles. Green fluorescence: Actin-Tracker for cytoskeleton; red fluorescence: R6G for nanoparticles; blue fluorescence: DAPI for nuclei. (B) R6G fluorescence distributions of 4T1 cells incubated with PBS or R6G-labelled FeGP4@HMON-SNO and the (C) corresponding quantitative analysis determined by flow cytometry. Mean ± SD, *n* = 3. *** *P* < 0.001. (D) *T*_1_-MRI images of 4T1 cells after treatment with FeGP4@HMON-SNO, measured on a 3.0 T MRI scanner. (E) LSCM images of 4T1 cells after incubation with FeGP4@HMON-SNO, showing lysosome escape of the nanoparticles. Red fluorescence: Lyso-Tracker for lysosomes; green fluorescence: FeRhoNox-1 for Fe^2+^; blue fluorescence: DAPI for nuclei.

LSCM observations reveal a significantly stronger red fluorescence signal in 4T1 cells treated with R6G-labeled FeGP4@HMON-SNO compared to the control group treated with PBS, indicating effective cellular uptake of the nanoparticles (Figure 3A). Meanwhile, the images confirm that, after 4.0 h of incubation, the predominant red fluorescence is localized in the cytoplasm, with minimal R6G detected in the nuclei, likely attributable to nanoparticle degradation. These results highlight the stability of R6G-labeled FeGP4@HMON-SNO and suggest that the nanoparticles enter cells via the endocytic pathway, as evidenced by the distribution of the R6G signal. Figure S24 shows LSCM images of R6G-labeled FeGP4@HMON-SNO incubated with 4T1 cells for varying periods, demonstrating the time-dependent endocytosis behavior of the nanoparticles. The intracellular red fluorescence intensity increases over time, peaking at 4.0 h. Furthermore, both michigan cancer foundation-7 (MCF7) and human gastric cancer cell (HGC27) treated with R6G-labeled FeGP4@HMON-SNO exhibit notable fluorescence signals, further suggesting robust uptake capabilities of FeGP4@HMON-SNO by human cancer cells (Figure S25).

Consistent with the LSCM observations, the flow cytometry analysis for the fluorescence intensity of 4T1 cells treated with FeGP4@HMON-SNO (Figure 3B-C) exhibits a significant enhancement compared with the control group (*** *P* < 0.001), suggesting the effective endocytosis of FeGP4@HMON-SNO by 4T1 cells. Figure 3D illustrates the *T*_1_-weighted MRI of 4T1 cells post-treatment with FeGP4@HMON-SNO for varying time intervals, captured under 3.0 T of magnetic field. 4T1 cells that successfully uptake the nanoparticles continue to exhibit *T*_1_ MRI signals. Furthermore, prolonged incubation time correlates with increased nanoparticle uptake, which further enhances the *T*_1_ MRI signals. Over the time of 0-4.0 h, the MRI signal intensity gradually increases, demonstrating a time-dependent increase of the endocytosis. The Bio-TEM image shows the distribution of FeGP4@HMON-SNO both within and outside the lysosomes, providing additional evidence of the endocytosis of nanoparticles by 4T1 cells (Figure S26).

Furthermore, the 4T1 cells after treatment with FeGP4@HMON-SNO nanoparticles and staining with FeRhoNox-1 (green fluorescence for Fe^2+^) and Lyso-Tracker (red fluorescence for lysosomes) were visualized using LSCM (Figure 3E). Notably, a distinct segregation between red and green fluorescent areas is observed, indicating the efficient lysosomal escape of FeGP4@HMON-SNO. The quantitative analysis of lysosomal co-localization with Fe^2+^ in 4T1 cells following treatment with FeGP4@HMON-SNO at various time points is illustrated in Figure S27A-D. After 1.0 h of incubation, the fluorescence co-localization rate of FeGP4@HMON-SNO with lysosomes exceeded 70%. However, this rate declines to 54% at 2.0 h and further decreases to 20% at 4.0 h. These findings suggest that FeGP4@HMON-SNO experiences substantial lysosomal escape within the cells over time. The lysosomal escape of FeGP4@HMON-SNO is facilitated by the proton sponge effect. The presence of silanol groups in HMON-SNO and carboxyl groups in GP allows these compounds to function as proton sponges, effectively sequestering a substantial number of protons within lysosomes. This proton uptake disrupts the osmotic pressure balance, leading to lysosomal swelling and eventual rupture. This characteristic of lysosomal escape is critical for subsequent tumor ferroptosis treatment, as it ensures the availability of sufficient catalytic substrate (Fe^2+^).

### 3.4. *In vitro* verification of ferroptosis triggered by FeGP4@HMON-SNO

**Figure 4:**
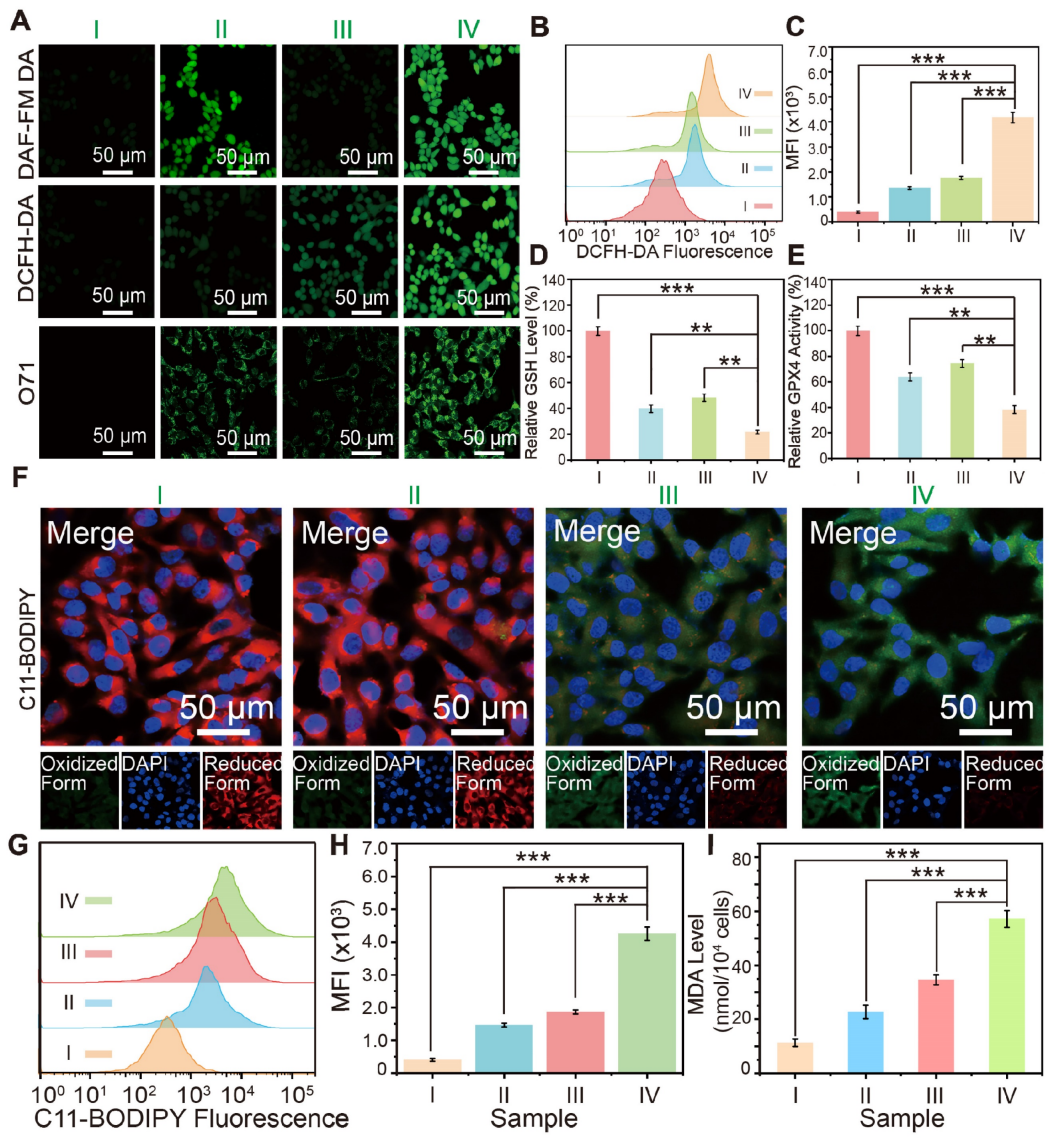
(A) LSCM images of 4T1 cells incubated with PBS (control, I), HMON-SNO (Ⅱ), FeGP4@HMON (Ⅲ), or FeGP4@HMON-SNO (Ⅳ), and stained with DCFH-DA (for ROS generation measurement), DAF-FM DA (for NO generation), and O71 probe (for ONOO^-^ generation). (B) DCF fluorescence distributions, and the (C) corresponding quantitative analysis of 4T1 cells determined by flow cytometry for the intracellular ROS generation assay after the aforementioned treatments (*i.e.*, groups I-IV). (D) GSH levels, and (E) the GPX4 activity in 4T1 cells after treatments of the group I-IV. Mean ± SD, *n* = 3. **P < 0.01, ***P< 0.001. (F) LPO fluorescent images of 4T1 cells after various treatments and staining with C11-BODIPY^581/591^. (G) C11-BODIPY green fluorescence distributions, and the (H) corresponding quantitative analysis of 4T1 cells after treatments of the group I-IV, measured by flow cytometry. Mean ± SD, *n* = 3. **P < 0.01, ***P < 0.001. (I) The MDA level of 4T1 cells after different treatments.

To investigate the production of NO, ROS, and ONOO^-^ and elucidate the mechanism underlying the potent ferroptosis effect in tumor cells, a series of cellular staining assays were conducted (Figure 4A). DAF-FM DA was utilized as a fluorescent probe to quantify intracellular NO levels. It was observed that 4T1 cells treated with PBS or FeGP4@HMON exhibit a weak fluorescence signal of NO, whereas both the HMON-SNO and FeGP4@HMON-SNO groups display a substantial increase in NO production, as evidenced by a notable rise of the fluorescence intensity (first row of Figure 4A). When 4T1 cells treated with FeGP4@HMON-SNO were pretreated with the GSH inhibitor RSL3, there was a significant decrease in the green fluorescent signal, indicating a reduction in NO release (Figure S28). This phenomenon can be attributed to the -SNO group on the surface of HMON, which reacts with GSH in tumor cells, leading to the generation of NO.

Elevated intracellular NO levels have been documented to trigger mitochondrial dysfunction and suppress mitochondrial respiration, resulting in a reduction in intracellular O_2_ consumption, effectively acting as O_2_ economizers 37. LSCM images of 4T1 cells after treatment with PBS, HMON-SNO, FeGP4@HMON, or FeGP4@HMON-SNO and staining with the [Ru(DPP)_3_]Cl_2_ probe show the visualization of intracellular O_2_ level (Figure S29). The fluorescence intensity serves as a measurement of cellular O_2_ content, with higher intensity correlating to lower O_2_ levels. Consistent with expectations, 4T1 cells treated with FeGP4@HMON-SNO exhibit minimal red fluorescence compared with the PBS or FeGP4@HMON group, which indicates the elevated levels of O_2_ within the cells due to the inhibitory impact of NO on mitochondrial respiration.

Subsequently, an evaluation of ROS generation efficacy in 4T1 cells following various treatments was conducted using the fluorescence probe 2',7'-dichlorofluorescein diacetate (DCFH-DA). Based on the LSCM images (second row of Figure 4A), minimal green fluorescence of ROS is observed in the PBS and HMON-SNO groups, meanwhile, significantly enhanced fluorescence signals are observed in 4T1 cells treated with FeGP4@HMON and FeGP4@HMON-SNO. Particularly, the FeGP4@HMON-SNO group exhibits a notably stronger ROS signal compared with the FeGP4@HMON group. This discrepancy may be attributed to the heightened intracellular NO levels coupled with mitochondrial dysfunction, leading to the generation of mitochondrial ROS 38. The powerful ROS production in 4T1 cells induced by FeGP4@HMON-SNO is further confirmed through flow cytometry analysis of ROS fluorescence (Figure 4B-C), providing additional evidence of the amplified impact of this treatment on ROS generation.

Based on the cellular NO and ROS generation, the *in vitro* production of ONOO^-^ induced by the cascade reaction between NO and ROS was further examined using LSCM with the ONOO^-^ fluorescent probe O71 39. Notably, the distinct green fluorescence indicating the presence of ONOO^-^ is solely detected in FeGP4@HMON-SNO (third row of Figure 4A), which is probably attributed to the interaction between elevated ROS generated by the Fenton reaction and NO from -SNO, thereby resulting in the substantial production of ONOO^-^.

Figure S30 shows the LSCM images of 4T1 cells after various treatments and staining with Thiol-Tracker Violet dyes (a GSH-specific probe). Intense green fluorescence of GSH is found for the PBS group, and reduced fluorescence is observed for the groups of HMON-SNO and FeGP4@HMON. However, the FeGP4@HMON-SNO group exhibits minimal green fluorescence, which offers direct evidence of strong GSH depletion. The quantitative assessment of GSH levels in 4T1 cells conducted using an Assay Kit technique yields similar results (Figure 4D).

The notable GSH depletion capacity is primarily attributed to the presence of -S-S and -SNO bonds within FeGP4@HMON-SNO. The consumption of GSH can induce the deactivation of GPX4, consequently triggering lipid peroxidation and subsequent ferroptosis 40. As shown in Figure 4E, the FeGP4@HMON-SNO group demonstrates significantly reduced GPX4 activity compared with the PBS, HMON-SNO, and FeGP4@HMON groups (***P<0.001, **P<0.01). Clearly, the treatment with FeGP4@HMON-SNO accompanied by the marked GSH depletion and subsequent GPX4 inactivation is expected to promote the LPO accumulation and ferroptosis of tumor cells.

In addition to generating toxic RNS through the reaction of NO and ROS, the release of NO from FeGP@HMON-SNO in response to a GSH-enriched TME is anticipated to induce mitochondrial dysfunction and inhibit cellular respiration. This disruption contributes to a reversal of tumor hypoxia and subsequent downregulation of HIF-1α, which is typically elevated due to oxygen conservation associated with respiratory depression 36. Consequently, this cascade of events reduces the accumulation of lipid droplets (LDs), which act as storage sites for free fatty acids, while promoting the release of free polyunsaturated fatty acids (PUFAs)^41^. The increased release of PUFAs enhances LPO accumulation, ultimately elevating the level of ferroptosis.

The level of lipid droplets in 4T1 tumor cells after various treatments was assessed by the Oil Red O staining. Analysis of the lipid droplet staining images and the corresponding quantitative data obtained from 4T1 cells treated by different groups (Figure S31) reveals minimum lipid droplet storage in the FeGP4@HMON-SNO group, compared with that of the FeGP4@HMON, HMON-SNO, and PBS groups. This result indicates that our FeGP4@HMON-SNO can cause a significant reduction of lipid droplets, which promotes the following LPO accumulation and ferroptosis of tumor cells.

The LPO probe C11-BODIPY^581/591^ was employed to monitor the cellular LPO levels after different treatments. As shown in Figure 4F, in comparison with the PBS and HMON-SNO groups, the FeGP4@HMON and FeGP4@HMON-SNO groups present much stronger green fluorescence and weaker red fluorescence of 4T1 cells due to their strong ●OH generation ability based on the Fenton reaction. In addition, compared with the FeGP4@HMON group, the FeGP4@HMON-SNO group displays stronger green fluorescence and weaker red fluorescence because of the NO-promoted LPO accumulation within cancer cells.

Figure 4G-H show the distributions of green fluorescence from C11-BODIPY and the quantitative assessment of intracellular LPO generation in 4T1 cells, determined through flow cytometry. 4T1 cells treated with FeGP4@HMON-SNO exhibit a substantial increase in LPO production, significantly exceeding the LPO levels observed in the PBS, HMON-SNO, and FeGP4@HMON groups (***P<0.001).

Given that malondialdehyde (MDA) is a key product of lipid peroxidation, the MDA levels in 4T1 cells after different treatments were further evaluated using an MDA kit (Figure 4I). As anticipated, 4T1 cells treated with FeGP4@HMON-SNO display notably higher MDA content compared to the other groups, indicating a significant production of MDA due to the elevated LPO levels induced by FeGP4@HMON-SNO (***P<0.001). These outcomes underscore the considerable potential of FeGP4@HMON-SNO for effective ferroptosis of tumor cells.

### 3.5. *In vitro* antitumor efficacy of FeGP4@HMON-SNO based on ferroptosis-gas synergistic therapy

**Figure 5:**
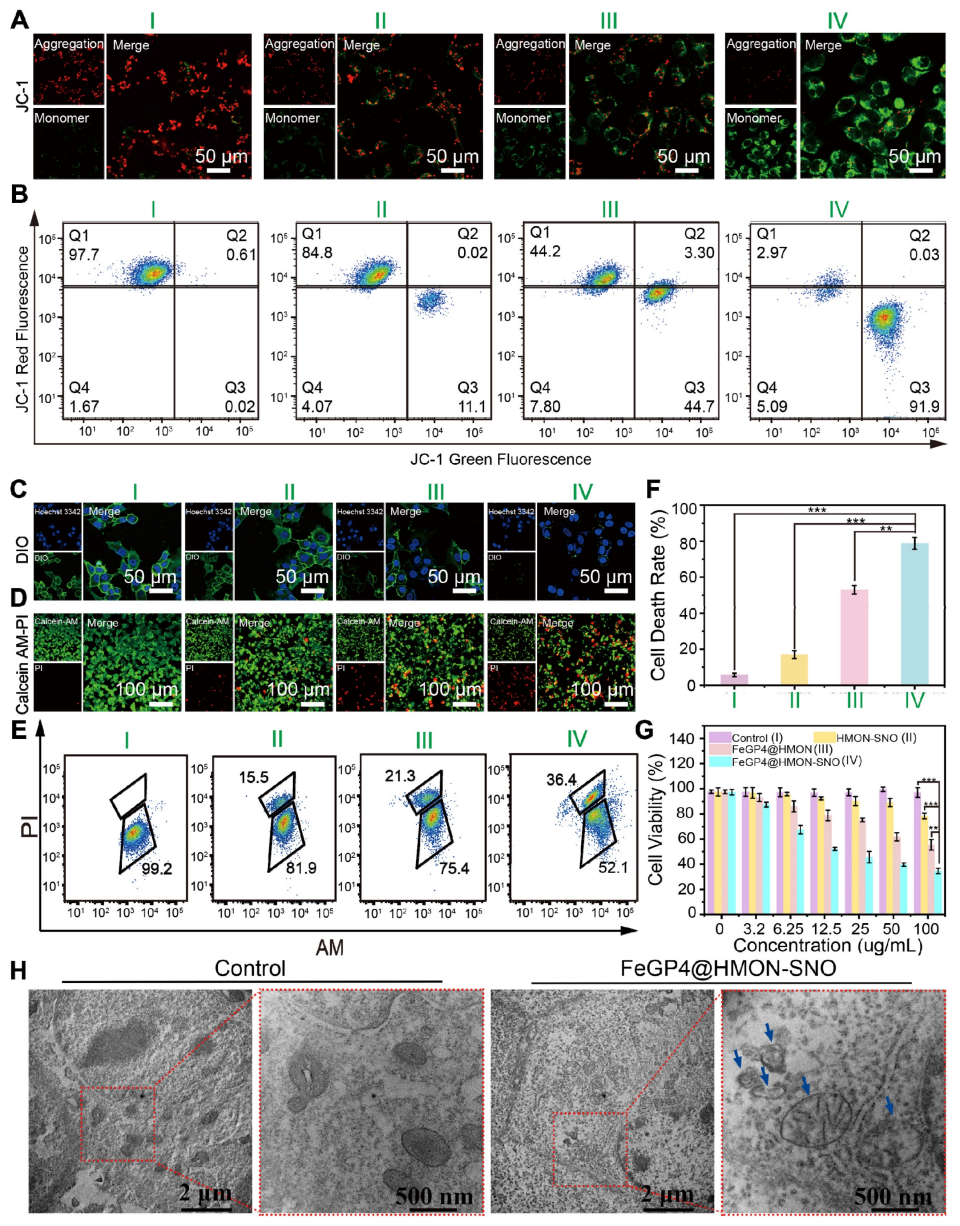
(A) LSCM images of 4T1 cells after treatments with PBS (I), HMON-SNO (II), FeGP4@HMON (III), or FeGP4@HMON-SNO (IV), and staining with JC-1 dyes. Red fluorescence: JC-1 aggregates for healthy mitochondria with normal MMP. Green fluorescence: JC-1 monomers for damaged mitochondria with declined MMP. (B) Flow cytometry quantification of JC-1-labeled 4T1 cells treated with the above-mentioned formulations of I-IV. (C) LSCM images of 4T1 cells after the above-mentioned treatments I-IV, and staining with DIO probe to show the integrality of cells membrane. The decreased green fluorescence indicates damage to the cell membrane. (D) LSCM images of AM/PI co-stained 4T1 cells after the above-mentioned treatments I-IV. Green fluorescence: calcein-acetoxymethyl (Calcein-AM) for live cells; red fluorescence: propidium iodide (PI) for dead cells. (E) Live/Dead 4T1 cells after different treatments and staining of AM/PI measured by flow cytometry. (F) The corresponding quantitative analysis of cell death rate determined by the above flow cytometry analysis. Mean ± SD, *n* = 3. ** *P* < 0.01, *** *P* < 0.001. (G) Cell viability of 4T1 cells after various treatments for 24 h. (H) Bio-TEM images of 4T1 cells treated with PBS or FeGP4@HMON-SNO for 24 h.

Figure 5A depicts LSCM images of 4T1 cells following treatment with PBS, HMON-SNO, FeGP4@HMON, or FeGP4@HMON-SNO, and subsequent staining with JC-1 dyes. The PBS group shows strong red fluoresce from the JC-1 aggregate, indicating the healthy mitochondria of the 4T1 cells. The HMON-SNO group exhibits weak green fluorescence from the JC-1 monomer due to minimal mitochondrial damage, attributed to the inhibitory impact of NO on mitochondria. The FeGP4@HMON group displays evident green fluorescence of the JC-1 monomer, indicating the mitochondrial membrane potential (MMP) dissipation and ensuing mitochondrial impairment. Remarkably, 4T1 cancer cells exposed to FeGP4@HMON-SNO exhibit the most substantial level of mitochondrial injury, characterized by a notably decreased mitochondrial membrane potential resulting from heightened oxidative stress induced by simultaneously elevated levels of ROS and NO. Flow cytometry analysis utilizing the JC-1 Green and Red Fluorescence double staining techniques (Figure 5B) further validates the severe mitochondrial impairments caused by FeGP4@HMON-SNO with 91.9% of cells exhibiting low MMP, which is much higher than that of HMON-SNO (11.1%) and FeGP4@HMON (44.7%).

Figure S32 shows the mitochondrial distribution in 4T1 cells subjected to various treatments and stained with the Mito-Tracker Red probe. The PBS group displays healthy mitochondrial integrity, the HMON-SNO and FeGP4@HMON groups show obvious mitochondrial damage, and the FeGP4@HMON-SNO group presents the most serious mitochondrial impairment.

Cytochrome c oxidase (CCO) is a crucial enzyme in the mitochondrial electron transport chain that catalyzes the reduction of oxygen. NO can bind to CCO, inhibiting mitochondrial function and affecting cellular energy metabolism. Figure S33A illustrates the two-dimensional binding mode of NO with CCO, highlighting hydrogen bonds (green dashed lines) and hydrophobic interactions (red dashed lines and gear shapes). Figure S33B shows the three-dimensional binding mode, where NO stably binds within a cavity formed by the amino acids HIS291, ASP369, HIS368, TRP236, and the heme a3 molecule. Further analysis reveals that NO forms four hydrogen bonds with surrounding amino acids and engages in hydrophobic interactions, enhancing its stable binding to CCO.

Specifically, the oxygen atom of NO forms a hydrogen bond with heme a3, while the nitrogen atom forms three hydrogen bonds with HIS291 and ASP369. Additionally, hydrophobic interactions with two surrounding amino acids further increase the affinity between NO and CCO. Table S9 shows a binding energy of -5.755 kcal/mol between NO and CCO, indicating a strong and stable interaction. These results also suggest that NO can inhibit mitochondrial activity by binding to CCO, thereby improving oxygen content in the TME.

Similar results are found in the assessment of cell membrane integrality stained with DIO (Figure 5C, Figure S34). The membranes of 4T1 cells treated with PBS maintain their intact morphology, whereas varying degrees of membrane disruption are evident in all nanoparticle groups. The FeGP4@HMON-SNO group displays nearly complete membrane rupture, which can be attributed to the ferroptosis-gas synergistic therapeutic effect.

To visually evaluate the efficacy of 4T1 cell eradication across various treatments, a co-staining assay using calcein AM (for live cells)/propidium iodide (PI, for dead cells) was conducted (Figure 5D). The FeGP4@HMON-SNO group displays a pronounced red fluorescence signal (an indicator of dead cells) alongside a minimal green fluorescence signal (live cells). However, the HMON-SNO and FeGP4@HMON groups still present a high proportion of live cells, which underscores that the enhanced anticancer capability stems from the ferroptosis-gas synergistic therapeutic effect. The associated flow cytometry analysis (Figure 5E) and its quantitative evaluation (Figure 5F) distinctly reveal a markedly increased cell death rate for the FeGP4@HMON-SNO group compared with the other three groups, which further indicates the powerful therapeutic effect of FeGP4@HMON-SNO against tumor cells. Figure 5G shows the cell viability of 4T1 cells evaluated using the MTT assay after 24 h of treatments. Compared to the PBS control group, the survival rates of 4T1 cells treated with HMON-SNO and FeGP4@HMON moderately decrease to 78.4% and 55.4%, respectively, which can be attributed to the specific release of NO or Fe^2+^ accompanying the antitumor activity. Notably, the FeGP4@HMON-SNO group exhibits significantly reduced cell viability (34.6%) compared to the other groups at equivalent concentrations, aligning well with the aforementioned AM/PI staining data. This finding demonstrates the synergistic effect of ferroptosis and NO gas therapy in killing 4T1 cancer cells. Additionally, the cell survival rates of MCF7 (30.7%) and HGC27 (30.1%), determined by the MTT assay, are closely aligned with the survival rate observed in 4T1 cells (34.6%) at a concentration of 100 μg/mL of FeGP4@HMON-SNO. These results also suggest that FeGP4@HMON-SNO exhibits notable cytotoxicity against both MCF7 and HGC27 cells (Figure S35).

Figure 5H presents the bio-TEM images of 4T1 cells treated with PBS (control) or FeGP4@HMON-SNO for 24 h. 4T1 cells treated with PBS demonstrate intact organelle structures and morphology. However, 4T1 cells treated with FeGP4@HMON-SNO exhibit mitochondrial damage, evidenced by cristae loss and heightened permeability. These changes are significant indicators of ferroptosis in 4T1 cancer cells induced by FeGP4@HMON-SNO.

Due to the capacity of ROS to oxidize proteins and lipids, as well as induce DNA damage in tumor cells, the extent of DNA damage in different treatment groups was assessed through immunofluorescent staining with γ-H2AX (Figure S36). It is evident that 4T1 cells subjected to FeGP4@HMON-SNO exhibit notable DNA damage in comparison to those treated with PBS, HMON-SNO, or FeGP4@HMON. Furthermore, 4T1 cells were stained with the Azide 488 probe to evaluate levels of cell proliferation. The decreased fluorescence intensity in the FeGP4@HMON-SNO group indicates suppressed cell proliferation (Figure S37). These results strongly support the claim that the ferroptosis-gas synergistic therapy initiated by FeGP4@HMON-SNO can effectively inhibit tumor cell proliferation *via* ferroptosis and apoptosis of tumor cells.

In cancer therapy, the emergence of ferroptosis and NO gas therapies, facilitated by their inducers or donors, has opened new avenues for targeted treatment. Nonetheless, these strategies often encounter significant challenges, including rapid clearance, non-specific distribution, and off-target toxicity, which may limit their therapeutic efficacy. This study introduces a CIA strategy that exhibits substantial drug-loading capacities for synergistic ferroptosis and gas therapy, potentially addressing these limitations.

### 3.6. T_1_-weighted MRI performance and biodistribution analysis *in vivo*

Prior to evaluate the MRI efficacy of FeGP4@HMON-SNO at tumors, the blood circulation half-life was determined to assess the time point with the maximum MRI signal at tumor sites. Figure S38 depicts the pharmacokinetics of FeGP4@HMON-SNO in 4T1 tumor-bearing mice, showing the blood Gd concentration measured *via* ICP-OES at different time points post intravenous (*i.v.*) administration. The blood circulating half-life (*t*_1/2_) of FeGP4@HMON-SNO is determined to be 5.6 h.

**Figure 6:**
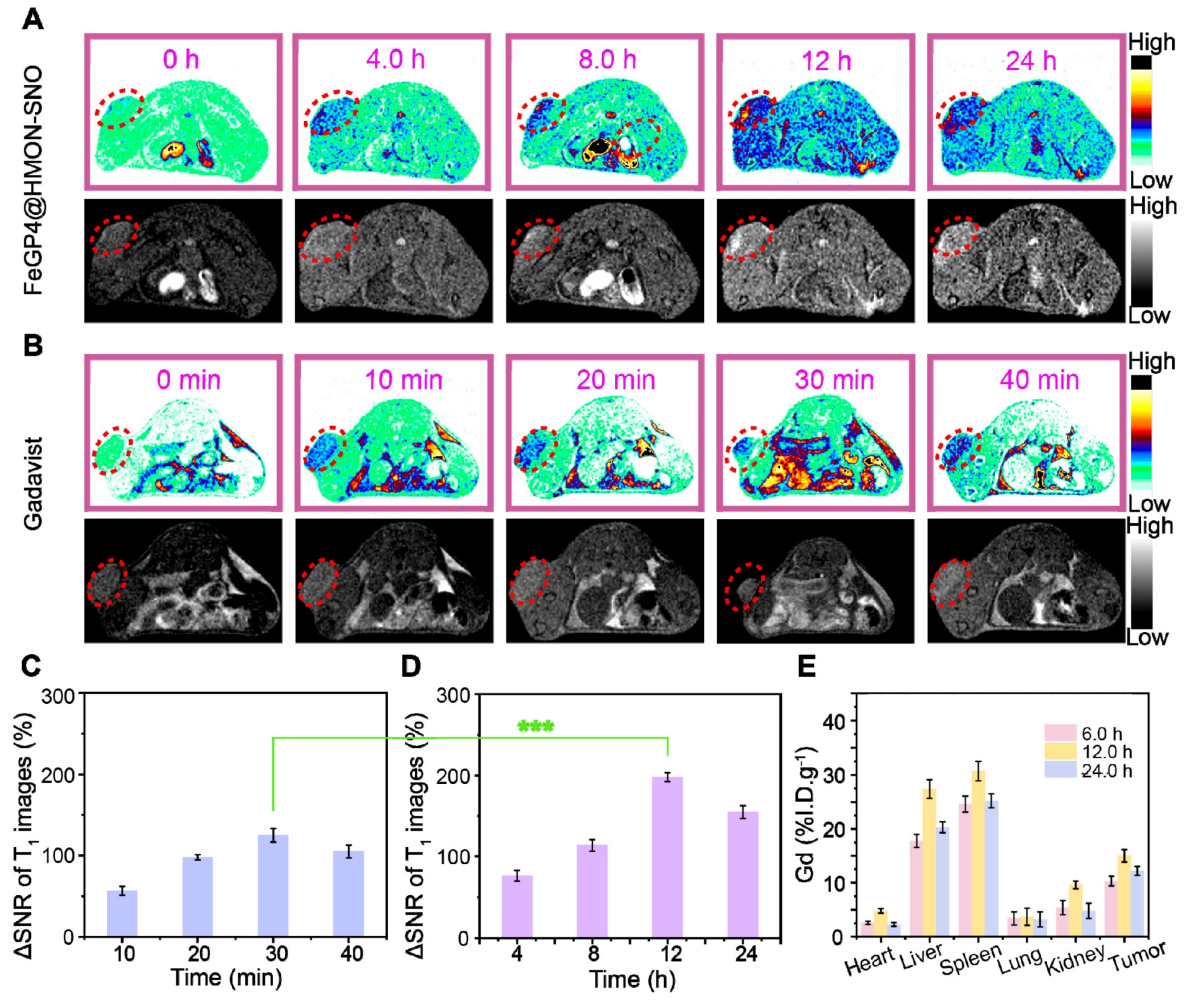
(A) *T*_1_-weighted MR images of 4T1 tumor-bearing mice before and after injection with FeGP4@HMON-SNO (*C*_Gd_ = 5.0 mg/kg, C_Nanoparticle_ = 13.0 mg/kg), or (B) commercial Gadavist^®^ (*C*_Gd_ = 5.0 mg/kg, C_Nanoparticle_ = 13.0 mg/kg) at different time intervals. Magnetic field = 7.0 T. (C) Quantitative data for the tumor *T*_1_ MR images post-injection of commercial Gadavist^®^, or (D) FeGP4@HMON-SNO. Mean ± SD, *n* = 3. *** *P* < 0.001. (E) Biodistributions of Gd in major organs and tumors of mice at 6.0-24 h post-injection (*i.v.*) of FeGP4@HMON-SNO (Gd dosage = 5.0 mg/kg, C_Nanoparticle_ = 13.0 mg/kg). Mean ± SD, *n* = 3.

Figure 6A, B illustrates the *T*_1_-weighted MRI images of 4T1 tumor-bearing mice post *i.v.* injection of the commercially available Gadavist^®^ or FeGP4@HMON-SNO. In the FeGP4@HMON-SNO group, a distinct enhancement of the MRI signal at tumor sites is observed over time, reaching peak brightness at 12 h. This augmentation can be ascribed to the substantial accumulation of FeGP4@HMON-SNO in tumors, and the sustained release of GP prompted by the GSH-responsive HMON degradation within the TME. Gadavist^®^ undergoes rapid renal excretion due to its low molecular weight (*M*_w_ = 604.7). The MRI signal of tumors reaches its zenith at 30 min post-administration, followed by a decline of the signal from 40 min. Notably, the MRI signal of tumors treated with FeGP4@HMON-SNO is significantly stronger than that of Gadavist^®^.

Subsequently, the signal-to-noise ratio (SNR) of the tumor after *i.v.* injection of contrast agent, and the ΔSNR were quantified by the following formula (1) and (2) 42.

SNR = SI_mean_/SD_noise_ (1)

ΔSNR = (SNR_post_ - SNR_pre_)/SNR_pre_ × 100% (2)

Figure 6C-D demonstrate the substantial enhancement of MRI contrast by FeGP4@HMON-SNO than that of Gadavist^®^. The maximum ΔSNR of FeGP4@HMON-SNO is 198.2 ± 5.5% at 12 h post-injection, which is much higher than that of Gadavist (*i.e.*, 122.7 ± 8.3% at 30 min post-injection) (***P<0.001). The MRI signals remain robust even at 24 h post-injection, indicating the prolonged retention of FeGP4@HMON-SNO. That's because the presence of GSH triggers the degradation of HMON-SNO, leading to the specific and sustained release of GP, which ultimately maintains enhanced and persistent MRI signals at the tumor site. Consequently, 4T1 tumor-bearing mice continue to exhibit high tumor brightness 24 h after the intravenous injection of our nanomedicine. These MRI results confirm the superior efficacy of FeGP4@HMON-SNO on real-time monitoring of its *in vivo* delivery, which thereby provides precise guidance for *in vivo* therapeutic interventions.

To further elucidate the high accumulation of FeGP4@HMON-SNO in tumors, the concentrations of Gd and Fe in various organs and tumors were quantified using ICP-OES, enabling a comprehensive exploration of the *in vivo* biodistribution of Gd and Fe. The results demonstrate that the accumulation of Gd in tumor tissues peaks at 12 h post-injection (15.0 ± 1.2 %I.D./g), then gradually decreases to 12.2 ± 0.8 %I.D./g at 24 h (Figure 6E). Similarly, the enrichment of Fe in tumor tissues also reaches its peak at 12 h (17.2 ± 0.8 %I.D./g), followed by a decline to 13.6 ± 1.2 %I.D./g at 24 h (Figure S39). These findings are consistent with the previously discussed circulation half-life and *in vivo* MRI results. Furthermore, it has been observed that the administration of nanomedicines *via* the tail vein in mice leads to significant accumulation of Gd or Fe in the liver and spleen, while their distribution in the heart and kidneys remains minimal. This distribution pattern is predominantly influenced by the activity of the reticuloendothelial system (RES). Moreover, after 24 h of treatment, a notable decrease in the accumulation of FeGP4@HMON-SNO in both the liver and spleen is observed. This finding suggests that FeGP4@HMON-SNO may undergo metabolic processes and subsequent excretion, which could potentially reduce the risk of toxicity associated with its administration.

### 3.7. Evaluation of antitumor efficacy and biosafety *in vivo*

**Figure 7:**
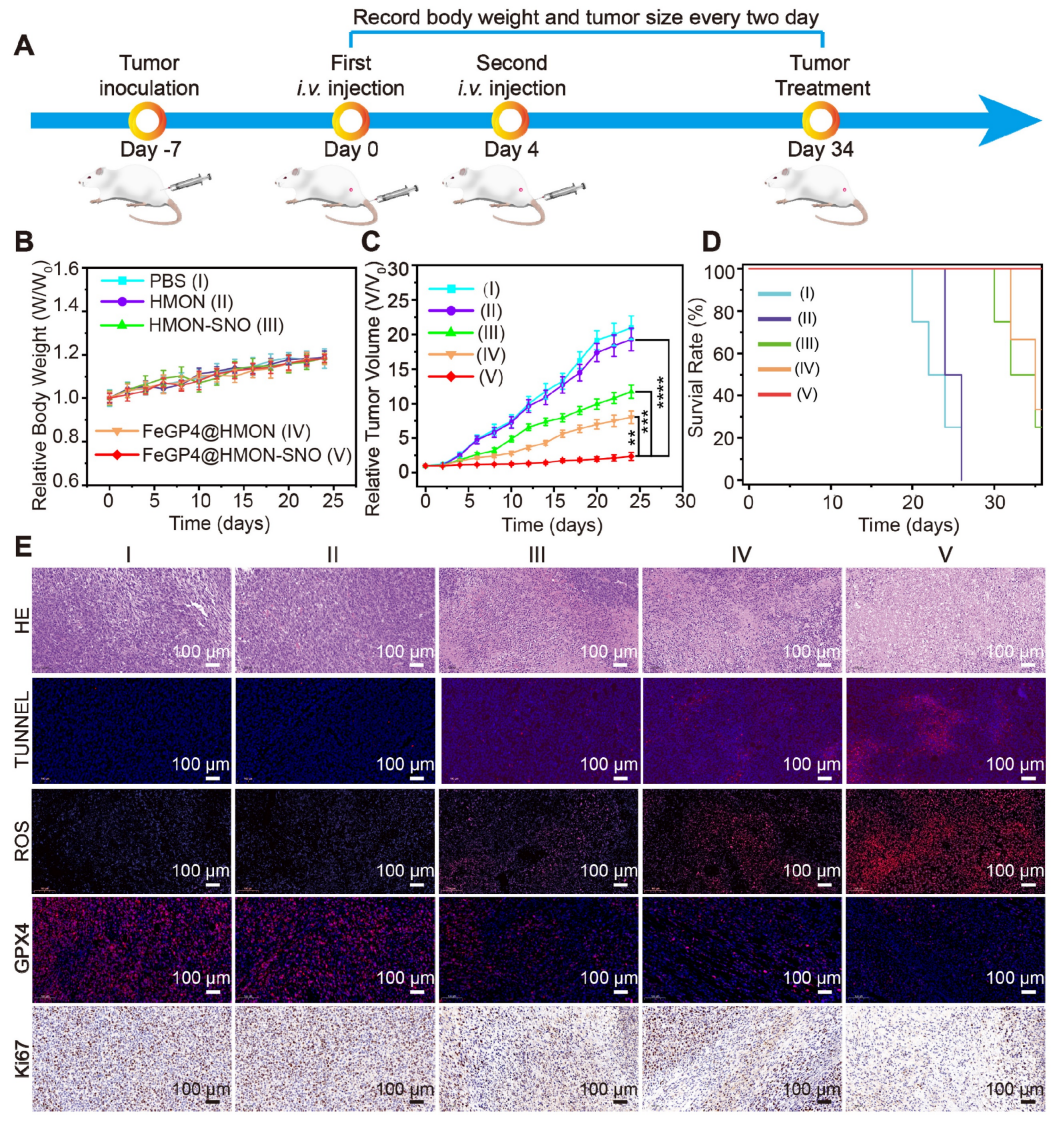
*In vivo* therapeutic performance of FeGP4@HMON-SNO. (A) Schematic illustration for the schedule of tumor inoculation and treatments (100 μL, Fe dosage of 5.0 mg/kg, or an equivalent nanoparticle dosage of 13.0 mg/kg). (B) Curves for the relative body weights, (C) tumor growth, and (D) survival rates of tumor-bearing mice treated with saline (I), HMON (II), HMON-SNO (III), FeGP4@HMON (IV), or FeGP4@HMON-SNO (V). Mean ± SD, *n* = 5. ** *P* < 0.01, *** *P* < 0.001 and ****P < 0.0001. (E) Histological observation of the tumors with staining of H&E, TUNEL, ROS, GPX4, or Ki67 after the above-mentioned treatments.

Given the molecular characteristics of 4T1 tumors, which exhibit similarities to human breast cancers, and the ease and rapidity of establishing these tumors due to the aggressive nature and rapid growth of 4T1 cancer cells, we selected the subcutaneous 4T1 tumor model for the subsequent* in vivo* evaluation of the anti-tumor efficacy of FeGP4@HMON-SNO (Figure 7A). Throughout the entire treatment duration, the 4T1 tumor mice in all groups showed a slight increase in body weight (Figure 7B), indicating that our nanomedical formulation had minimal adverse effects on the normal growth of the mice. Analysis of the tumor growth curves^43^, ^44^ (Figure 7C) reveals significant differences in tumor growth inhibition. The PBS (control) or HMON group shows rapid tumor growth, the HMON-SNO or FeGP4@HMON group presents moderate tumor growth inhibition, and the FeGP4@HMON-SNO group exhibits substantial tumor therapeutic efficacy (** *P* < 0.01, *** *P* < 0.001, and **** *P* < 0.0001). Additionally, the survival rate of 4T1 tumor-bearing mice treated with FeGP4@HMON-SNO remained at 100% even after 35 days (Figure 7D), underscoring the remarkable antitumor efficacy of FeGP4@HMON-SNO constructed according to the CIA strategy.

The exceptional efficacy of FeGP4@HMON-SNO in tumor treatment was further substantiated through the histological staining of tumor tissues obtained from various treatments, including hematoxylin and eosin (H&E), ROS, Ki67, TdT-mediated dUTP Nick-End Labeling (TUNEL), and GPX4 (Figure 7E). The FeGP4@HMON-SNO group displays the most pronounced histological damage compared to other treatment formulations, as evidenced by both H&E and TUNEL staining analyses. Compared with other control groups, the Dihydroethidium-stained images of the FeGP4@HMON-SNO group reveal significantly higher ROS fluorescence intensity, while the Ki-67-stained images suggest a more pronounced suppression of proliferation. The red fluorescence of GPX4 is significantly reduced in the FeGP4@HMON-SNO group compared to the other treatments, highlighting the effectiveness of the antitumor intervention attributed to enhanced ferroptosis. Furthermore, the fluorescence intensity of 3-nitrotyrosine (3-NT) in the FeGP4@HMON-SNO group is markedly greater than that observed in the PBS, HMON, and FeGP4@HMON groups, indicating effective NO release within the tumor (Figure S40). Additionally, the collected tumor tissue from 4T1-bearing mice treated with FeGP4@HMON-SNO exhibits significantly elevated levels of MDA compared to the other groups, further suggesting amplified tumor ferroptosis induced by FeGP4@HMON-SNO (Figure S41).

To further elucidate the role of FeGP4@HMON-SNO in tumor ferroptosis, we assessed the mRNA expression levels of the tumor suppressor genes TP53 and KMT2C, as well as the tumor-promoting gene PIK3CA, in collected tumor tissues after various treatments using quantitative PCR (qPCR). As presented in Figure S42, the mRNA expression levels of TP53 and KMT2C in the FeGP4@HMON-SNO group are significantly elevated, while the mRNA expression level of PIK3CA is significantly diminished. These findings provide evidence of its robust antitumor potential through ferroptosis-gas synergistic therapy.

Figure S43 shows that the hemolysis rates of FeGP4@HMON-SNO at varying concentrations remain below 5.0 %, similar to that of saline. The representative H&E-stained images of major organs (heart, liver, spleen, lung, and kidney) from 4T1 tumor-bearing mice after various administrations (Figure S44) show no notable pathological irregularities or signs of inflammation for the major organs of all groups. These results indicate the dependable biosafety of FeGP4@HMON-SNO constructed using our proposed CIA strategy, positioning it as a promising candidate formulation for ferroptosis-gas synergistic tumor therapy.

Although the H&E staining results of various normal tissues and organs indicate that our nanomedicine does not demonstrate acute toxicity, long-term toxicity assessments in mice require further validation. FeGP4@HMON-SNO, as a therapeutic agent, may potentially exert toxic effects on normal cells, particularly at high dosages, which could result in cellular damage or toxic reactions. Additionally, the introduction of exogenous nanomaterials like FeGP4@HMON-SNO may trigger immune responses, leading to inflammatory reactions or adverse effects on the immune system. Lastly, the biological distribution and long-term accumulation of these nanomaterials could have prolonged effects on the organism, highlighting the need for detailed investigations into their biodistribution and metabolism to thoroughly assess their safety profile. Given the potential implications for future applications, we plan to further investigate the concrete metabolic pathways, stability, and long-term toxicity of nanoparticles *in vivo* in our upcoming studies.

In contrast to previously reported NO-mediated ferroptosis enhancement systems^25^, ^27^, ^44^, our approach employs controlled aggregation of FeGP within HMON-SNO, ensuring targeted delivery of therapeutic agents to tumor sites while minimizing systemic side effects. The concurrent release of NO and initiation of the Fenton reaction generates a synergistic effect that amplifies the therapeutic impact on tumor cells. Furthermore, the release of high-contrast MRI agents (GP) in response to the TME enables real-time monitoring of drug delivery and therapeutic response, thereby facilitating precise treatment planning and adjustments.

## 4. Conclusions

In summary, the GP was synthesized as a powerful *T*_1_-weight MRI CA due to its excellent *r*_1_ value (56.2 ± 1.7 mM^-1^ s^-1^, 3.0 T). To realize the MRI visualization of *in vivo* drug delivery, ferroptosis agonists (*i.e.*, Fe^2+^, LAP, BOR, or SOR) were tried to be loaded onto GP. It is observed that the aggregation of FeGP, LAPGP, BQRGP, and SORGP is uncontrollable, which represents a common challenge of drug loading and delivery using MRI CAs.

To overcome this problem, in this study, a strategy of CIA is proposed to restrict the aggregation of MRI CAs at high drug loading conditions. The GP was used to load ferroptosis agonists in the hollow core of HMON-SNO. The resulting FeGP4@HMON-SNO, LAPGP4@HMON-SNO, BQRGP4@HMON-SNO, and SORGP4@HMON-SNO without precipitation exhibit much higher DLC (*i.e.*, 25.6%, 20.6%, 18.7%, and 17.4%) than FeGP4, LAPGP4, BQRGP4, and SORGP4 with serious precipitation (*i.e.*, 7.1%, 13.3%, 9.6%, and 7.8%). The large aggregates within the hollow core of HMON-SNO exceed the mesopore size of HMON-SNO, which effectively impedes the premature release of various ferroptosis agonists. These results collectively demonstrate the feasibility and universality of the proposed CIA strategy.

The synthesized FeGP4@HMON-SNO exhibits tumor accumulation because of its optimal hydrated particle size of 118.7 nm and the EPR effect, which facilitates passive targeting. Upon accumulation in the TME, the presence of GSH triggers the degradation of HMON-SNO, leading to the specific release of Fe^2+^ and NO from FeGP4@HMON-SNO. The released Fe^2+^ initiates a potent Fenton reaction, generating •OH that promotes ferroptosis. Simultaneously, the elevated levels of NO induce mitochondrial dysfunction and inhibit cellular respiration, resulting in a reversal of tumor hypoxia and a downregulation of HIF-1α expression, which is typically upregulated due to oxygen conservation associated with respiratory depression. This disruption reduces the accumulation of LDs, which serve as storage sites for free fatty acids and promotes the release of free PUFAs. The increased release of PUFAs enhances LPO, thereby elevating the incidence of ferroptosis in tumor cells. Furthermore, the degradation of HMON and the production of NO occur concurrently with the depletion of GSH. This depletion compromises the antioxidant defense system, further facilitating ferroptosis in tumor cells. Consequently, the exceptional antitumor activity observed both *in vitro* and *in vivo* can be attributed to the potent synergistic effects of ferroptosis and NO gas therapy provided by FeGP4@HMON-SNO. It is noteworthy that FeGP4@HMON-SNO can undergo metabolic processes followed by excretion via the liver, which may potentially reduce the risk of toxicity associated with long-term retention in the body. Moreover, the released GP was used for tumor high contrast *T*_1_-weighted MRI due to its high *r*_1_ value, enabling real-time monitoring for the *in vivo* delivery of FeGP@HMON-SNO. Therefore, our study presents a comprehensive approach to cancer therapy that integrates MRI-guided drug delivery with the therapeutic potential of ferroptosis-gas synergistic therapy, offering a promising strategy for enhancing both efficacy and safety in cancer treatment.

### Author contributions

Jiaoyang Zhu: Conceptualization, Data curation, Formal analysis, Investigation, Methodology, Validation, Visualization, Writing - original draft. Ling Huang: Conceptualization, Formal analysis, Investigation, Methodology, Visualization, Validation, Writing - original draft. Jing Yang: Formal analysis, Investigation, Methodology, Validation, Visualization. Zongheng Li: Formal analysis, Investigation, Methodology, Validation. Lihe Wu: Formal analysis, Investigation, Methodology. Wei Xiong: Formal analysis, Resource, Methodology. Jie Feng: Formal analysis, Resource, Methodology. Chenggong Yan: Formal analysis, Resource, Methodology, Validation. Chaomin Chen: Conceptualization, Funding acquisition, Methodology, Project administration, Resources, Supervision, Writing - review & editing. Yan Li: Investigation, Project administration, Resources, Supervision, Validation, Visualization, Writing - review & editing. Zheyu Shen: Conceptualization, Formal analysis, Funding acquisition, Investigation, Methodology, Project administration, Resources, Supervision, Validation, Visualization, Writing - review & editing.

## Supplementary Material

Supplementary experimental section, figures and tables.

## References

[1] Cooley CZ, McDaniel PC, Stockmann JP, Srinivas SA, Cauley SF, Sliwiak M (2021). A portable scanner for magnetic resonance imaging of the brain. Nat Biomed Eng.

[2] Yang J, Feng J, Yang SG, Xu YK, Shen ZY (2023). Exceedingly small magnetic iron oide nnoparticles for T_1_-wighted mgnetic rsonance imaging and imaging-guided therapy of tumors. Small.

[3] Guo S, Li ZH, Feng J, Xiong W, ang J, Lu XY (2022). Cycloacceleration of ferroptosis and calcicoptosis for magnetic resonance imaging-guided colorectal cancer therapy. Nano Today.

[4] Zhou ZJ, Yang LJ, Gao JH, Chen XY (2019). Structure-relaxivity relationships of magnetic nanoparticles for magnetic resonance imaging. Adv Mater.

[5] Slobozhanyuk AP, Poddubny AN, Raaijmakers AJ, van den Berg CA, Kozachenko AV, Dubrovina IA (2016). Enhancement of magnetic resonance imaging with metasurfaces. Adv Mater.

[6] Zhang H, Guo YK, Jiao J, Qiu Y, Miao YQ, He Y (2023). A hepatocyte-targeting nanoparticle for enhanced hepatobiliary magnetic resonance imaging. Nat Biomed Eng.

[7] Lazovic J, Goering E, Wild AM, SchutzendUbe P, Shiva A, Loffler J (2024). Nanodiamond-enhanced magnetic resonance imaging. Adv Mater.

[8] Laha SS, Thorat ND, Singh G, Sathish CI, Yi J, Dixit A (2022). Rare-earth doped iron oxide nanostructures for cancer theranostics: magnetic hyperthermia and magnetic resonance imaging. Small.

[9] Wei ZN, Jiang ZQ, Pan CS, Xia JB, Xu KW, Xue T (2020). Ten-gram-scale facile synthesis of organogadolinium complex nanoparticles for tumor diagnosis. Small.

[10] Chen HM, Qiu YW, Ding DD, Lin HR, Sun WJ, Wang GD (2018). Gadolinium-encapsulated graphene carbon nanotheranostics for imaging-guided photodynamic therapy. Adv Mater.

[11] Lu YD, Liang ZY, Feng J, Huang L, Guo S, Yi PW (2022). Facile synthesis of weakly ferromagnetic organogadolinium macrochelates-based T_1_-weighted magnetic resonance imaging contrast agents. Adv Sci.

[12] Guo WH, Ren YX, Chen ZA, Shen GD, Lu YD, Zhou HM (2023). Targeted magnetic resonance imaging/near-infrared dual-modal imaging and ferroptosis/starvation therapy of gastric cancer with peritoneal metastasis. Adv Funct Mater.

[13] Shen ZY, Wu H, Yang SG, Ma XH, Li ZH, Tan MQ (2015). A novel Trojan-horse targeting strategy to reduce the non-specific uptake of nanocarriers by non-cancerous cells. Biomaterials.

[14] Mendez-Lucas A, Lin W, Driscoll PC, Legrave N, Novellasdemunt L, Xie CC (2020). Identifying strategies to target the metabolic flexibility of tumours. Nat Metab.

[15] Jin JK, Yuan PC, Yu W, Lin JT, Xu AK, Xu XD (2020). Mitochondria-targeting polymer micelle of dichloroacetate induced pyroptosis to enhance osteosarcoma immunotherapy. ACS Nano.

[16] Chang MY, Wang M, Liu YH, Liu M, Kheraif AAA, Ma P (2023). Dendritic plasmonic CuPt alloys for closed-loop multimode cancer therapy with remarkably enhanced efficacy. Small.

[17] Fan QD, Xiong W, Zhou HM, Yang J, Feng J, Li ZH (2023). An AND logic gate for magnetic-resonance-imaging-guided ferroptosis therapy of tumors. Adv Mater.

[18] Lu XY, Zhou HM, Liang ZY, Feng J, Lu YD, Huang L (2022). Biodegradable and biocompatible exceedingly small magnetic iron oxide nanoparticles for T_1_-weighted magnetic resonance imaging of tumors. J Nanobiotechnology.

[19] Zhao ZH, Li MY, Zeng J, Huo LL, Liu K, Wei RX (2022). Recent advances in engineering iron oxide nanoparticles for effective magnetic resonance imaging. Bioact Mater.

[20] Zheng JS, Conrad M (2020). The metabolic underpinnings of ferroptosis. Cell Metab.

[21] Chen FQ, Kang R, Tang DL, Liu J (2024). Ferroptosis: principles and significance in health and disease. J Hematol Oncol.

[22] Shen ZY, Song JB, Yung BC, Zhou ZJ, Wu AG, Chen XY (2018). Emerging strategies of cancer therapy based on ferroptosis. Adv Mater.

[23] Lei G, Mao C, Horbath AD, Yan YL, Cai SR, Yao J (2024). BRCA1-mediated dual regulation of ferroptosis exposes a vulnerability to GPX4 and parp co-inhibition in brca1-deficient cancers. Cancer Discovery.

[24] He M, Song YY, Xu W, Zhang XL, Dong CM (2023). Four birds with one stone: a multifunctional polypeptide nanocomposite to unify ferroptosis, nitric oxide, and photothermia for amplifying antitumor immunity. Adv Funct Mater.

[25] Jia L, Y Wang YZ, Hu TT, Yang CY, Lin HM, Zhang F (2023). Boosting the tumor photothermal therapy with hollow CoSnSx-based injectable hydrogel via the sonodynamic and dual-gas therapy. Chem Eng J.

[26] Yu WJ, Jia F, Fu JZ, Chen YH, Huang Y, Jin Q (2023). Enhanced transcutaneous chemodynamic therapy for melanoma treatment through cascaded Fenton-like reactions and nitric oxide delivery. ACS Nano.

[27] Zhou XF, Meng ZQ, She JL, Zhang YJ, Yi X, Zhou HL (2020). Near-infrared light-responsive nitric oxide delivery platform for enhanced radioimmunotherapy. Nanomicro Lett.

[28] Huang L, Lu YD, Guo S, Yang J, Liang ZY, Zhang QQ (2023). A strategy of limited-space controlled aggregation for generic enhancement of drug loading capability. Adv Funct Mater.

[29] Theivendran S, Gu ZY, Tang J, Yang YN, Song H, Yang Y (2022). Nanostructured organosilica nitric oxide donors intrinsically regulate macrophage polarization with antitumor effect. ACS Nano.

[30] Malone-Povolny MJ, Schoenfisch MH (2019). Extended nitric oxide-releasing polyurethanes via s-nitrosothiol-modified mesoporous silica nanoparticles. ACS Appl Mater Interfaces.

[31] Shen ZY, Fan WP, Yang Z, Liu YJ, Bregadze VI, Mandal SK, Yung BC (2019). Exceedingly small gadolinium oxide nanoparticles with remarkable relaxivities for magnetic resonance imaging of tumors. Small.

[32] Li ZM, Yu YK, Zeng WF, Ding F, Zhang D, Cheng W (2022). Mussel-inspired ligand clicking and ion coordination on 2d black phosphorus for cancer multimodal imaging and therapy. Small.

[33] Schildknecht S, von Kriegsheim A, Vujacic-Mirski K, Di Lisa F, Ullrich V (2022). Recovery of reduced thiol groups by superoxide-mediated denitrosation of nitrosothiols. Redox Biol.

[34] Huang DP, Huang HQ, Li ML, Fan JL, Sun W, Du JJ (2022). A tumor-specific platform of peroxynitrite triggering ferroptosis of cancer cells. Adv Funct Mater.

[35] Yang J, Xiong W, Huang L, Li ZH, Fan QD, Hu F (2024). A mesoporous superparamagnetic iron oxide nanoparticle as a generic drug delivery system for tumor ferroptosis therapy. J Nanobiotechnology.

[36] Liu JN, Liu Y, Bu WB, Bu J, Sun Y, Du J (2014). Ultrasensitive nanosensors based on upconversion nanoparticles for selective hypoxia imaging in vivo upon near-infrared excitation. J Am Chem Soc.

[37] P Murphy M (2013). Mitochondrial dysfunction indirectly elevates ros production by the endoplasmic reticulum. Cell Metab.

[38] Li Y, Fan WZ, Gu X, Liu SP, He TT, Gou SQ (2024). Biodegradable ferric phosphate nanocarriers with tumor-specific activation and glutathione depletion for tumor self-enhanced ferroptosis and chemotherapy. Adv Funct Mater.

[39] Gan HB, Huang X, Luo X, Li JL, Mo BH, Cheng LZ (2023). A mitochondria-targeted ferroptosis inducer activated by glutathione-responsive imaging and depletion for triple negative breast cancer theranostics. Adv Healthc Mater.

[40] Del Gobbo LC, Imamura F, Aslibekyan S, Marklund M, Virtanen JK, Wennberg M (2016). Fatty acids outcomes res, ω-3 polyunsaturated fatty acid biomarkers and coronary heart disease pooling project of 19 cohort studies. JAMA INTERN MED.

[41] Pavuluri K, Yang E, Ayyappan V, Sonkar K, Tan Z, Tressler CM (2022). Unlabeled aspirin as an activatable theranostic MRI agent for breast cancer. Theranostics.

[42] Navarria P, Pessina F, Bellu L, Politi S, Savini G, Clerici E (2023). A differentitate treatment effects from disease progression in treated brain tumor: promising results using delayed contrast MRI. Neuro-Oncology.

[43] Leu K, Enzmann D, Woodworth D, Teixeira S, Lai A, Nghiemphu P (2014). Ni-54hyprervascular volume estimated by comparasion to a large-scale cerebral blood volume (CBV) radiographic atlas predicts survival in recurrent blioblastoma treated sith bevacizumab. Neuro-Oncology.

[44] Li WT, Liu SK, Ding H, Zhao RX, Zang PY, Li SY (2024). Three-step depletion strategy of glutathione: tunable metal-organic-framework-engineered nanozymes for driving oxidative/nitrative stress to maximize ferroptosis therapy. Nano Lett.

