# Supplementary material for: A CIA strategy with eminent drug-loading capacities for tumor ferroptosis-gas synergistic therapy
Source: Theranostics. 2024 Oct 28;14(19):7349–69. doi: 10.7150/thno.99295 (PMC11626936; doi:10.7150/thno.99295)
Supplement: Supplementary file 1 — Supplementary experimental section, figures and tables. [file thnov14p7349s1.pdf]

Supporting Information

Jiaoyang Zhu[1], Lin Huang[1,*], Jing Yang[1], Zongheng Li[1], Lihe Wu[1], Wei Xiong[2], Jie Feng[2], Chenggong Yan[2], Chaomin Chen[3,*], Yan Li[3,*], Zheyu Shen[1,*]

[1] *Biomaterials Research Center, School of Biomedical Engineering, Southern Medical University, 1023 Shatai South Road, Baiyun, Guangzhou, Guangdong 510515, China.*

[2] *Medical Imaging Center, Nanfang Hospital, Southern Medical University, 1023 Shatai South Road, Baiyun, Guangzhou, Guangdong 510515, China.*

[3] *Institute of Medical Instruments, School of Biomedical Engineering, Southern Medical University, 1023 Shatai South Road, Baiyun, Guangzhou, Guangdong 510515, China.*

Corresponding Authors

[*]E-mail: sz@smu.edu.cn (Z. Shen), liyan2020@smu.edu.cn (Y. Li), huangem@smu.edu.cn (L. Huang)

Experimental Section

Materials and Reagents: Cetyltrimethylammonium Chloride (CTAC, ≥ 99.9%), Triethanolamine (TEA, ≥ 99.9%), tetraethyl orthosilicate (TEOS, ≥ 97%), Bis-[γ-(triethoxysilylpropyl)] tetrasulfide (BTES, ≥ 97%), absolute ethyl alcohol (C_2H_5OH, ≥ 99%), ammonium hydroxide ($NH_3 \cdot H_2O$, 25-28 %), iron (II) chloride tetrahydrate ($FeCl_2$ $4H_2O$ 99.9%), β-Lapachone, sodium poly (acrylic acid) (PAANa, M_w = 5100) and gadolinium (III) nitrate hexahydrate (Gd $(NO_3)_3 \cdot 6H_2O$, 99.9 %) were purchased from Macklin Reagent (Shanghai, China). Sorafenib (99%) was purchased from KKL Med Inc. Brequinar was purchased from MedChemExpress. 4',6-Diamidino-2-phenylindole (DAPI), lysosomes probe, and calcein acetoxymethyl ester (Calcein-AM) and propidium iodide (PI) were purchased from Beyotime Biotechnology Co., Ltd. (Shanghai, China). Diaminofluorescein-FM diacetate (DAF-FM DA) was purchased from beyotime. The fluorescence $ONOO^-$ assay kit (O71) was purchased from Bestbio, Shanghai, China. All chemical reagents were analytical grade.

Synthesis of Gd-PAA Macrochelate (GP): 40 mL of PAANa (M_w = 5100) solution (4.0 mg/mL) was first purged with nitrogen (≥ 50 min) to eliminate oxygen. The polymer solution was then heated to reflux (100 °C) and 0.8 mL of $Gd(NO_3)_3$ (125 mM) solution was poured into the PAANa solution. After 90 min of reaction under magnetic stirring at 100 °C, the GP was obtained. Finally, the GP solution was cooled down to room temperature and then purified by membrane dialysis (M_w cut-off 6-8 kDa) against Milli-Q water for 3.0 days with water change twice a day. The purified GP was concentrated by rotary evaporation, and then the Gd concentration of the concentrated GP solution was measured by Inductively Coupled Plasma Optical Emission Spectrometry (ICP-OES, iCAP PRO, Thermo Fisher Scientific, US).

Synthesis of HMON: For the synthesis of hollow mesoporous organosilica nanoparticles (HMON), a facile “chemical homology” strategy was used. Briefly, 2.0 g of CTAC and 1.0 mL of TEA aqueous solution (10 wt %) were premixed and kept at 80 °C for 1.0 h under stirring. After that, 1.0 mL of TEOS was dropwise added into the mixture. Mesoporous silica nanoparticles (MSNs) were formed after 1.0 h of hydrolysis/condensation reaction. Afterward, a mixture of silica precursors with 0.5 of BTES to TEOS ratio ($M_{\mathrm{BTES}}/M_{\mathrm{TEOS}}$) was added, and the reaction was continued for another 4.0 h. The resulting core/shell structured mesoporous SiO_2/organosilica nanoparticles (MSN@MON) were obtained by centrifugation at 20000 × g for 15 min, and subsequently washed three times with deionized water and ethanol. To eliminate the CTAC, the acquired MSN@MONs were dispersed in a methanol/sodium chloride mixture, subjected to magnetic stirring for 12 h, and then collected by centrifugation at 25000 × g for 15 min. To etch the MSNs core, the MSN@MONs were redispersed in 100 mL aqueous solution containing 2.0 mL of $NH_3{\cdot}H_2O$ (25-28 %). After 3.0 h of reaction at 95 °C, the final HMON products were collected after centrifugation (20000 × g, 20 min) and washed using Milli-Q water for three times.

Zeta Potential Measurements: 0.50 mL of HMON, HMON-SNO, or FeGP4@HMON-SNO (2.0 mg/mL in pure water) was mixed with 4.0 mL of PBS solution, and the resulting dispersions were maintained at 37 °C under magnetic stirring for 30 min. Then, the above dispersions were diluted for Zeta potential measurements by an instrument of dynamic light scattering (DLS, Nano-Brook 90PlusZata, Brookhaven).

Detection of In Vitro Hydroxyl Radicals (·OH) Production: 1.0 mL of PBS containing 2.0 mM of H_2O_2 was mixed with 1.0 mL of FeGP4@HMON-SNO (C_{Fe}=0.10-0.80 mM) that was pre-incubated with 10 mM of glutathione (GSH) overnight, and resuspended in PBS after

removal of GSH by centrifugation. The mixtures were stirred at room temperature for 0, 12, 24, and 48 h. After that, the supernatants were collected by centrifugation at 20000 × g for 10 min, followed by the addition of MB or TMB solution (0.10 mL, 2.0 mM). After 30 min of incubation, the solutions were measured by UV-Vis spectrophotometer (Evolution 300, Thermo Fisher) at the wavelength of 665 nm (MB) and 652 (TMB) nm to determine the generation of ·OH.

Electron Spin Resonance (ESR) Measurements: To measure ·OH generation *via* ESR, 100 μL of FeGP4@HMON-SNO (5.0 mg/mL), either untreated or pre-treated overnight with 1.0 mL of GSH (10 mM) and subsequently centrifuged to remove the GSH before being resuspended in PBS, was treated with 2.0 mL of PBS buffer containing H_2O_2 (2.0 mM) at 37 ^{o}C for 4.0 h. After that, 100 μL of 5,5-dimethyl-1-pyrroline-N-oxide (DMPO) was added to the above solution, and the ESR signal was recorded by ESR spectrometer (EMXPlus 10/12, Bruker).

Detection of In Vitro NO Production: 1.0 mL of FeGP4@HMON-SNO dispersion (0.25-2.0 mg/mL) was mixed with 1.0 mL of GSH (20 mM) at room temperature under magnetic stirring. After incubation at 37 ^{o}C for 0, 12, 24, and 48 h, the supernatants were collected by centrifugation at 20000 × g for 10 min, followed by the addition of DAF-FM DA solution (0.10 mL, 2.0 mM). After a further 30 min of incubation at 37 ^{o}C, the solutions were analyzed using a UV-Vis spectrophotomheter (Evolution 300, Thermo Fisher) at a wavelength of 524 nm to determine the generation of NO.

Detection of GSH Consumption: To evaluate GSH depletion capability, 400 μL of FeGP4@HMON-SNO (0.25-2.0 mg/mL) was dispersed in GSH solution (5.0 mL, 1.0 mM),

and the mixed solutions were maintained at 37 °C under magnetic stirring for 0, 12, 24, or 48 h. Subsequently, the above mixtures were centrifuged at 20,000 × g for 10 min. The resulting supernatants were then mixed with DTNB (50 μL, 10 mg/mL), and the obtained mixtures were incubated for 30 min at 37 °C. After that, the solutions were measured by a UV-Vis spectrophotometer (Evolution 300, Thermo Fisher) at the wavelength of 412 nm to determine the capability of GSH consumption.

Evaluation of Biodegradability Behavior: FeGP4@HMON-SNO (50 μL, 2.0 mg/mL) was incubated with 1.0 mL of PBS containing 10 mM of GSH at 37 °C under shaking. After incubation for 0, 1.0, 3.0, 5.0, and 7.0 days, 10 μL of the solutions were taken out for measurements on Transmission Electron Microscope (TEM, JEOL, JEM-1400).

Study of Stimulus-Triggered Drug Release: FeGP4@HMON-SNO (1.0 mL, 2.0 mg/mL) was sealed into a dialysis bag (cut off Mw: 12 kDa), and then placed in 10 mL of PBS without or with 10 mM of GSH. The dialysis was implemented at 37 ºC in an incubator with gently shaking at 100 rpm. At predetermined time points of 0.25, 0.50, 1.0, 2.0, 4.0, 8.0, 12, 24, 48, or 72 h, 0.50 mL of the solution was taken out, and 0.50 mL of fresh PBS was supplemented. The released Fe or Gd chelates were quantified by ICP-OES (iCAP PRO, Thermo Fisher Scientific, US). These procedures were repeated three times for each group.

Magnetic Resonance Imaging (MRI) Performance In Vitro: The MRI measurements were performed with a clinical MRI scanner system (3.0 T, Philips, Ingenia, NL). The FeGP4@HMON-SNO dispersion treated with 10 mM of GSH for varying durations was diluted to achieve solutions containing different Gd concentrations (0-200 μM). Subsequently, the diluted dispersions were added into 1.5 mL of centrifuge tubes for MRI measurements.

The relaxivity value of r_1 or r_2 was obtained from the slope of the linear curve of relaxation rate ($1/T_1$ or $1/T_2$, s^{-1}) versus Gd concentration (mM). Meanwhile, MR images were analyzed by measuring signal intensity using the software Image J. The signal-to-noise ratios (SNR) and ΔSNR values (*i.e.*, signal enhancement) were calculated according to the following equations (1) and (2), respectively.

$$SNR = SI_{mean}/SD_{noise} \quad (1)$$

$$\Delta SNR = (SNR_{sample} - SNR_{control})/SNR_{control} \times 100\ \% \quad (2)$$

Cell Culture: 4T1 cells (mouse breast cancer cell line) were cultured in the complete DMEM culture medium supplemented containing 10 % of FBS, 100 U/ml of penicillin G sodium, and 100 mg/mL of streptomycin sulfate. The cells were incubated at 37 ºC in a humidified atmosphere containing 5.0 % of CO_2.

Cellular Uptake: Cellular uptake of FeGP4@HMON-SNO was evaluated *via* laser scanning confocal microscopy (LSCM, Nikon, ECLIPSE Ti2), flow cytometry (BD, FACSAria III, USA), and a clinical MRI scanner (3.0 T, Philips, Ingenia, NL).

By LSCM: 4T1, MCF7 and HGC27 cells (2.0×10^4 cells) were cultured in confocal dishes for 24 h. After that, the growth media were replaced with 0.50 mL of fresh ones containing R6G-labelled FeGP4@HMON-SNO ($C_{Nanoparticle}$ = 100 μg/mL). After 1.0, 2.0, 4.0 h of incubation, the treated cells were washed with PBS twice, fixed with 4.0 % of paraformaldehyde for 30 min, permeabilized with 0.10 % of Triton X-100 for 5.0 min, blocked with 1.0 % of BSA for 30 min, and then stained with a mixture of 4',6-diamidino-2-phenylindole (DAPI, Beyotime Biotechnology) and Phalloidin-FITC solution for 30 min at room temperature. Finally, the cells were observed by the LSCM.

By Flow Cytometry: 4T1 cells (2.0×10^5 cells per well) were cultured in a 6-well plate for

24 h and treated with R6G-labelled FeGP4@HMON-SNO ($C_{Nanoparticle}$ = 100 μg/mL) for 4.0 h. The treated cells were washed with cold PBS twice, trypsinized, and harvested by centrifugation at 1,000 × g for 3.0 min. The cells were then resuspended in 200 μL of PBS and then detected by the flow cytometry.

By MRI: 4T1 cells (2.0 × 10^5 cells per well) were seeded in 6-well plates, cultured for 24 h, and then treated with FeGP4@HMON-SNO ($C_{Nanoparticle}$ = 100 μg/mL) for different time intervals (1.0, 2.0, or 4.0 h) at 37 °C. Next, the cells were harvested into 1.5 mL of centrifuge tubes by trypsinization and centrifugation procedures (1,000 × g, 3.0 min), followed by the addition of 0.20 mL of agarose solution (0.80 wt%) to fix the cells. T_1-weighted MR images of cell samples were acquired using the 3.0 T MRI scanner under the following parameters: TE = 8.2 ms, TR = 500 ms.

Lysosome Escape Assay: 0.50 mL of 4T1 cells (4.0 × 10^4 cells/mL) in complete growth medium were seeded into confocal dishes and allowed to adhere at 37 °C for 24 h. Subsequently, the growth medium was replaced with a fresh one (without FBS) containing R6G-labelled FeGP4@HMON-SNO ($C_{Nanoparticle}$ = 100 μg/mL). After treatment for 1.0-4.0 h, the cells were fixed by 4.0 % of paraformaldehyde for 20 min, the nuclei were stained using DAPI for 10 min, and the lysosomes were stained with Lyso-Tracker Green reagents (50 nM) for 30 min. Finally, the cells were observed by the LSCM (Nikon, ECLIPSE Ti2).

Evaluation of Intracellular ROS Generation via DCFH-DA Staining Assay: The intracellular generation of ROS was determined by a fluorogenic reagent 2,7-dichlorofluorescein diacetate (DCFH-DA), which could be oxidized to the highly fluorescent dichlorofluorescein (DCF) by ROS.

By LSCM: 0.50 mL of 4T1 cells (4.0 × 10^4 cells/mL) in complete growth medium were

seeded into confocal dishes, and allowed to adhere at 37 ºC for 24 h. After that, the growth medium was replaced with a fresh one (without FBS) containing (I) PBS, (II) HMON-SNO, (III) FeGP4@HMON, or (IV) FeGP4@HMON-SNO ($C_{Nanoparticle}$ = 100 μg/mL). After 4.0 h of incubation at 37 ºC, the culture media were removed, and the cells were washed thrice with PBS. After that, 1.0 mL of fresh culture media (with 2.0% FBS) containing DCFH-DA (20 μM) was added to each dish, and the cells were cultured at 37 °C for 30 min. After washing with PBS for three times, the images of the samples were captured by an LSCM imaging system (Nikon, ECLIPSE Ti2).

By Flow Cytometry: 2.0 mL of 4T1 cells (2.0×10^5 cells/mL) in a complete growth medium were seeded into a 6-well culture plate, and allowed to adhere at 37 ºC for 24 h. After that, the growth medium was replaced using a fresh one (without FBS) containing (I) PBS, (II) HMON-SNO, (III) FeGP4@HMON, or (IV) FeGP4@HMON-SNO ($C_{Nanoparticle}$ = 100 μg/mL). After 4.0 h of incubation at 37 ºC, the culture media were removed, and the cells were washed thrice with PBS. After that, 1.0 mL of fresh culture media (with 2.0% FBS) with 20 μM of DCFH-DA was added to each well, and the cells were cultured at 37 °C for 30 min. The treated cells were washed with cold PBS twice, trypsinized, and harvested by centrifugation at 1,000 × g for 3.0 min. The obtained cells were resuspended in 0.20 mL of PBS and then measured by the flow cytometer (BD, FACSAria III, USA). Data analysis was performed using the flow cytometry analysis software (FlowJo V10).

Evaluation of Intracellular NO Generation via DAF-FM DA Staining Assay: 0.50 mL of 4T1 cells (4.0×10^4 cells/mL) in complete growth medium were seeded into confocal dishes, and allowed to adhere at 37 ºC for 24 h. After that, the growth medium was replaced with a fresh one (without FBS) containing PBS, HMON-SNO, FeGP4@HMON, FeGP4@HMON-SNO, or FeGP4@HMON-SNO plus 0.5 μM RSL3 ($C_{Nanoparticle}$ = 100

μg/mL). After 4.0 h of incubation at 37 ºC, the culture media were removed, and the cells were washed thrice with PBS. After that, 1.0 mL of fresh culture media (with 2.0% FBS) containing 5.0 μM of DAF-FM DA was added to each dish, and the cells were cultured at 37 °C for 30 min. The cells were washed three times with PBS. Subsequently, the images of the samples were captured by the LSCM imaging system (Nikon, ECLIPSE Ti2).

Evaluation of Intracellular $ONOO^-$ Generation via Staining O71 Assay: 0.50 mL of 4T1 cells (4.0×10^4 cells/mL) in complete growth medium were seeded into confocal dishes, and allowed to adhere at 37 ºC for 24 h. After that, the growth medium was replaced with a fresh one (without FBS) containing (I) PBS, (II) HMON-SNO, (III) FeGP4@HMON, or (IV) FeGP4@HMON-SNO ($C_{\mathrm{Nanoparticle}}$ = 100 μg/mL). After 4.0 h of incubation at 37 ºC, the culture media were removed, and the cells were washed thrice with PBS. After that, 1.0 mL of fresh culture media (with 2.0% FBS) containing 5.0 μL of O71 dyes were added to each dish, and the cells were cultured at 37 °C for 20 min. The cells were washed thrice with PBS. After that, the images of the samples were observed on the LSCM imaging system (Nikon, ECLIPSE Ti2).

Assay of Intracellular GSH Level and GPX4 Activity: 4T1 cells (2.0 mL, 2.0×10^5 cells/mL) were seeded into 6-well plates and cultured for 24 h. After that, the cells were treated with (I) PBS, (II) HMON-SNO, (III)FeGP4@HMON, or (IV) FeGP4@HMON-SNO ($C_{\mathrm{Nanoparticle}}$ = 100 μg/mL) for 12 h, respectively. The GSH level and GPX4 activity were respectively detected using a GSH Assay Kit, and a GSH Peroxidase Assay Kit according to the manufacturer's instructions. Moreover, the 4T1 cells (seeded on confocal dishes) after different treatments were stained with Thiol Tracker Violet (10.0 μM) for 30 min, and then observed by the LSCM (Nikon, ECLIPSE Ti2).

Detection of Intracellular O_2 Level: 0.5 mL of 4T1 cells (4.0 × 10^4 cells/mL) were seeded into confocal dishes and incubated for 24 h to adhere. After that, the cells were treated with (I) PBS, (II) HMON-SNO, (III) FeGP4@HMON, or (IV) FeGP4@HMON-SNO ($C_{Nanoparticle}$ = 100 μg/mL) for 12 h. Thereafter, the cells were incubated with $[Ru(DPP)_3]Cl_2$ dye solution (dilution 1:200) for 25 min. The cells were further rinsed three times by PBS and the cell nucleus was stained by Hoechst for 15 min. Finally, the fluorescence images were observed by the LSCM (Nikon, ECLIPSE Ti2).

Detection of Lipid Peroxides (LPO): LPO was typically detected by a BODIPY-C11 fluorescence probe. First, 4T1 cells were seeded in confocal dishes (0.5 mL, 4.0 × 10^4 cells/mL) or 6-well plates (2.0 mL, 2.0 × 10^5 cells/mL) and cultured for 24 h. After that, the growth medium was replaced with a fresh one (without FBS) containing (I) PBS, (II) HMON-SNO, (III) FeGP4@HMON, or (IV) FeGP4@HMON-SNO ($C_{Nanoparticle}$ = 100 μg/mL). After 6.0 h of incubation at 37 ºC, the cells were washed thrice with PBS and incubated with a fresh DMEM containing BODIPY-C11 (5.0 μM) for 20 min, followed by staining by DAPI for 10 min. Finally, the cells in confocal dishes and 6-well plates were washed with PBS, and then respectively subjected to LSCM (Nikon, ECLIPSE Ti2) observation and flow cytometry (BD, FACSAria III, USA) evaluation.

Intracellular Malondialchehyche (MDA) Assay: 4T1 cells were seeded into 6-well plates (2.0 × 10^5 cells per well) and cultured for 24 h. After that, the cells were treated with (I) PBS, (II) HMON-SNO, (III) FeGP4@HMON, or (IV) FeGP4@HMON-SNO ($C_{Nanoparticle}$ = 100 μg/mL) in a normoxic incubator for 12 h, respectively. The intracellular MDA contents were detected by the MDA Assay Kit according to the manufacturer's protocol. The results of all experimental groups were normalized according to the control group.

Lipid Droplets Storage: For detection of intracellular lipid droplets storage, 4T1 cells were cultured in 12-well plates (1.0×10^5 cells per well) for 24 h, and then incubated with 1.0 mL oleic acid (OA, 100 μM) for another 2.0 h. Next, the growth medium was replaced with a fresh one (without FBS) containing (I) PBS, (II) HMON-SNO, (III) FeGP4@HMON, or (IV) FeGP4@HMON-SNO ($C_{\text{Nanoparticle}}$ = 100 μg/mL). After 4.0 h incubation, the cells were fixed with 4.0% paraformaldehyde for 30 min, and stained with Oil Red O for 30 min (3.0 mg/mL, dissolved in lPA/H_2O = 3:2). The lipid droplet storage was directly observed through a microscope (Nikon, LV100NPOL/ Ci-POL). Additionally, Oil Red O in treated 4T1 cells was further dissolved in isopropyl alcohol for the quantitative analysis using the UV-Vis spectrophotometer (Evolution 300, Thermo Fisher).

Detection of DNA Damage: γ-H2AX immunofluorescence analysis was used for the detection of DNA damage. Typically, 0.5 mL of 4T1 cells (4.0×10^4 cells/mL) were seeded into confocal dishes, and incubated for 24 h to adhere. The cells were then treated with (I) PBS, (II) HMON-SNO, (III) FeGP4@HMON, and (IV) FeGP4@HMON-SNO ($C_{\text{Nanoparticle}}$ = 100 μg/mL) for 12 h. The cells were washed with PBS, fixed with 4.0 % paraformaldehyde, permeabilized with 0.1 % Triton X-100, and blocked with 1.0 % BSA in sequence. The obtained cells were incubated with anti-phospho-histone γ-H2AX rabbit monoclonal antibody (dilution 1: 1000) overnight at 4.0 °C, and further stained with FITC-conjugated anti-rabbit antibody for 1.0 h. After washing for several times with PBS, the cell nuclei were stained with DAPI for 15 min. Finally, the fluorescence images were taken by the LSCM (Nikon, ECLIPSE Ti2).

5-Ethynyl-2-deoxyuridine (EdU) Proliferation Assay: 4T1 cells (0.5 mL, 4.0×10^4

cells/mL) were seeded into confocal dishes and cultured for 24 h. The cells were treated with (I) PBS, (II) HMON-SNO, (III) FeGP4@HMON, and (IV) FeGP4@HMON-SNO ($C_{Nanoparticle}$ = 100 μg/mL) for 24 h. Then, the cells were fixed with 4.0% paraformaldehyde for 10 min and permeabilized with 0.1 % Triton X-100 for 10 min at room temperature. Thereafter, the cells were stained with DAPI for 10 min, and a click additive solution containing Azide 488 for 30 min, according to the instructions provided in the BeyoClick™ Edu-488 Proliferation Kit. Finally, the cells were rinsed with PBS and observed by the LSCM (Nikon, ECLIPSE Ti2).

Monitoring the Changes of Mitochondrial Membrane Potential: 4T1 cells (0.5 mL, 4.0 × 10^4 cells/mL) were seeded into confocal dishes and incubated for 24 h to adhere. After that, the cells were treated with (I) PBS, (II) HMON-SNO, (III) FeGP4@HMON, or (IV) FeGP4@HMON-SNO ($C_{Nanoparticle}$ = 100 μg/mL) for 12 h. Afterward, the cells were stained with 10.0 μg/mL of JC-1 dye (JC-1 monomers, Ex = 514 nm, Em = 529 nm; JC-1 aggregates, Ex = 585 nm, Em = 590 nm) for 30 min, and imaged *via* the LSCM (Nikon, ECLIPSE Ti2).

For flow cytometry analysis, 4T1 cells (1.5 × 10^5 cells/well) were seeded into 6-well plates and cultured for 24 h to adhere. The cells were then treated with (I) PBS, (II) HMON-SNO, (III) FeGP4@HMON or (IV) FeGP4@HMON-SNO ($C_{Nanoparticle}$ = 100 μg/mL) for 12 h. Subsequently, the treated cells were washed twice with PBS and collected by centrifugation (2000 × g, 3.0 min), followed by resuspension in 190 μL of cold PBS solution. Next, 10.0 μL of JC-1 dye (10 μg/mL) was added to the suspensions, and the mixture was incubated in the dark for at least 15 min before flow cytometry analysis (BD, FACSAria III, USA).

Observation of Mitochondria Integrity: 4T1 cells (0.5 mL, 4.0 × 10^4 cells/mL) were seeded into confocal dishes and incubated for 24 h to adhere. After that, the cells were treated

with (I) PBS, (II) HMON-SNO, (III) FeGP4@HMON, or (IV) FeGP4@HMON-SNO ($C_{Nanoparticle}$ = 100 μg/mL) for 12 h. Afterward, the cells were stained by Mito-Tracker Red CMXRos (20 nM) for 30 min. Next, the obtained cells were washed with PBS, fixed with 4.0 % paraformaldehyde for 20 min, and stained by DPAI for 10 min. After washing several times with PBS, the fluorescence images of cells were taken by the LSCM (Nikon, ECLIPSE Ti2).

Observation of Cell Membrane Morphology: 4T1 cells (0.5 mL, 4.0 × 10^4 cells/mL) were seeded into confocal dishes and cultured for 24 h. The cells were then treated with (I) PBS, (II) HMON-SNO, (III) FeGP4@HMON, or (IV) FeGP4@HMON-SNO ($C_{Nanoparticle}$ = 100 μg/mL). After 12 h of incubation, the cells were cultured with 3,3′-dioctadecyloxacarbocyanine perchlorate (DIO) solution (10.0 μg/mL) for 30 min. The cells were further rinsed three times by PBS and the nuclei were stained with DAPI for 15 min. Finally, the fluorescence images were observed by the LSCM (Nikon, ECLIPSE Ti2).

For flow cytometry analysis, 4T1 cells (1.5 × 10^5 cells/well) were seeded into 6-well plates and cultured for 24 h to adhere. The cells were then treated with (I) PBS, (II) HMON-SNO, (III) FeGP4@HMON, or (IV) FeGP4@HMON-SNO ($C_{Nanoparticle}$ = 100 μg/mL) for 12 h. Next, the treated cells were washed twice with PBS and harvested by centrifugation (2000 × g, 3.0 min), followed by suspension into 190 μL of cold PBS solution. Subsequently, 10.0 μL of DIO dye (100 μg/mL) was added to the above suspensions, which were incubated in the dark for over 15 min before flow cytometry analysis (BD, FACSAria III, USA).

Live/Dead Staining Assay: 4T1 cells (0.5 mL, 4.0 × 10^4 cells/mL) were seeded into confocal dishes and incubated for 24 h to adhere. The cells were then treated with (I) PBS, (II) HMON-SNO, (III) FeGP4@HMON, or (IV) FeGP4@HMON-SNO ($C_{Nanoparticle}$ = 100 μg/mL)

for 12 h. Thereafter, the cells were stained with calcein-AM (5.0 μg/mL) and PI (10.0 μg/mL) for 30 min. The obtained cells were rinsed with PBS, and observed by the LSCM (Nikon, ECLIPSE Ti2).

For flow cytometry analysis, 4T1 cells (1.5 × 10^5 cells/well) were seeded into 6-well plates and cultured for 24 h to adhere. The cells were then treated with (I) PBS, (II) HMON-SNO, (III) FeGP4@HMON, or (IV) FeGP4@HMON-SNO ($C_{\text{Nanoparticle}}$ = 100 μg/mL) for 12 h. Next, the treated cells were washed twice with PBS, and harvested by centrifugation (2000 × g, 3.0 min), followed by suspension into 185 μL of cold PBS solution. 5.0 μL of calcein-AM and 10 μL of PI were added to the above suspensions, which were then incubated in the dark for over 15 min before flow cytometry analysis (BD, FACSAria III, USA).

Cytotoxicity Assay: 4T1, MCF-7, or HGC-27 cells (5.0 × 10^3 cells/well) were seeded into 96-well plates and incubated overnight at 37 °C. After washing with PBS, the cells were treated with HMON-SNO, FeGP4@HMON, or FeGP4@HOMON-SNO at varying concentrations ($C_{\text{Nanoparticle}}$ = 0-100 μg/mL) for 24 h. Then, 10 μL of methyl thiazolyl tetrazolium (MTT, 5.0 mg/mL) was added to each well. After an additional 4.0 h of incubation, the culture media was removed, and then 100 μL of DMSO was added to the cells. The absorbance was recorded at a wavelength of 570 nm using a microplate reader (Biotek Synergy H1).

Quantitative time PCR (Q-PCR): 4T1 cells (5.0 × 103 cells/well) were seeded into 96-well plates and incubated overnight at 37 °C. After washing with PBS, the cells were treated with HMON-SNO, FeGP4@HMON, or FeGP4@HOMON-SNO ($C_{\text{Nanoparticle}}$ = 0-100 μg/mL) for 2.0 h. Then the cells were mixed well with TRIzol (15596026, Thermo) to extract total RNA followed by reverse transcription into cDNA using a kit (CW2569, CWBIO). The cDNA was

amplified with Ultra SYBR Mixture (CW2601, CoWin Biosciences) on Quant Studio 1 (Thermo). The amplification procedure was as follows: 95 C for 30 s and 40 cycles of 95 C for 5 s and 60 C for 15 s. The relative expression of the target gene was calculated by the $2^{-\triangle\triangle Ct}$ method, with GAPDH and U6 as internal references.

Tumor Model: All animal procedures were performed in accordance with the Guidelines for Care and Use of Laboratory Animals of Southern Medical University, and approved by the Animal Ethics Committee of Southern Medical University. The assigned approval/accreditation number is SYXK(YUE)2021-0167.

To establish the xenograft tumor models, female Balb/c mice (five-week-old, 15-20 g) were subcutaneously implanted with 100 μL of 5.0×10^6 4T1 cells in the right back side. The size of tumors was measured every other day with a vernier caliper, and the tumor volumes were calculated as follows: tumor volume (mm^3) = $width^2 \times length / 2$.

In Vivo Pharmacokinetics and Biodistribution of Nanoparticles: For the pharmacokinetic analysis ($n = 3$), 4T1 tumor-bearing mice were injected with 100 μL of FeGP4@HMON-SNO (Fe or Gd dosage is 5.0 mg/kg) *via* the tail-vein. After injection, venous blood in the orbit of mice was collected at different times, and the concentration of Fe or Gd was determined using ICP-OES (iCAP PRO, Thermo Fisher Scientific, US).

To investigate the tumor accumulation, the 4T1 tumor-bearing Balb/c mice ($n = 3$) were administrated with 100 μL of FeGP4@HMON-SNO (Fe dosage is 5.0 mg/kg) intravenously. At 0, 0.5, 2, 4, 8, 12, 18, 24, and 48 h post-injection, the major organs (heart, liver, spleen, lung, kidney) and tumors were collected and digested with concentrated nitric acid. The Fe contents in all samples were measured by ICP-OES (iCAP PRO, Thermo Fisher Scientific, US).

In Vivo MRI Examination: *In vivo* T_1-MRI evaluation of FeGP4@HMON-SNO was performed using a 7.0 T MRI scanner (Bruker, PharmaScan 70/16, US). The parameters were shown as: TR/TE =500/8.4 ms, FOV= 50 × 50 mm^2, matrix size =252 × 248, number of averages = 4.0, scan duration = 232 s. 100 μL of contrast agents FeGP4@HMON-SNO (Fe dosage is 5.0 mg/kg) or Gadavist® (Gd dosage is 5.0 mg/kg) were injected into 4T1-bearing mice (n = 3) *via* the tail vein after anesthesia, and the T_1-weighted MR images of 4T1-bearing mice were acquired using the MRI scanner at predetermined time points after injection.

Tumor Therapy Performance: When 4T1 tumors of the mice grew to a volume of ~100 mm^3, the mice were randomly divided into five groups (n = 5), and treated with (I) PBS, (II) HMON, (III) HMON-SNO, (IV) FeGP4@HMON or (V) FeGP4@HMON-SNO *via* tail-vein injection (100 μL, Fe dosage of 5.0 mg/kg, or an equivalent nanoparticle dosage of 13.0 mg/kg). The administration was conducted on the day 0 and 4. The tumor volume and body weight were recorded every other day.

The obtained tumor tissues were dissected, and stained by hematoxylin and eosin (H&E), ROS, GPX4, 3-NT, terminal deoxynucleotidyl transferase (TdT)-mediated deoxyuridine triphosphate (dUTP)-biotin nick-end labeling (TUNEL), or Ki-67 for histological analysis. Furthermore, the MDA level in these collected tumor tissues was detected by the MDA Assay Kit according to the manufacturer's protocol.

Hemolysis Analysis: Hemolysis analysis (n = 3) was performed to examine the hemocompatibility of the FeGP4@HMON-SNO. Typically, 1.5 mL of blood collected from the inner canthus vein plexus of mice was diluted with 3.5 mL of saline, followed by centrifugation and washing. The obtained pure red blood cells (RBCs) were then suspended in

5.0 mL of saline. Thereafter, 100 μL of RBCs suspension was mixed with 900 μL saline (negative control), water (positive control), or various concentrations of FeGP4@HMON-SNO (50, 100, 200, or 400 μg/mL) dispersed in saline. After 2.0 h of incubation at 37 ºC, all the samples were centrifuged at 15,000 × g for 5.0 min. Then, the photos of the samples were taken, and the absorbance of the obtained supernatants was measured at 540 nm using a UV-Vis spectrophotometer (Evolution 300, Thermo Fisher). The hemolysis rate was calculated according to the following formula: hemolysis rate (%) = $[(Ab_{sample}-Ab_{negative\ control})/(Ab_{positive\ control}-Ab_{negative\ control})] \times 100\%$.

Statistical Analysis: A software named SPSS 25.0 (SPSS, Chicago, USA) was used for the statistical analysis. All experiments were performed at least in triplicate, and all experimental data are presented as Mean ± SD. Two groups were compared using Student's t-test, and more than two groups were compared using a one-way analysis of variance (ANOVA) followed by a post hoc Tukey's test. The significance level was fixed as * $P < 0.05$, ** $P < 0.01$, or *** $P < 0.001$.

Table S1. Synthesis conditions and characterization results of FeGP@1-5.

Sample	GP [a] (mL)	$FeCl_2$ $4H_2O$ [b] (mL)	PBS (mL)	Fe Loading Content [c] (%)	Fe Loading Efficiency [d] (%)	Precipitation (overnight)
FeGP1	0.10	0.02	0.88	3.2	28.9	Yes
FeGP2	0.10	0.04	0.86	4.8	21.6	Yes
FeGP3	0.10	0.08	0.82	5.4	12.2	Yes
FeGP4	0.10	0.16	0.74	7.1	7.99	Yes
FeGP5	0.10	0.32	0.58	8.4	4.73	Yes

a) The concentration of feeding GP dispersed in pure water is 7.2 mg/mL.
b) The concentration of feeding Fe^{2+} is 4.0 mg/mL.
c) Calculated from the mass percentage of the loaded Fe^{2+} to the GP.
d) Calculated from the mass percentage of the loaded Fe^{2+} to the feeding Fe^{2+}.

Table S2. Synthesis conditions and characterization results of LAPGP1-4.

Sample	GP [a] (mL)	LAP [b] (mL)	PBS (mL)	LAP Loading Content [c] (%)	LAP Loading Efficiency [d] (%)	Precipitation (overnight)
LAPGP1	0.10	0.02	0.88	3.8	34.2	No
LAPGP2	0.10	0.04	0.86	7.4	33.3	No
LAPGP3	0.10	0.08	0.82	9.9	22.3	Yes
LAPGP4	0.10	0.16	0.74	13.3	15.0	Yes

[a)] The concentration of feeding GP dispersed in pure water is 7.2 mg/mL.
[b)] The concentration of feeding LAP is 4.0 mg/mL.
[c)] Calculated from the mass percentage of the loaded LAP to GP.
[d)] Calculated from the mass percentage of the loaded LAP to the feeding LAP.

Table S3. Synthesis conditions and characterization results of BQRGP1-4.

Sample	GP [a] (mL)	BQR [b] (mL)	PBS (mL)	BQR Loading Content [c] (%)	BQR Loading Efficiency [d] (%)	Precipitation (overnight)
BQRGP1	0.10	0.02	0.88	1.5	13.2	No
BQRGP2	0.10	0.04	0.86	3.8	17.3	No
BQRGP3	0.10	0.08	0.82	7.6	17.1	No
BQRGP4	0.10	0.16	0.74	9.6	10.8	Yes

a) The concentration of feeding GP dispersed in pure water is 7.2 mg/mL.

b) The concentration of feeding BQR is 4.0 mg/mL.

c) Calculated from the mass percentage of the loaded BQR to GP.

d) Calculated from the mass percentage of the loaded BQR to the feeding BQR.

Table S4. Synthesis conditions and characterization results of SORGP1-4.

Sample	GP [a] (mL)	SOR [b] (mL)	PBS (mL)	SOR Loading Content [c] (%)	SOR Loading Efficiency [d] (%)	Precipitation (overnight)
SORGP1	0.10	0.02	0.88	2.8	25.2	No
SORGP2	0.10	0.04	0.86	4.1	18.5	No
SORGP3	0.10	0.08	0.82	7.8	17.6	Yes
SORGP4	0.10	0.16	0.74	10.3	11.6	Yes

a) The concentration of feeding GP dispersed in pure water is 7.2 mg/mL.

b) The concentration of feeding SOR is 4.0 mg/mL.

c) Calculated from the mass percentage of the loaded SOR to GP.

d) Calculated from the mass percentage of the loaded SOR to the feeding SOR.

Table S5. Synthesis conditions and characterization results of FeGP1-5@HMON-SNO.

Sample	GP [a] (mL)	$FeCl_2\ 4H_2O$ [b] (mL)	HMON-SNO (mL)	PBS (mL)	Fe Loading Content [c] (%)	Fe Loading Efficiency [d] (%)	Precipitation (overnight)
FeGP1@HMON-SNO	0.10	0.02	0.58	0.30	10.1	90.6	No
FeGP2@HMON-SNO	0.10	0.04	0.58	0.28	16.3	73.4	No
FeGP3@HMON-SNO	0.10	0.08	0.58	0.24	26.0	58.4	No
FeGP4@HMON-SNO	0.10	0.16	0.58	0.16	38.8	43.6	No
FeGP5@HMON-SNO	0.10	0.32	0.58	0	51.4	28.9	Yes

[a)] The concentration of feeding GP dispersed in pure water is 7.2 mg/mL.
[b)] The concentration of feeding Fe^{2+} is 4.0 mg/mL.
[c)] Calculated from the mass percentage of the loaded Fe^{2+} to GP.
[d)] Calculated from the mass percentage of the loaded Fe^{2+} to the feeding Fe^{2+}.

Table S6. Synthesis conditions and characterization results of LAPGP1-4@HMON-SNO.

Sample	GP [a] (mL)	$FeCl_2$ $4H_2O$ [b] (mL)	HMON-SNO (mL)	PBS (mL)	LAP Loading Content [c] (%)	LAP Loading Efficiency [d] (%)	Precipitation (overnight)
LAPGP1@HMON-SNO	0.10	0.02	0.58	0.30	10.2	91.4	No
LAPGP2@HMON-SNO	0.10	0.04	0.58	0.28	15.0	67.3	No
LAPGP3@HMON-SNO	0.10	0.08	0.58	0.24	23.6	53.2	No
LAPGP4@HMON-SNO	0.10	0.16	0.58	0.16	39.5	44.4	No

a) The concentration of feeding GP dispersed in pure water is 7.2 mg/mL.

b) The concentration of feeding LAP is 4.0 mg/mL.

c) Calculated from the mass percentage of the loaded LAP to GP.

d) Calculated from the mass percentage of the loaded LAP to the feeding LAP.

Table S7. Synthesis conditions and characterization results of BQRGP1-5@HMON-SNO.

Sample	GP [a] (mL)	$FeCl_2$ $4H_2O$ [b] (mL)	HMON-SNO (mL)	PBS (mL)	BQR Loading Content [c] (%)	BQR Loading Efficiency [d] (%)	Precipitation (overnight)
BQRGP1@HMON-SNO	0.10	0.02	0.58	0.30	9.9	89.1	No
BQRGP2@HMON-SNO	0.10	0.04	0.58	0.28	15.7	70.6	No
BQRGP3@HMON-SNO	0.10	0.08	0.58	0.24	25.2	56.7	No
BQRGP4@HMON-SNO	0.10	0.16	0.58	0.16	35.4	39.8	No

a) The concentration of feeding GP dispersed in pure water is 7.2 mg/mL.

b) The concentration of feeding BQR is 4.0 mg/mL.

c) Calculated from the mass percentage of the loaded BQR to GP.

d) Calculated from the mass percentage of the loaded BQR to the feeding BQR.

Table S8. Synthesis conditions and characterization results of SORGP1-5@HMON-SNO.

Sample	GP [a] (mL)	$FeCl_2$ $4H_2O$ [b] (mL)	HMON-SNO (mL)	PBS (mL)	SOR Loading Content [c] (%)	SOR Loading Efficiency [d] (%)	Precipitation (overnight)
SORGP1@HMON-SNO	0.10	0.02	0.58	0.30	10.1	90.6	No
SORGP2@HMON-SNO	0.10	0.04	0.58	0.28	15.2	68.4	No
SORGP3@HMON-SNO	0.10	0.08	0.58	0.24	23.6	53.2	No
SORGP4@HMON-SNO	0.10	0.32	0.58	0.16	36.8	41.4	No

a) The concentration of feeding GP dispersed in pure water is 7.2 mg/mL.

b) The concentration of feeding SOR is 4.0 mg/mL.

c) Calculated from the mass percentage of the loaded SOR to GP.

d) Calculated from the mass percentage of the loaded SOR to the feeding SOR.

Table S9. Scoring results of docking between small molecules and proteins.

ligand	Binding energy (kcal/mol)	Predicted Ki
NO	-5.755	6.01×10^{-5}M

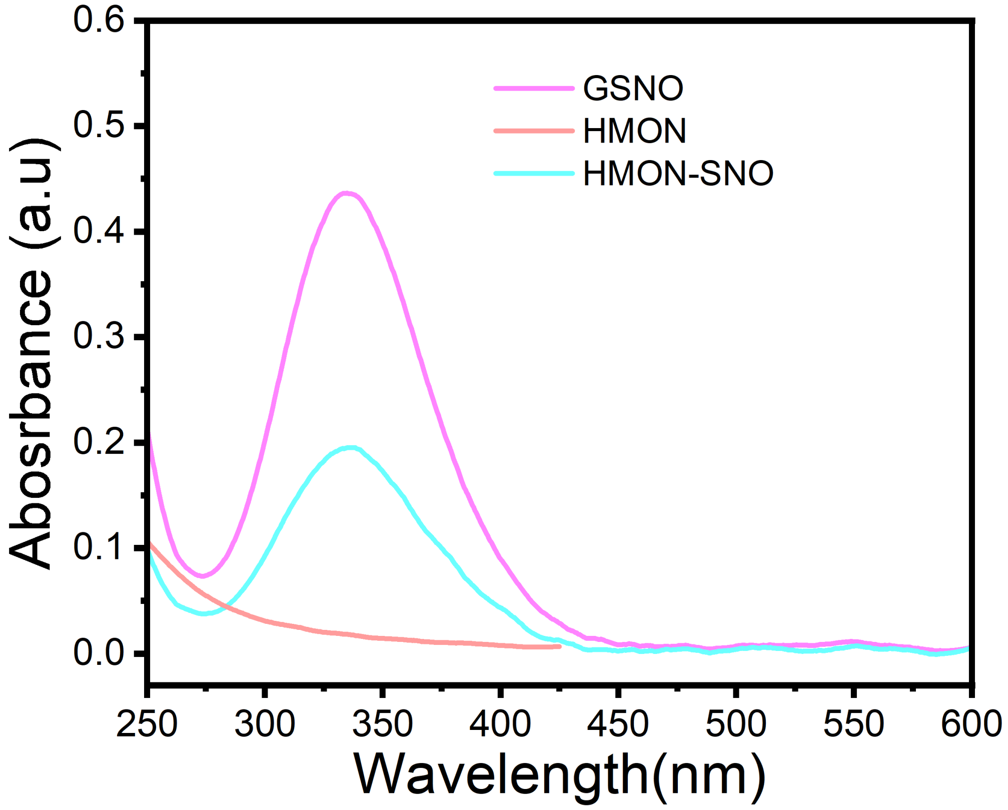

Figure S1. UV-Vis spectra of GSNO, HMON, HMON-SNO samples.

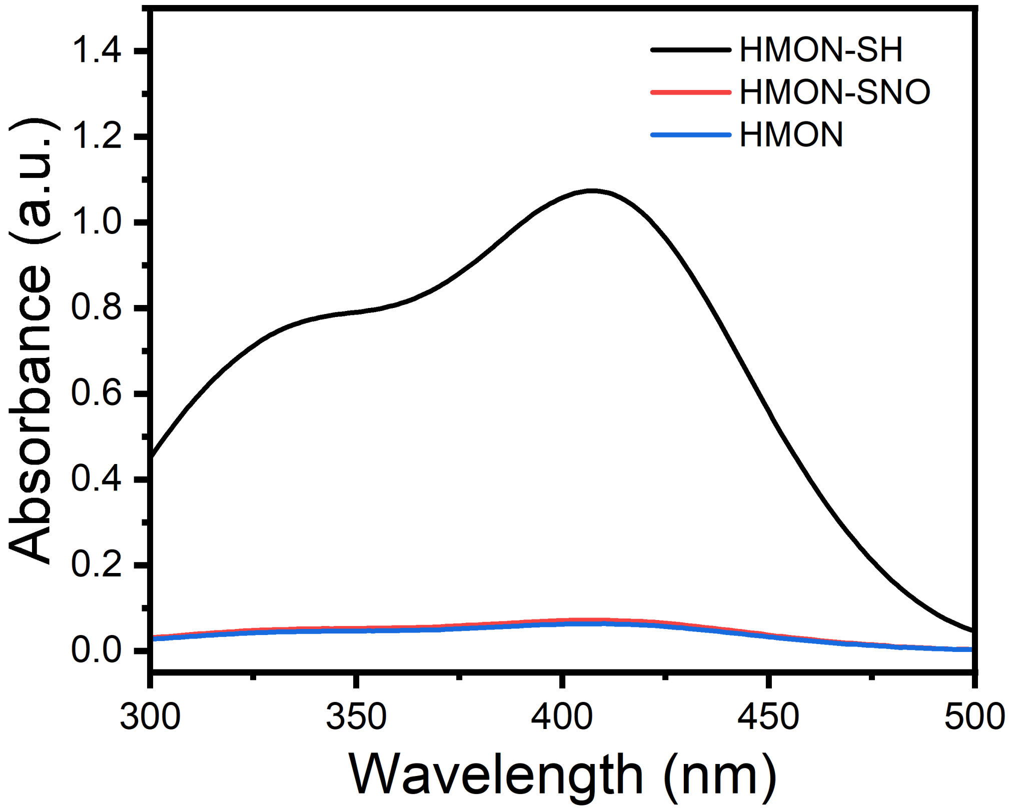

Figure S2. UV-Vis spectra of HMON, HMON-SNO, and HMON-SH after incubation with DTNB solution.

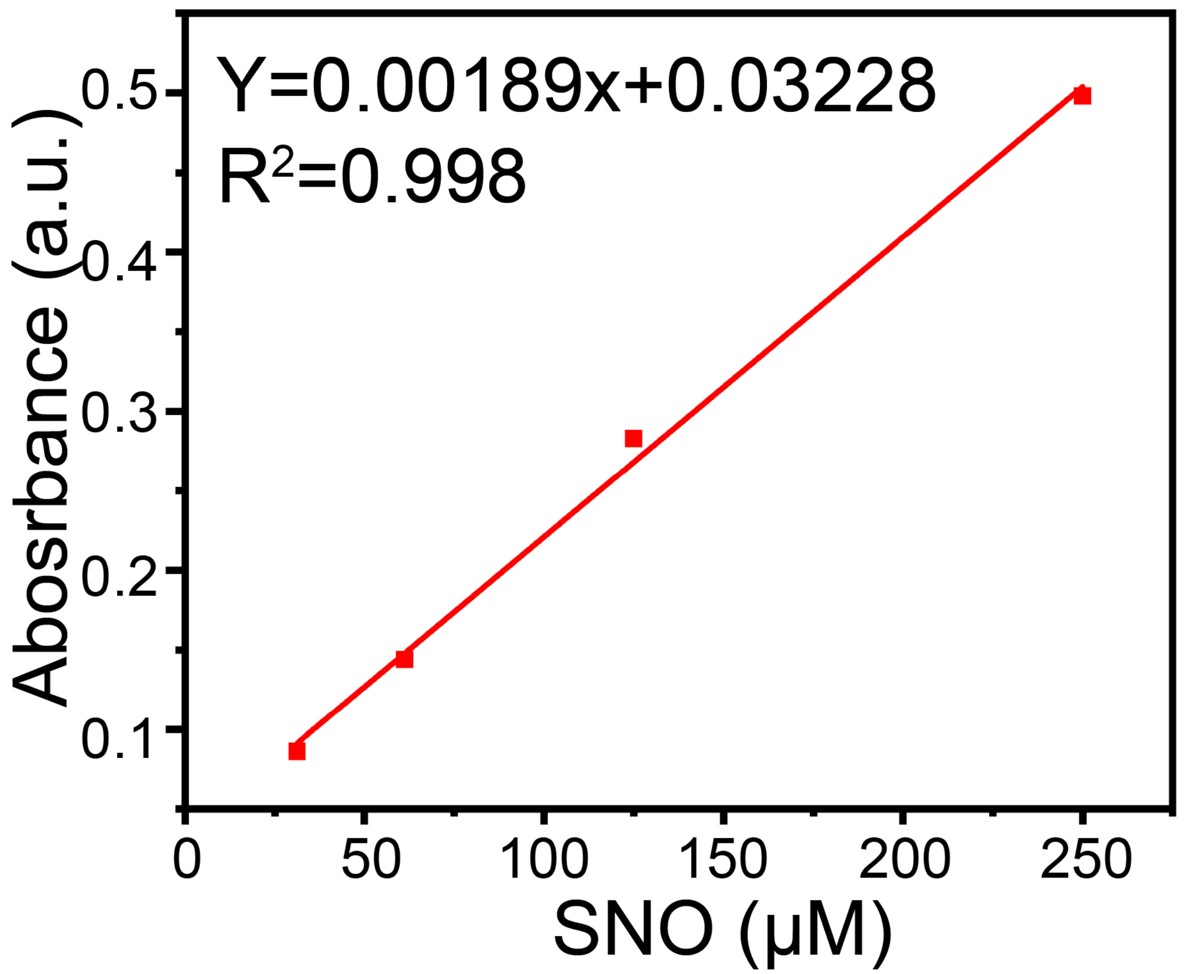

Figure S3. Plot of data from GSNO standard curve and measured by UV-visible spectrophotometry.

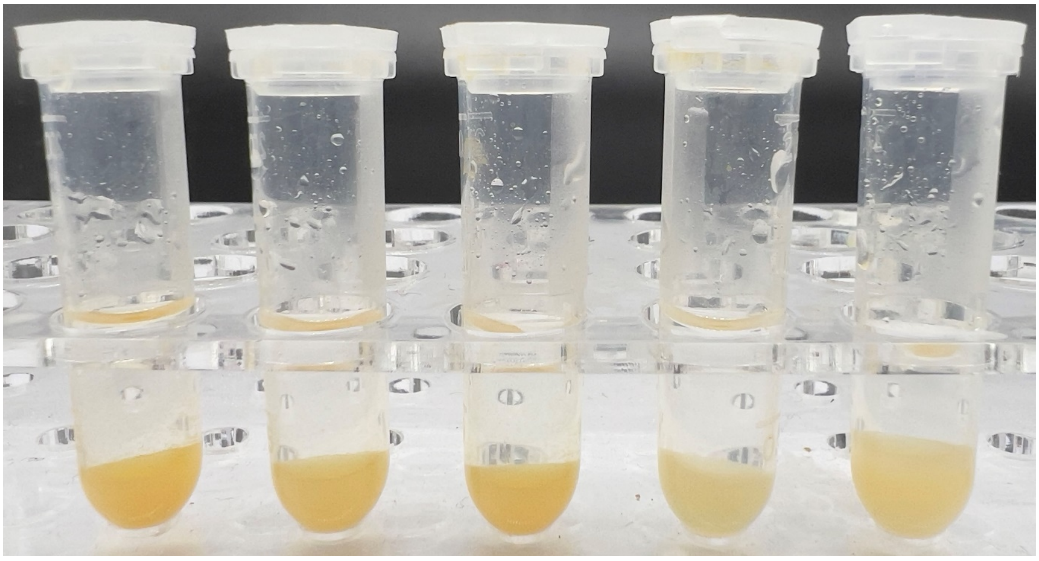

Figure S4. Photographs of FeGP1-5 dispersed in ultrapure water for 24 h. It is evident that FeGP1-5 samples exhibited substantial precipitation.

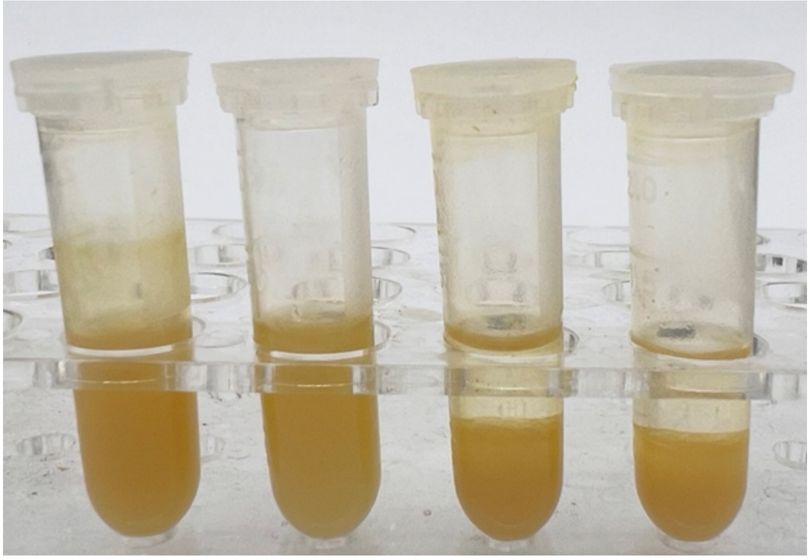

Figure S5. Photographs of LAPGP1-4 dispersed in ultrapure water for 24 h. It can be observed that there was no precipitation for LAPGP1-2, whereas LAPGP3-4 precipitated notably.

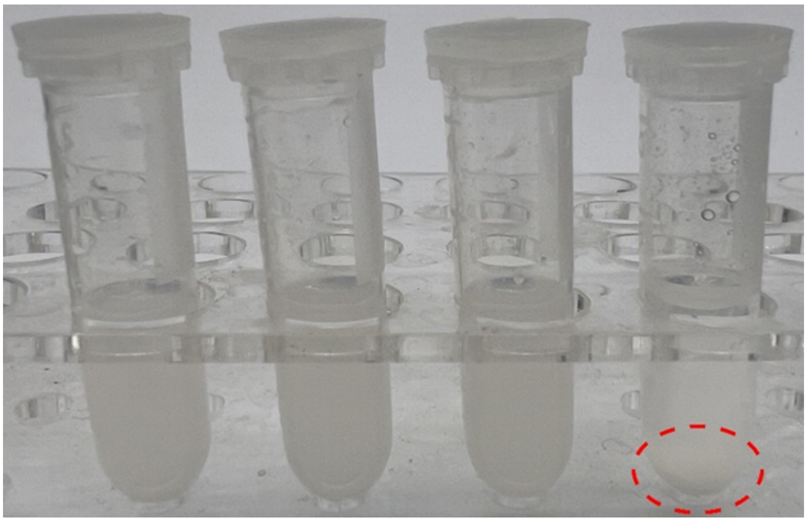

Figure S6. Photographs of BQRGP1-4 dispersed in ultrapure water for 24 h. It can be observed that there was no precipitation for BQRGP1-3, whereas BQRGP4 precipitated slightly.

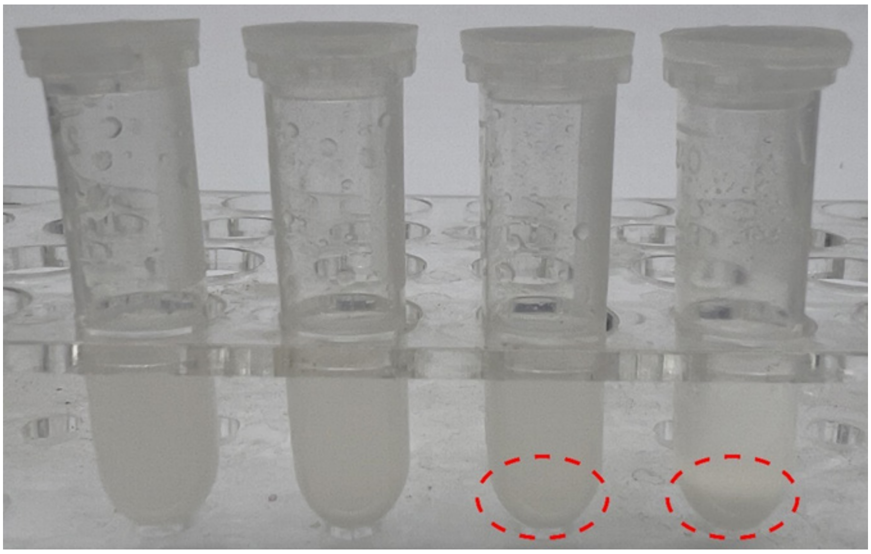

Figure S7. Photographs of SORGP1-5 dispersed in ultrapure water for 24 h. SORGP1-2 exhibited good water dispersibility without any precipitation. SORGP3-4 precipitated notably.

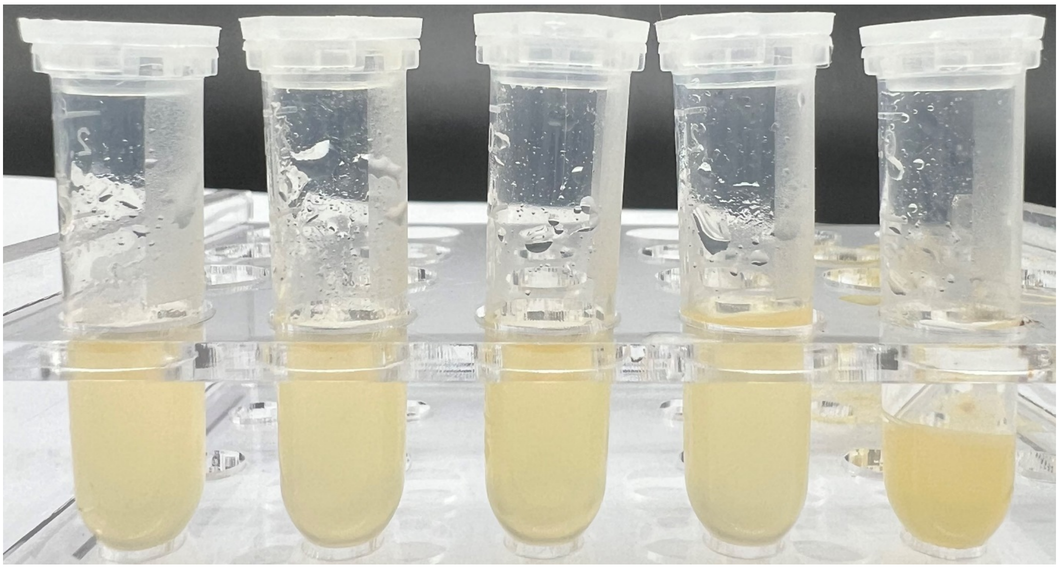

Figure S8. Photographs of FeGP1-5@HMON-SNO dispersed in ultrapure water for 24 h. FeGP1-4@HMON-SNO exhibited good water dispersibility without any precipitation. FeGP5@HMON-SNO precipitated slightly.

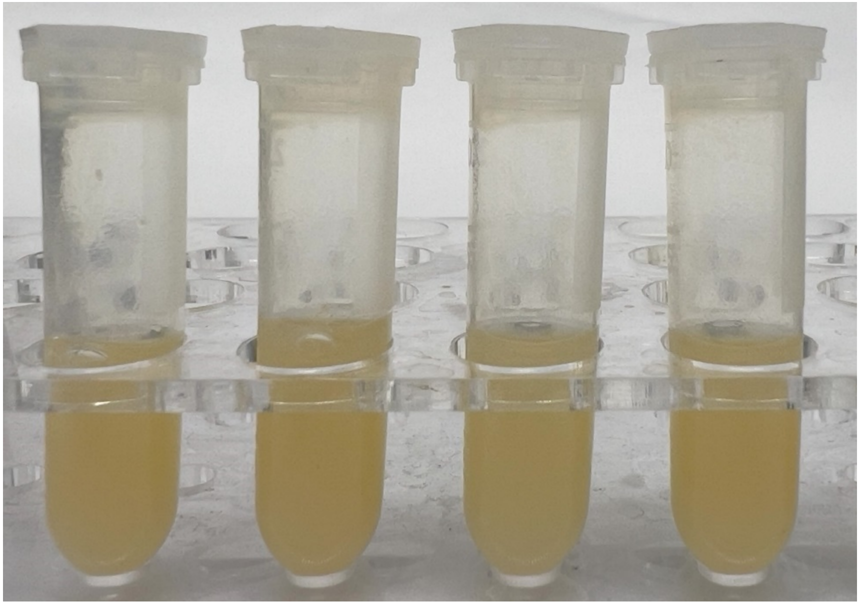

Figure S9. Photographs of LAPGP1-4@HMON-SNO dispersed in ultrapure water for 24 h. LAPGP1-4@HMON-SNO were all dispersed well without any precipitation.

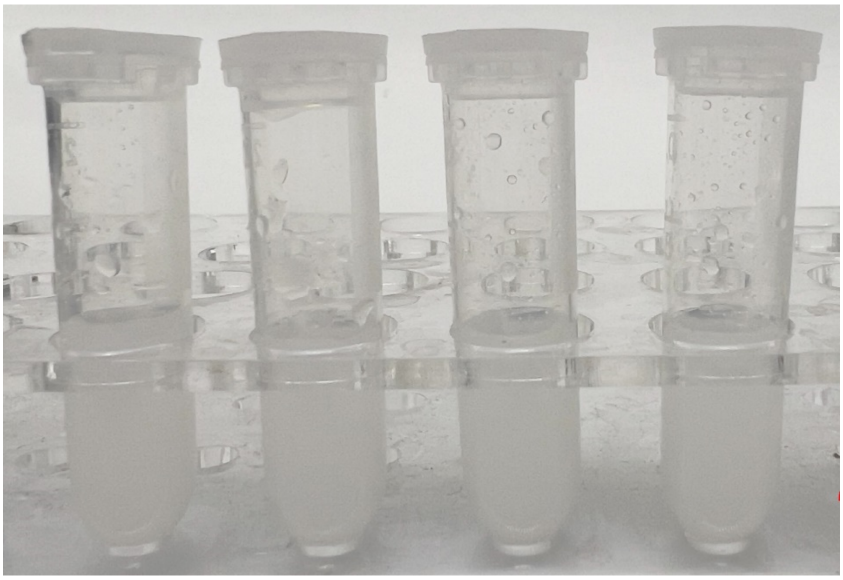

Figure S10. Photographs of BQRGP1-4@HMON-SNO dispersed in ultrapure water for 24 h. BQRGP1-4@HMON-SNO were all dispersed well without any precipitation.

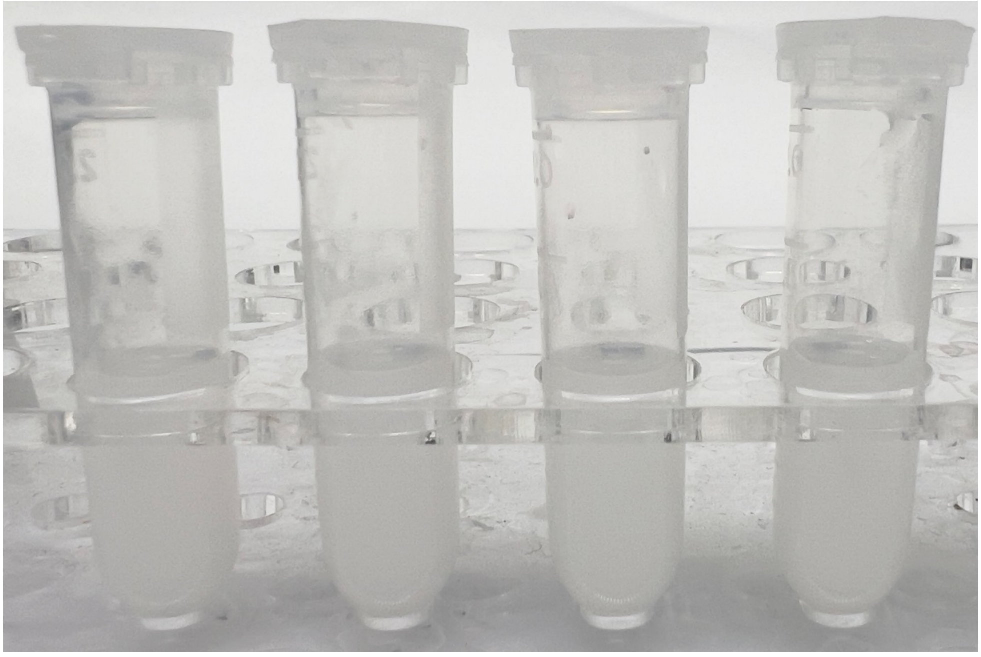

Figure S11. Photographs of SORGP1-4@HMON-SNO dispersed in ultrapure water for 24 h. SORGP1-4@HMON-SNO were all dispersed well without any precipitation.

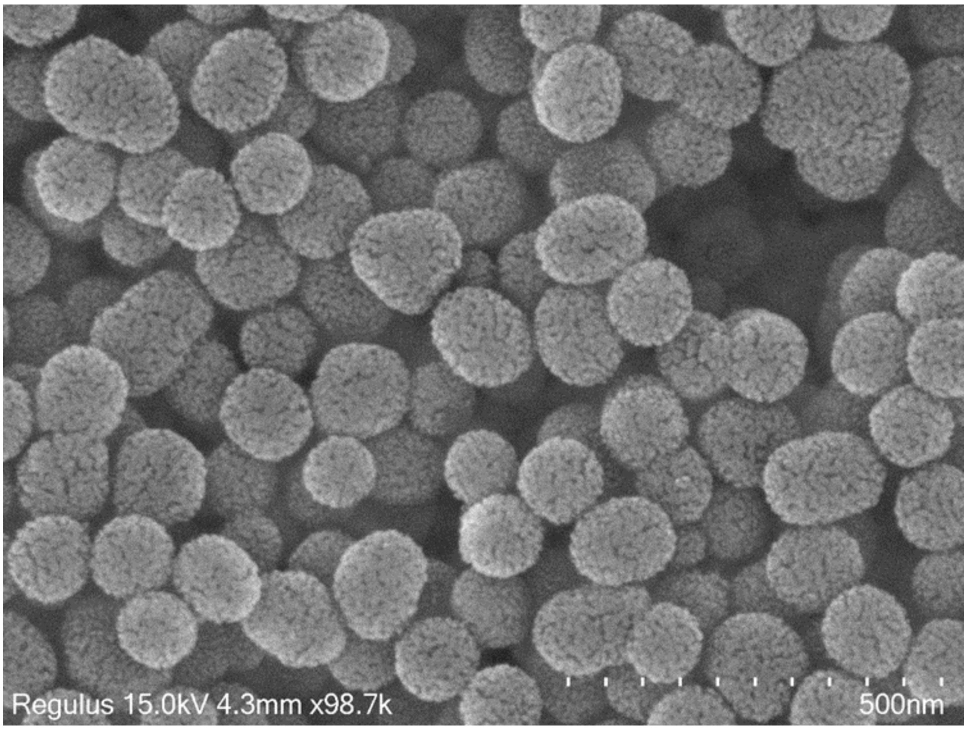

Figure S12. SEM image of FeGP4@HMON-SNO.

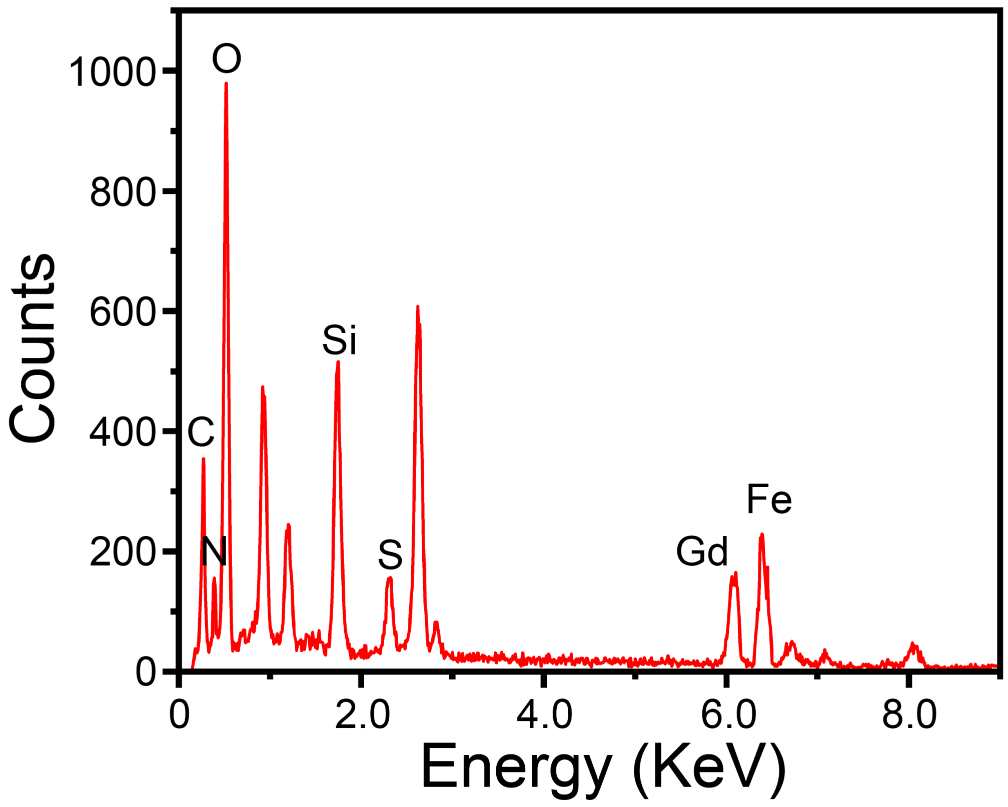

Figure S13. EDS spectrum of the FeGP4@HMON-SNO.

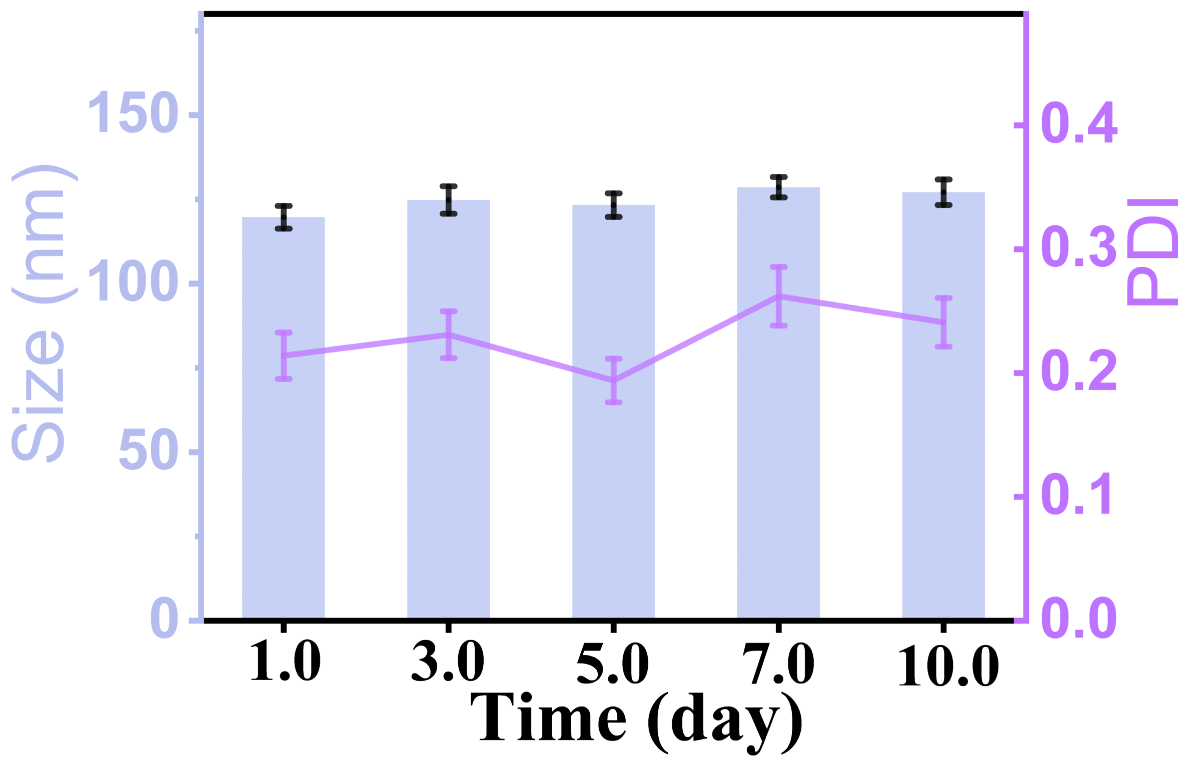

Figure S14. Hydrodynamic particle size and polydispersity index (PDI) of FeGP4@HMON-SNO in DMEM + 10% FBS within 10.0 days. Data are represented as mean ± S.D. (n = 3)

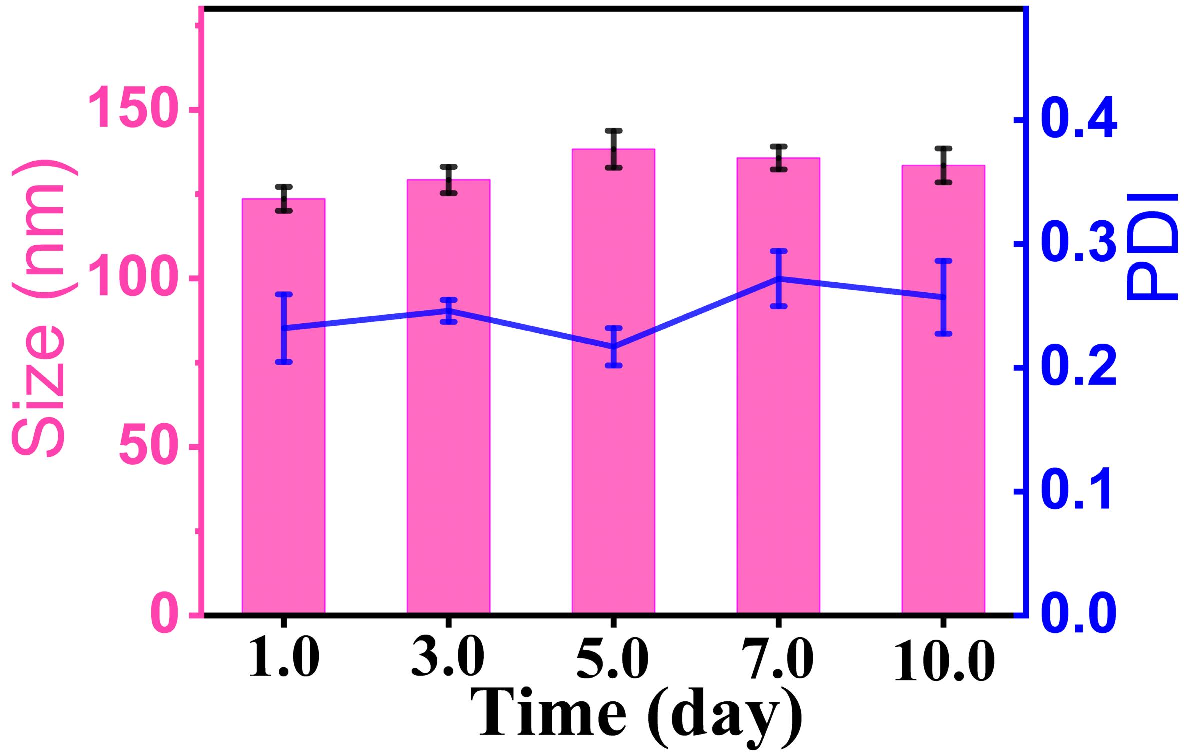

Figure S15. Hydrodynamic particle size and PDI of FeGP4@HMON-SNO in FBS within 10.0 days. Data are represented as mean ± S.D. (n = 3)

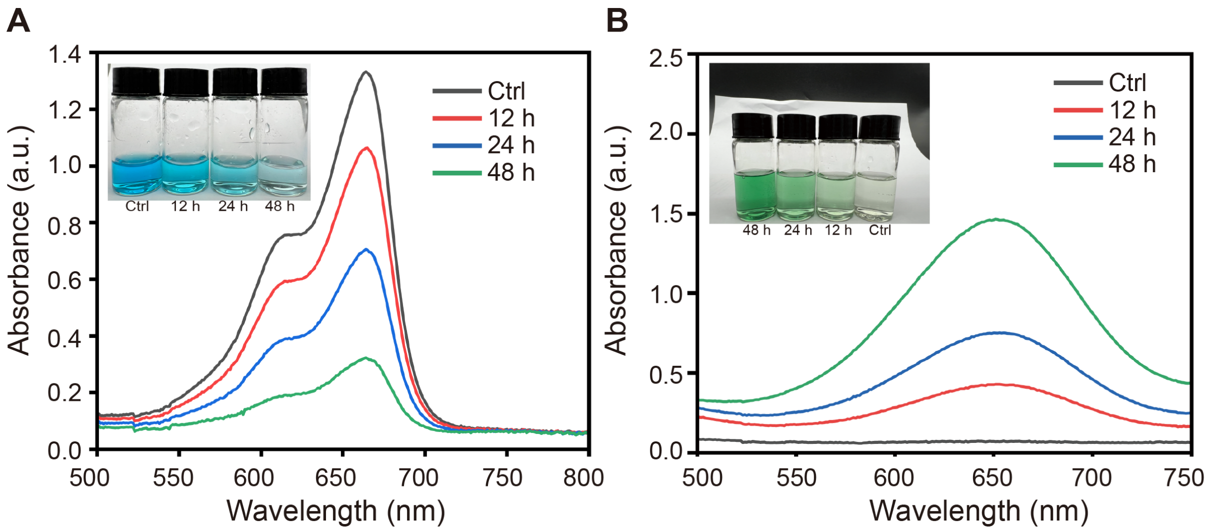

Figure S16. UV-Vis spectra and photographs (inset) of (A) MB solutions, and (B) TMB solutions incubated with FeGP4@HMON-SNO at different time, revealing the time-dependent ●OH generation.

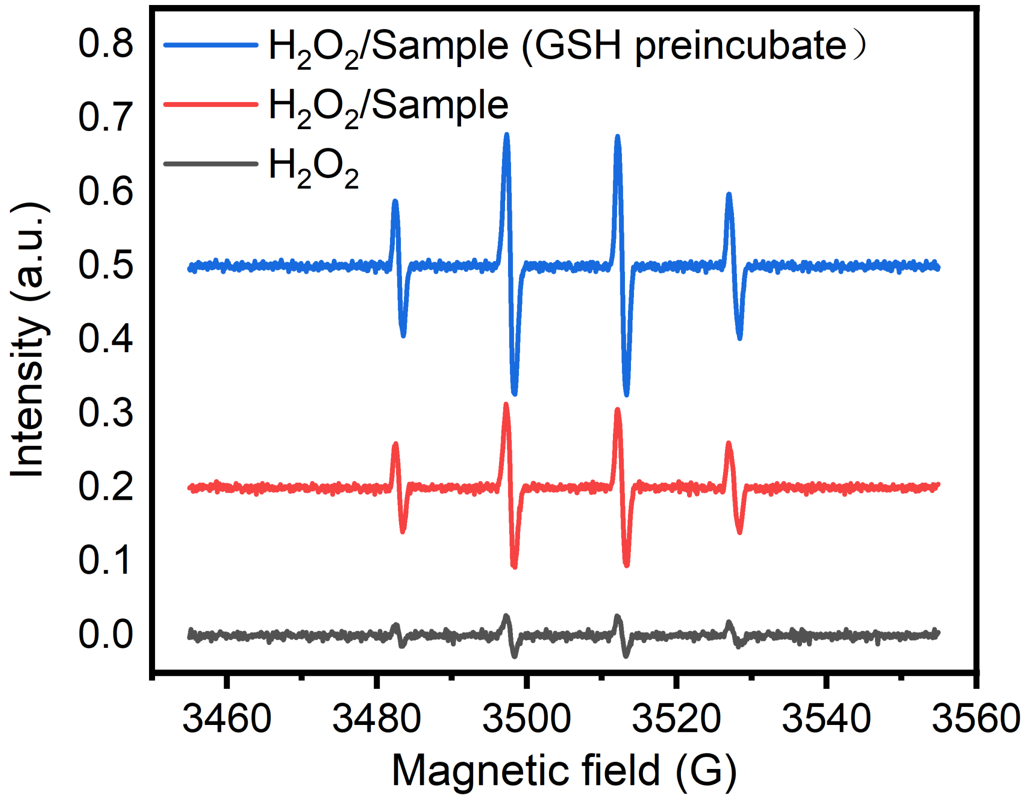

Figure S17. ESR spectra show ●OH generation from FeGP4@HMON-SNO pre-incubated with 10 mM of GSH at 37 ºC.

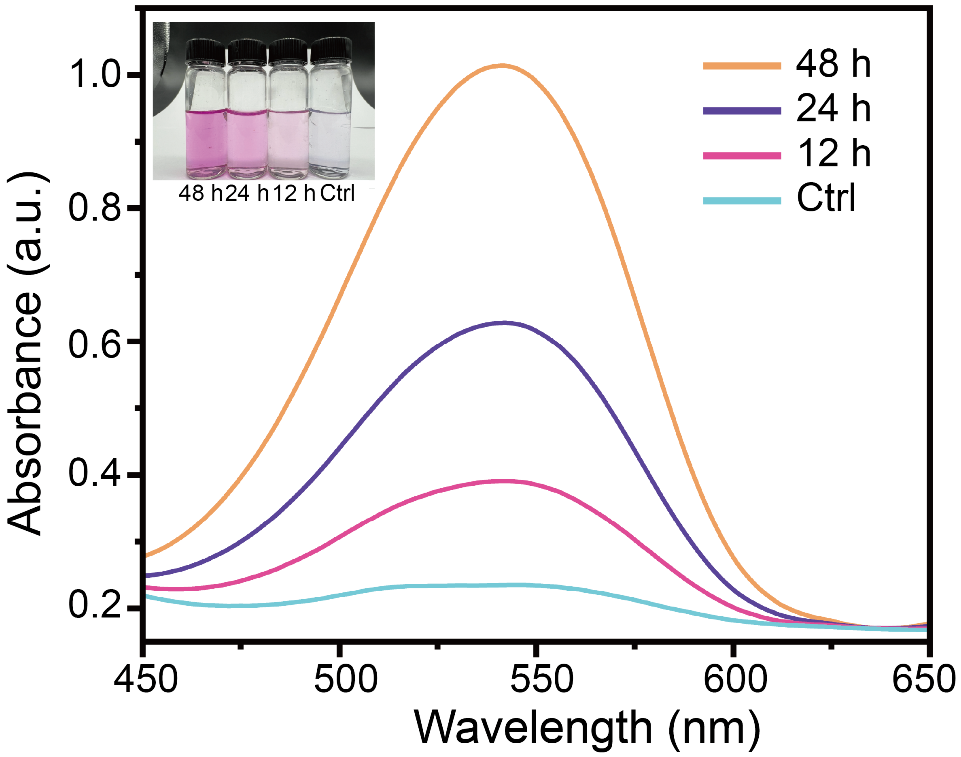

Figure S18. UV-Vis spectra and photographs (inset) of Griess agent after incubation with FeGP4@HMON-SNO for different time intervals in the presence of GSH (10 mM), showing the NO production capability of FeGP4@HMON-SNO.

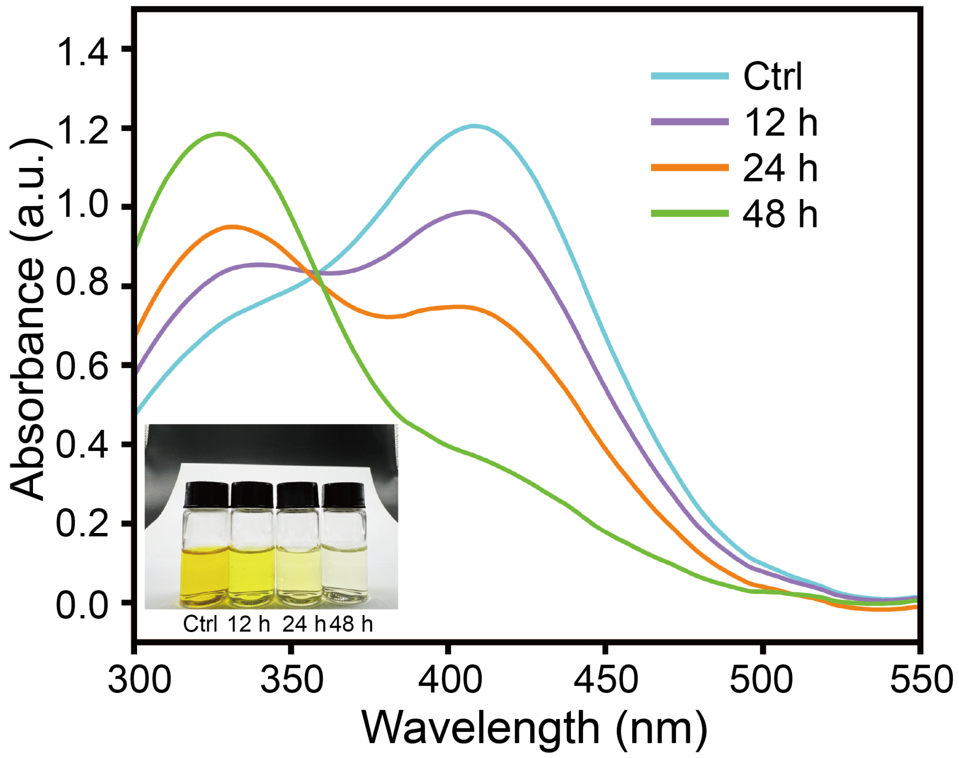

Figure S19. UV-Vis spectra and photographs (inset) of GSH after incubation with FeGP4@HMON-SNO for 0, 12, 24, or 48 h measured by DTNB assay, showing time dependence of GSH depletion abilities for FeGP4@HMON-SNO nanoparticles.

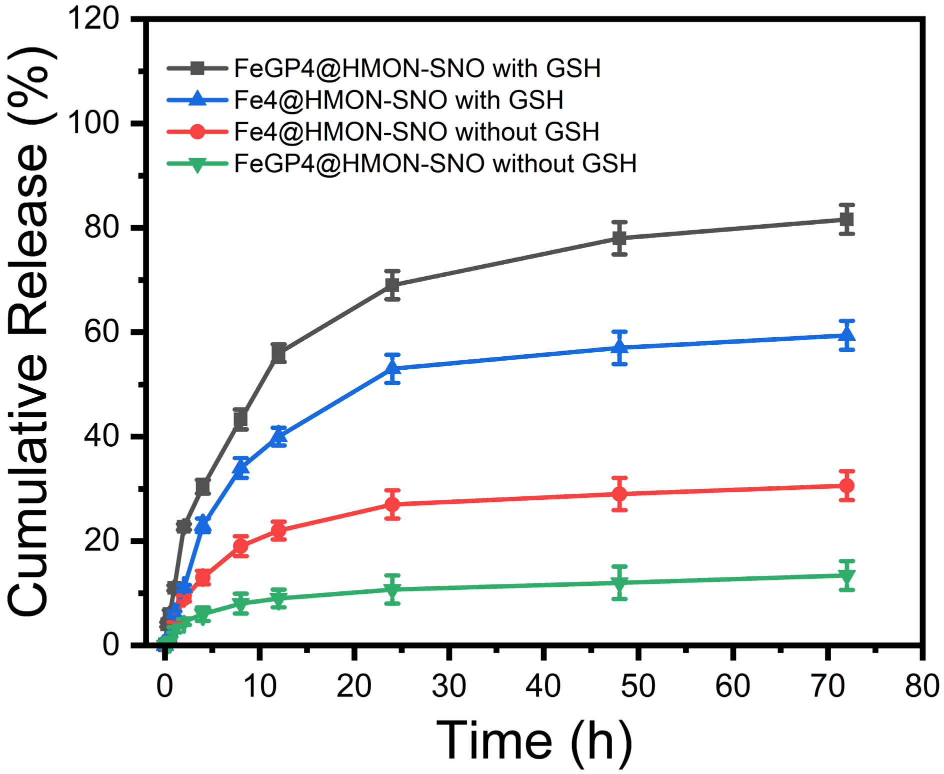

Figure S20. Cumulative release of Fe^{2+} from FeGP4@HMON-SNO and Fe4@HMON-SNO with or without GSH (10 mM).

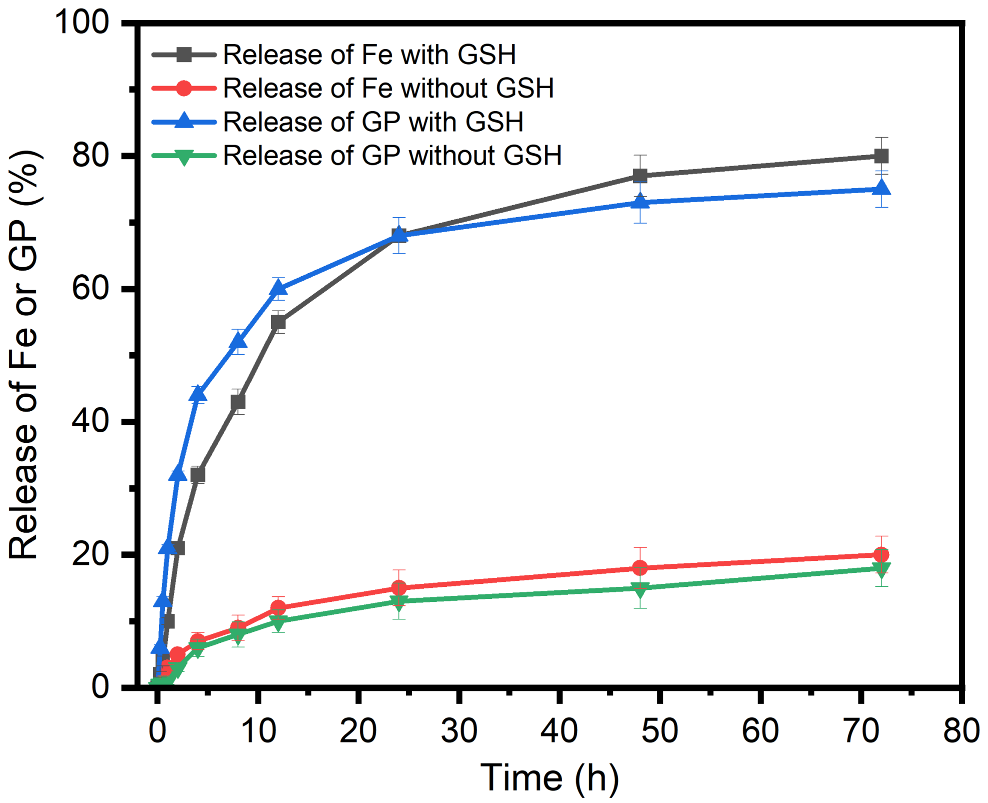

Figure S21. Cumulative release of Fe^{2+} or GP from FeGP4@HMON-SNO with or without GSH (10 mM).

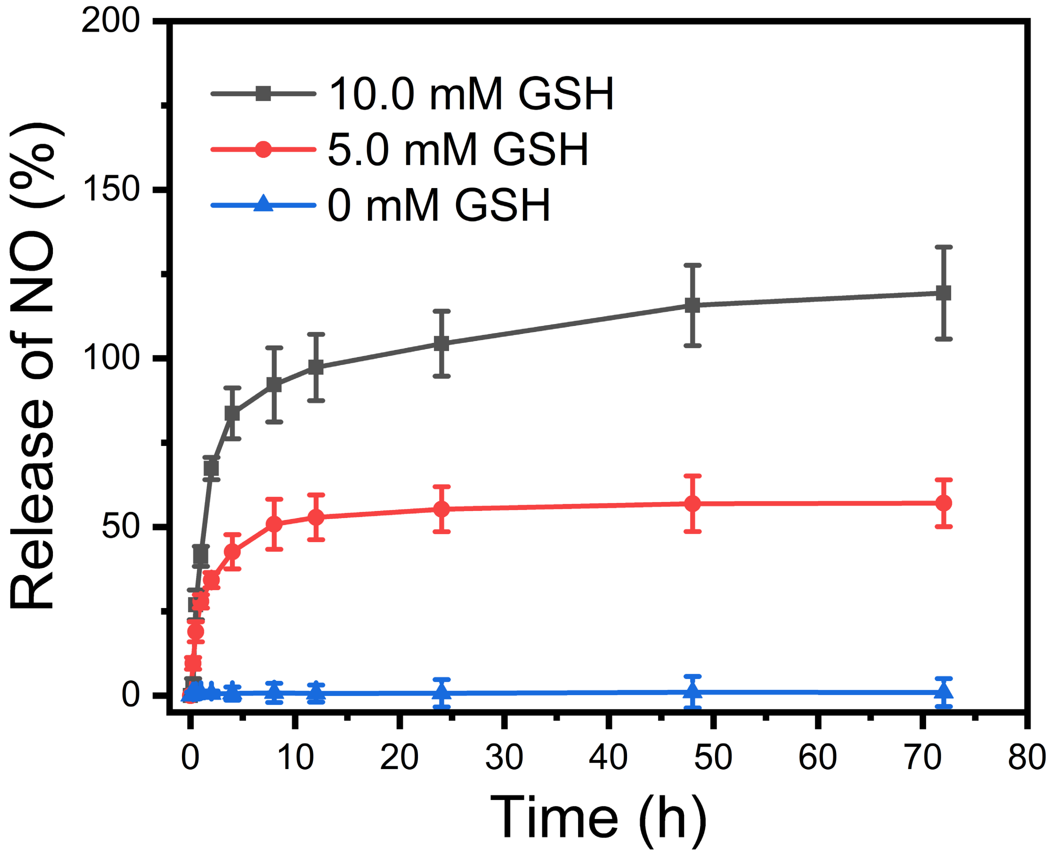

Figure S22. Release curves of NO from FeGP4@HMON-SNO in the presence of 0, 5.0, or 10.0 mM GSH.

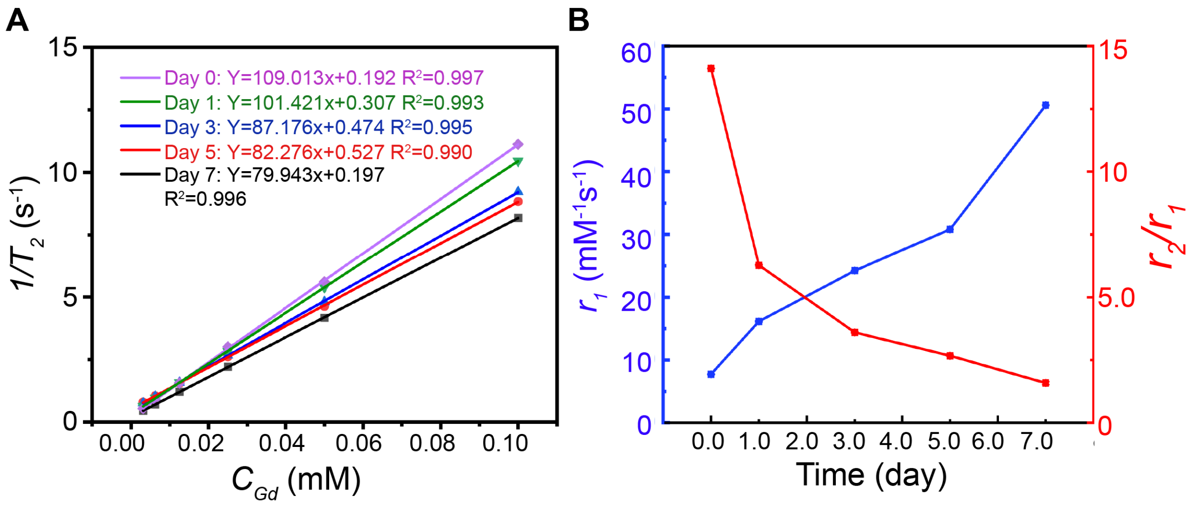

Figure S23. (A) T_2 relaxation rate ($1/T_2$) plotted as a function of C_{Gd} for FeGP4@HMON-SNO incubated with 10 mM of GSH at 37 °C for 0-7 days. TE=8.2 ms, TR=200 ms. (B) Change of r_1 or r_2/r_1 as a function of the incubation time for FeGP4@HMON-SNO. The magnetic field is 3.0 T.

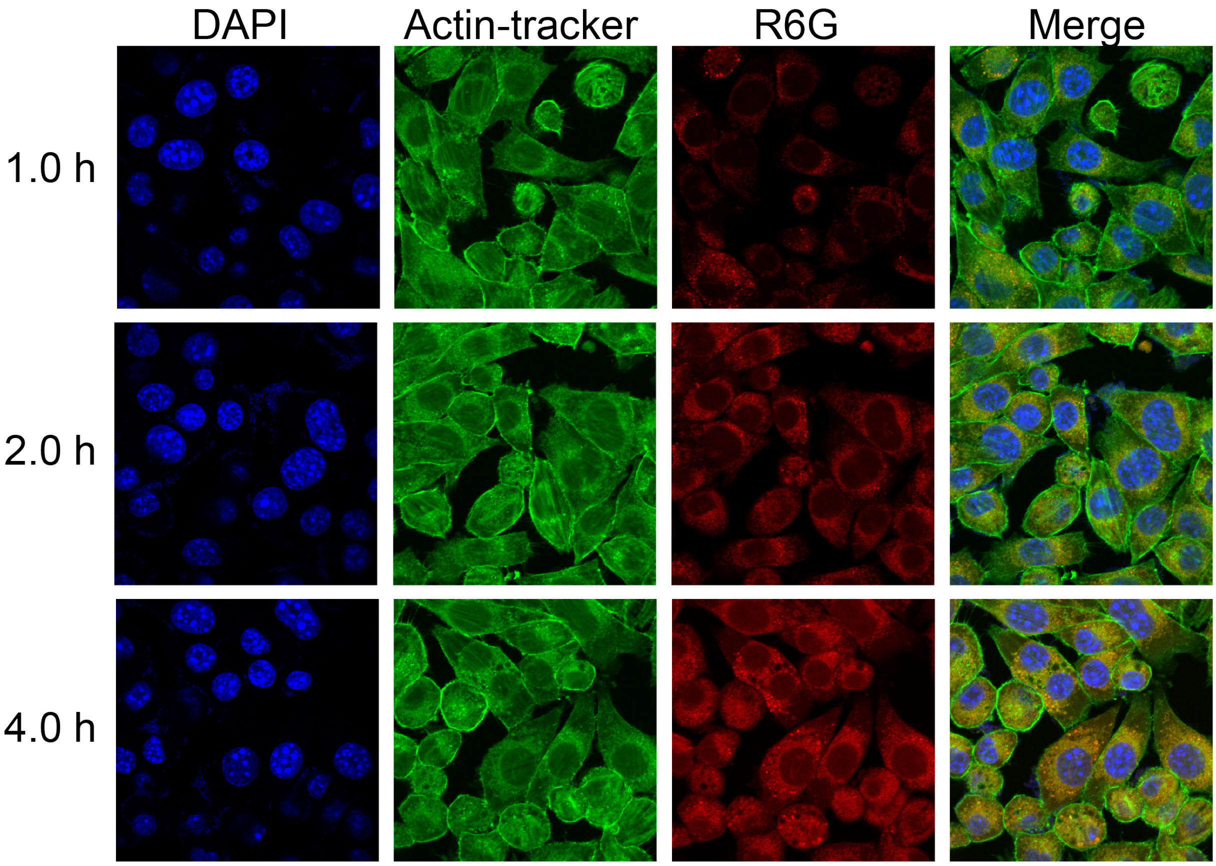

Figure S24. LSCM images of 4T1 cells after incubation with R6G-labelled FeGP4@HMON-SNO for different time intervals demonstrate the time-dependent cellular uptake capabilities of the nanoparticles. Green fluorescence: Actin-Tracker for cytoskeleton; red fluorescence: R6G for nanoparticles; blue fluorescence: DAPI for nuclei.

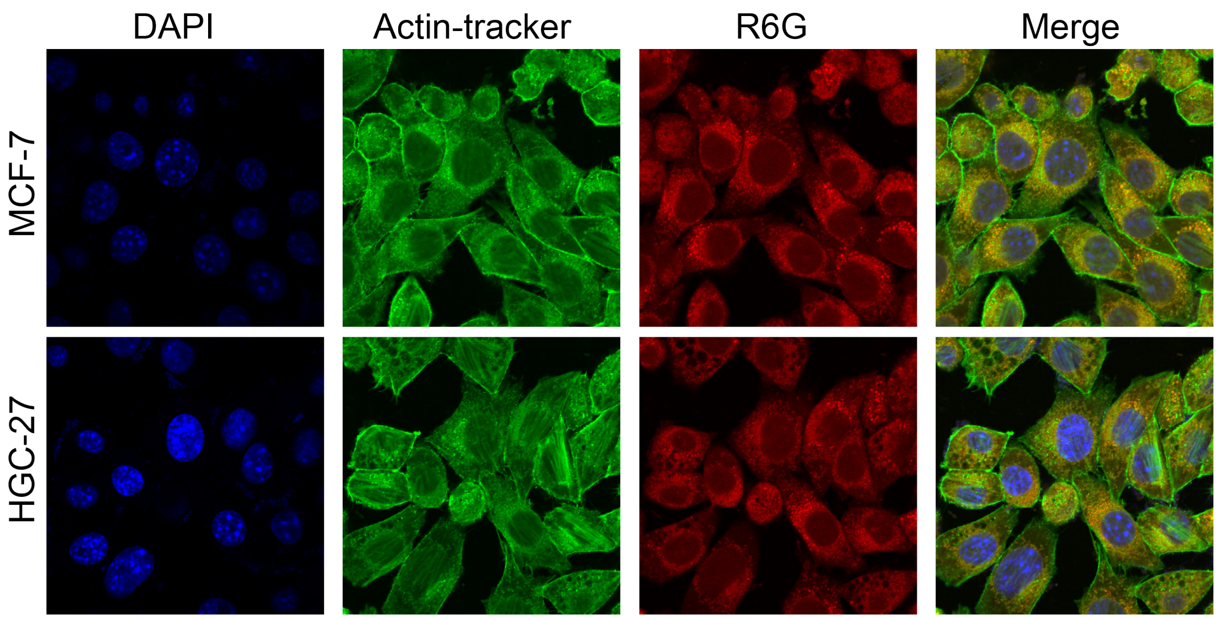

Figure S25. LSCM images of HGC27 or MCF7 cells after incubation with R6G-labelled FeGP4@HMON-SNO for 4.0 h demonstrate the uptake capabilities of the nanoparticles in different cell types. Green fluorescence: Actin-Tracker for cytoskeleton; red fluorescence: R6G for nanoparticles; blue fluorescence: DAPI for nuclei.

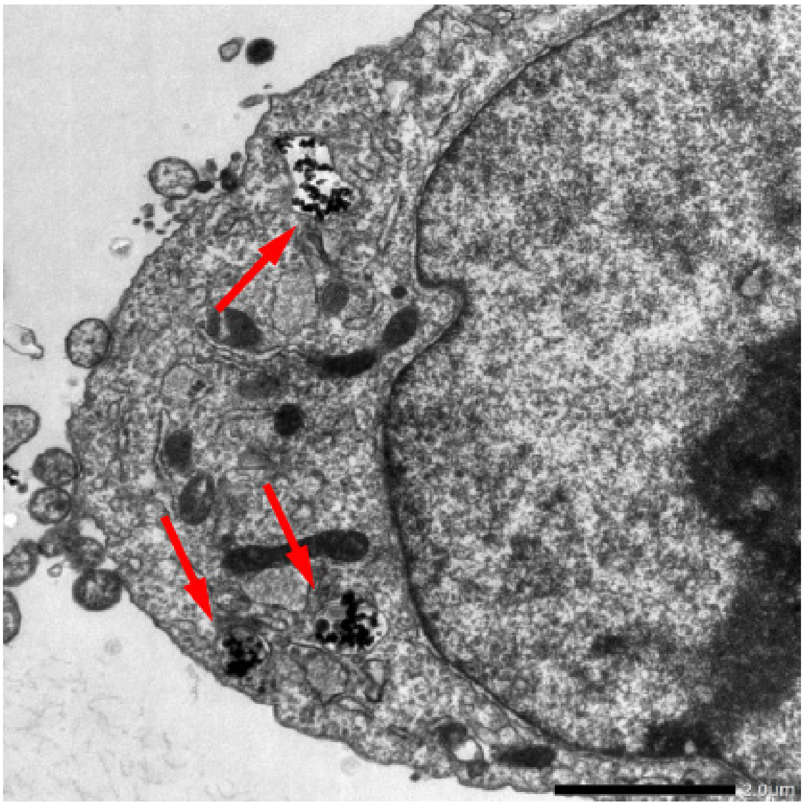

Figure S26. Bio-TEM images of 4T1 cells treated with FeGP4@HMON-SNO for 4.0 h.

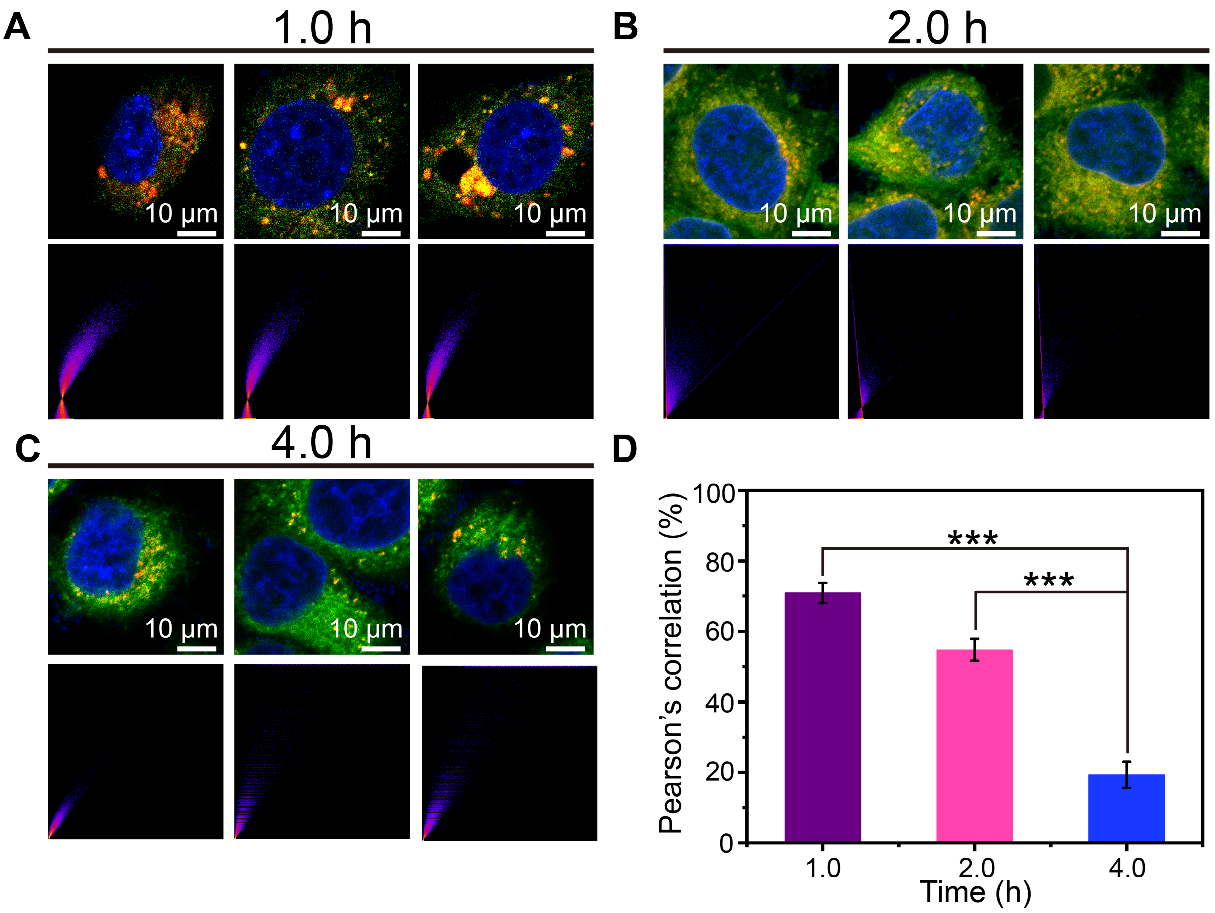

Figure S27. Illustrative co-localization separate channel images of FeGP4@HMON-SNO in lysosomes at 1.0 h (A), 2.0 h (B), and 4.0 h (C) using LSCM. Yellow fluorescence represents colocalization of Fe^{2+} (green) and FITC-RA (red). Pearson's correlation at different time were shown in (D).

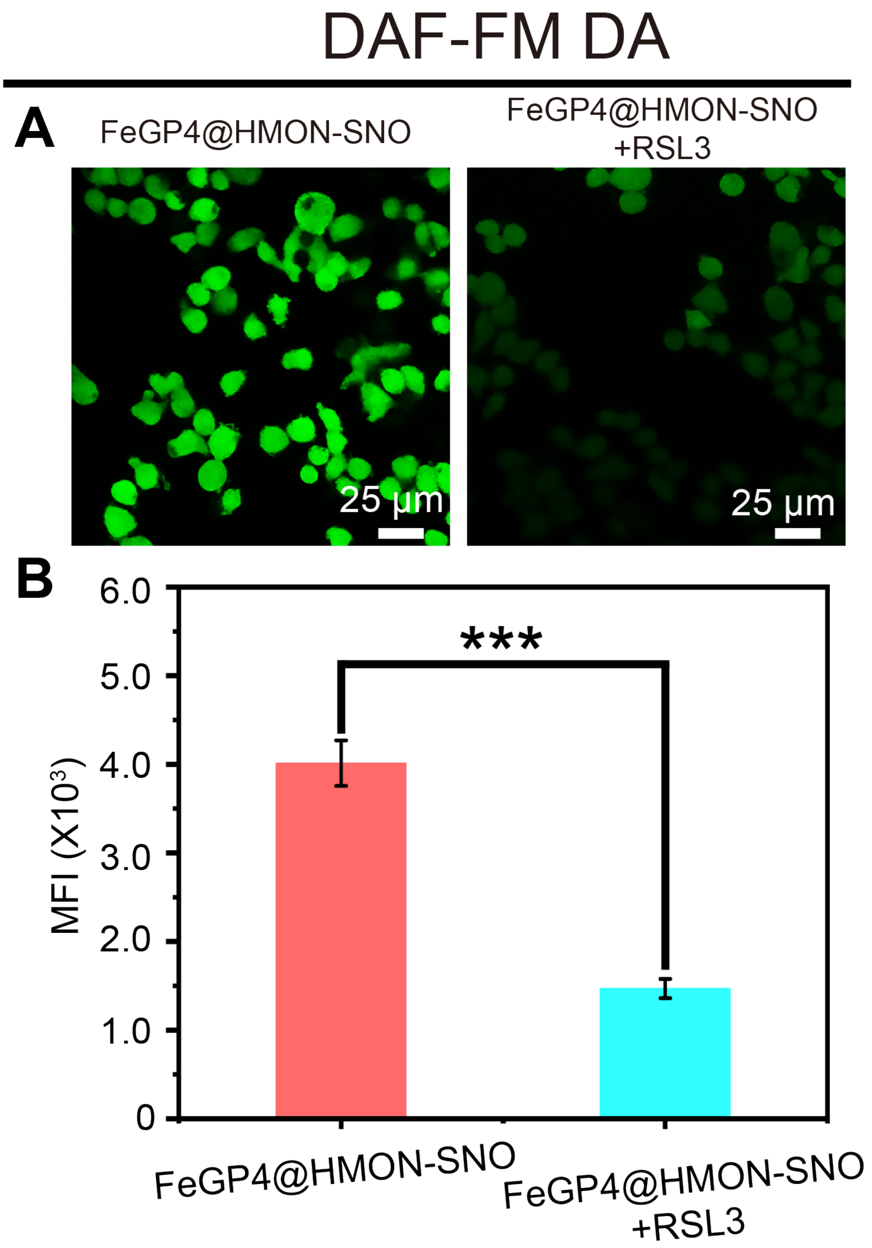

Figure S28. (A) LSCM images of 4T1 cells after incubation with FeGP4@HMON-SNO, with or without pretreatment of RSL3, and stained with DAF-FM DA. (B) The corresponding quantitative analysis.

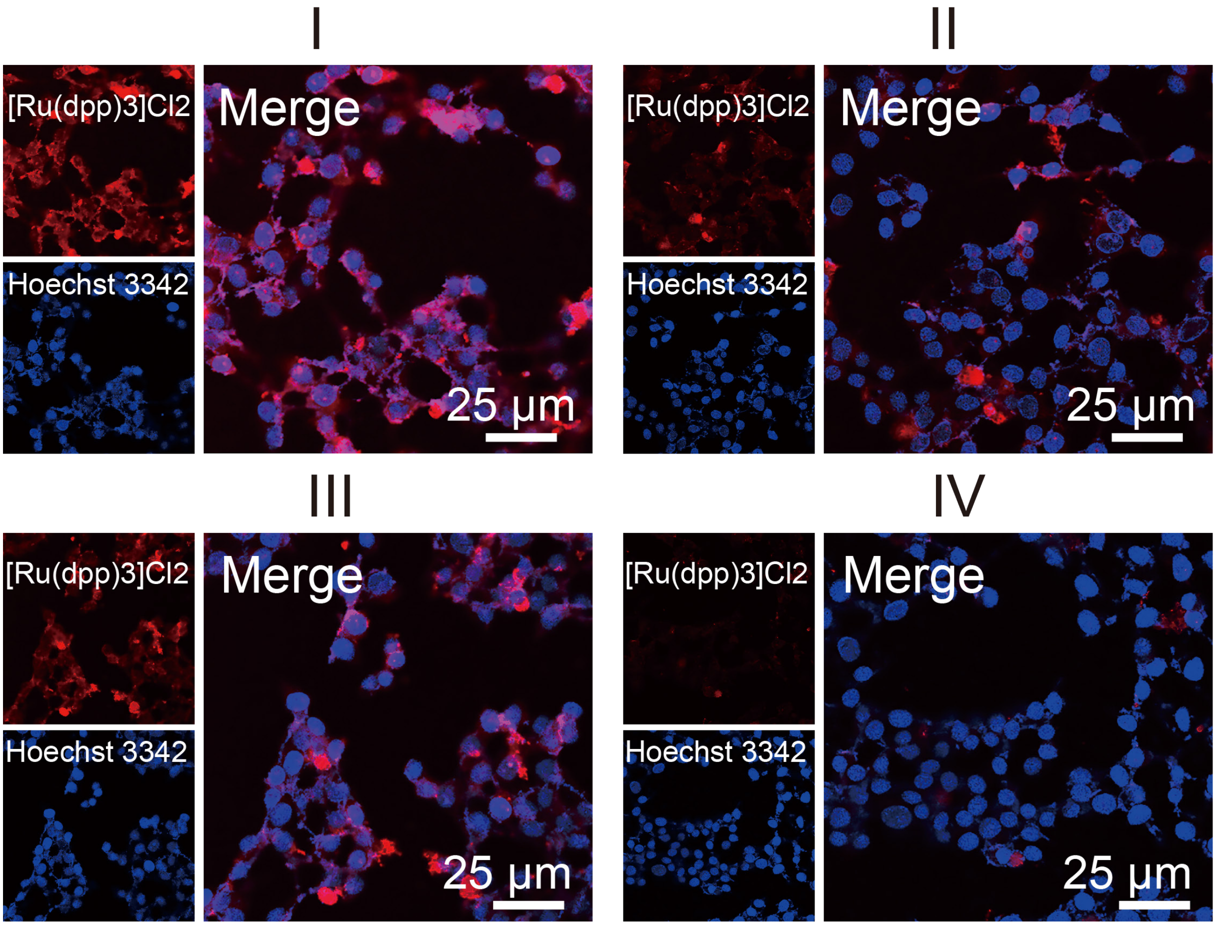

Figure S29. LSCM images of 4T1 cells after incubation with PBS (I), HMON-SNO (II), FeGP4@HMON (III), or FeGP4@HMON-SNO (IV), and staining with $[Ru(DPP)_3]Cl_2$ probe, showing intracellular O_2 level.

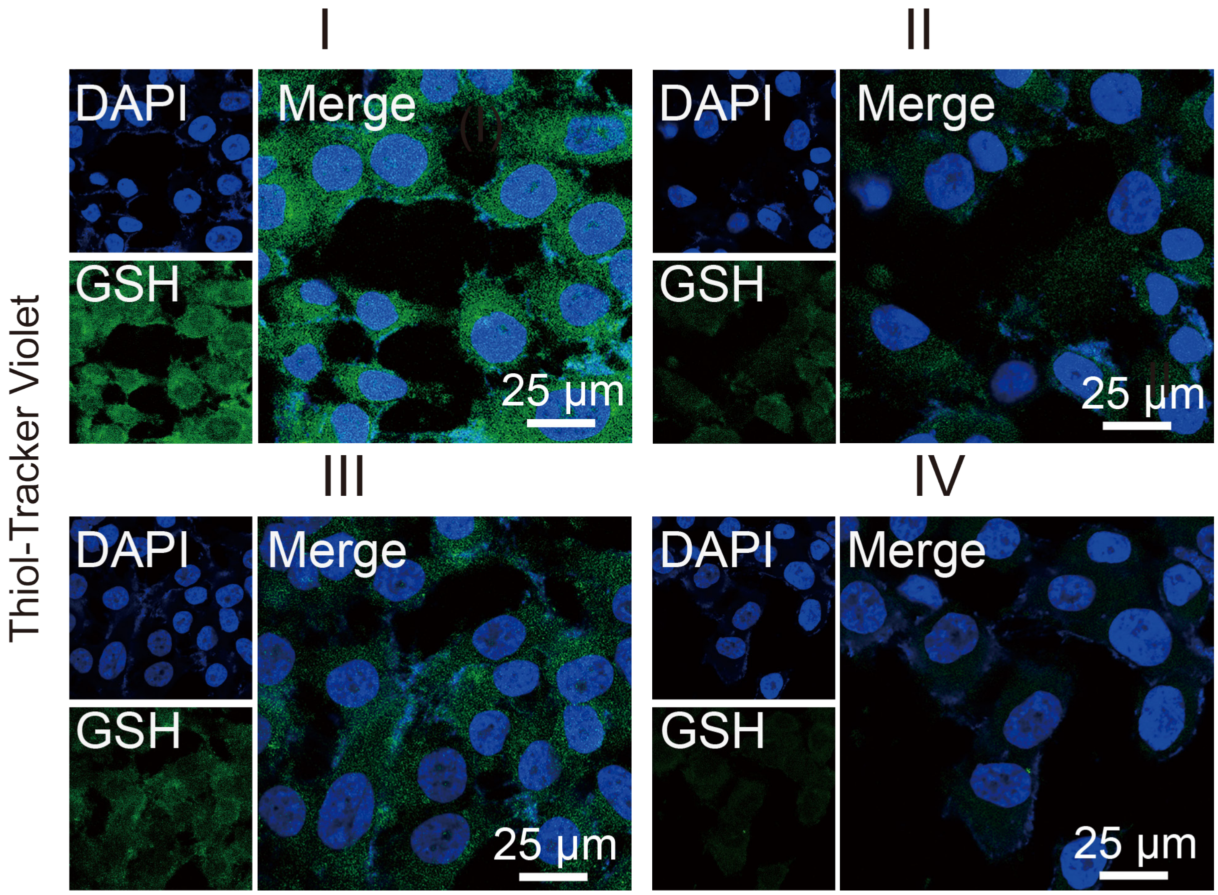

Figure S30. LSCM images of 4T1 cells after incubation with PBS (I), HMON-SNO (II), FeGP4@HMON (III), FeGP4@HMON-SNO (IV), and staining with Thiol-Tracker Violet probe, showing intracellular GSH content.

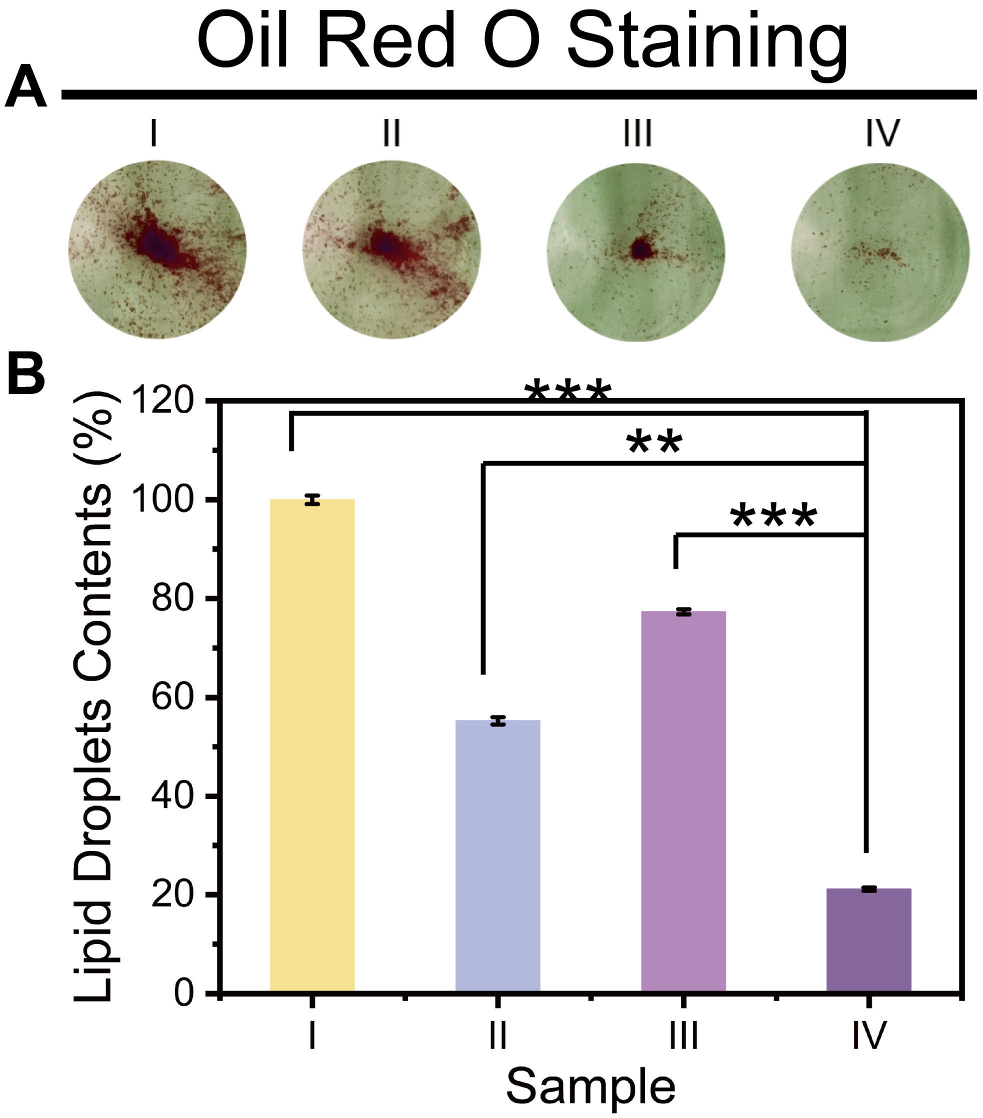

Figure S31. Optical microscope images of lipid droplets in 4T1 cells after treatments with PBS (I), HMON-SNO (II), FeGP4@HMON (III), or FeGP4@HMON-SNO (IV) staining with Oil red O (A), and the corresponding quantitative analysis of the lipid droplet (B). Mean ± SD, n = 3. ** $P < 0.01$, *** $P < 0.001$.

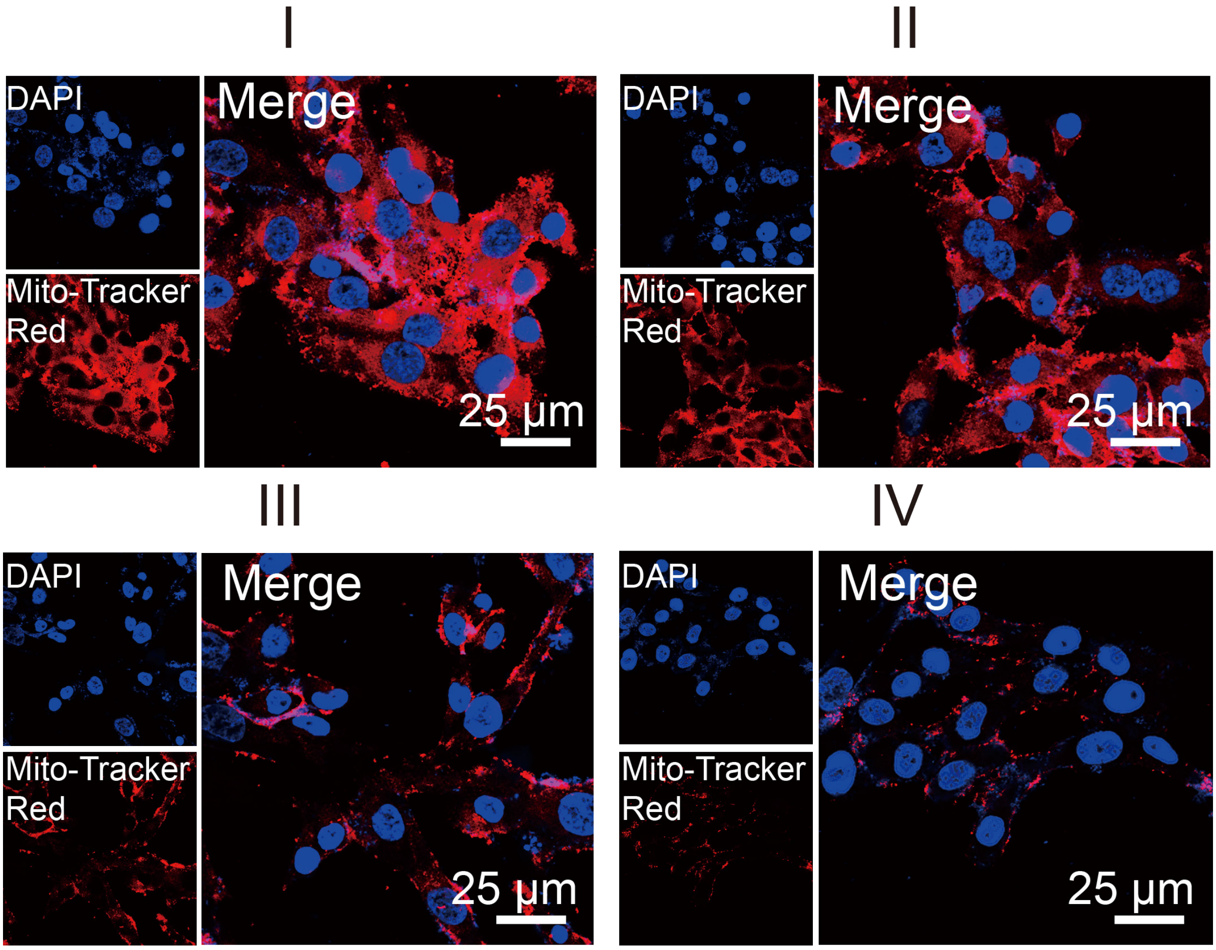

Figure S32. LSCM images of 4T1 cells after incubation with PBS (I), HMON-SNO (II), FeGP4@HMON (III), or FeGP4@HMON-SNO (IV), and staining with Mito-Tracker Red probe, showing the mitochondrial damage level.

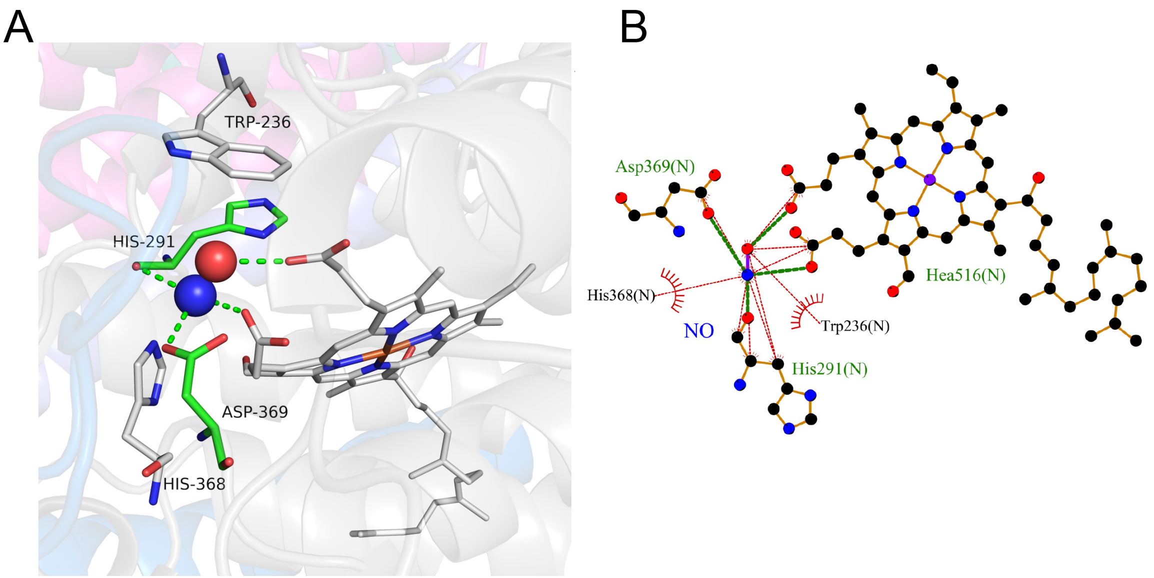

Figure S33. Schematic representation of binding modes for small molecules and proteins. The two-dimensional binding mode of the small molecule with the protein (A), and its three-dimensional binding mode (B). Green dashed lines indicate hydrogen bonding interactions.

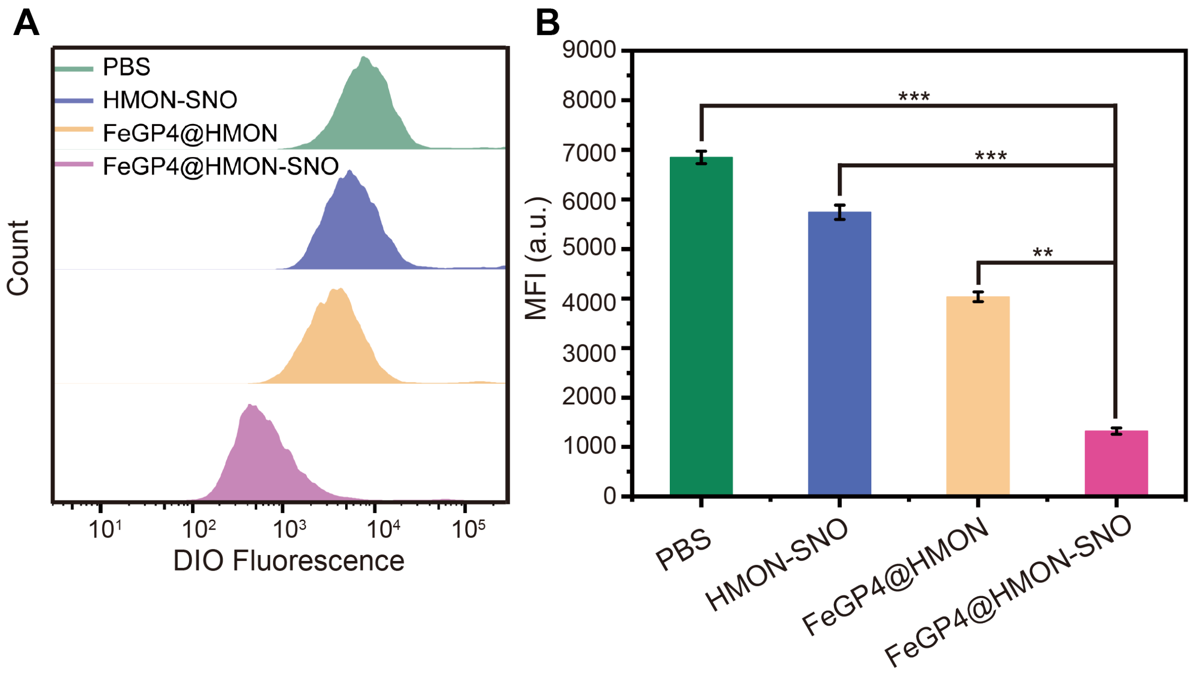

Figure S34. DIO fluorescence distributions (A), and the corresponding quantitative analysis (B) of 4T1 cells after treatments with PBS (I), HMON-SNO (II), FeGP4@HMON (III), or FeGP4@HMON-SNO (IV) determined by flow cytometry. Mean ± SD, n = 3. ** $P < 0.01$, *** $P < 0.001$.

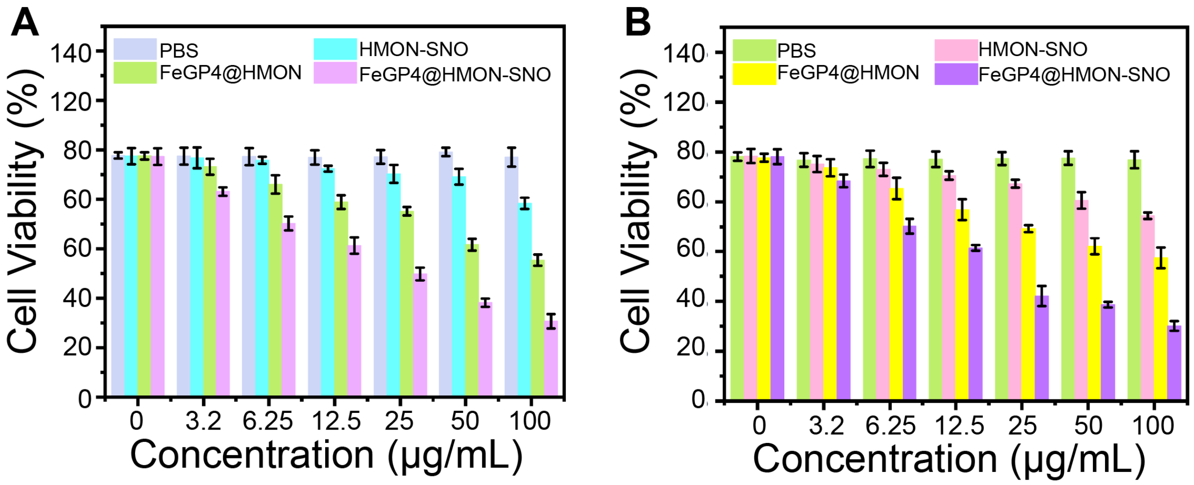

Figure S35. Cell viability of MCF7 cells (A), and HGC27 cells (B) after various treatments for 24 h.

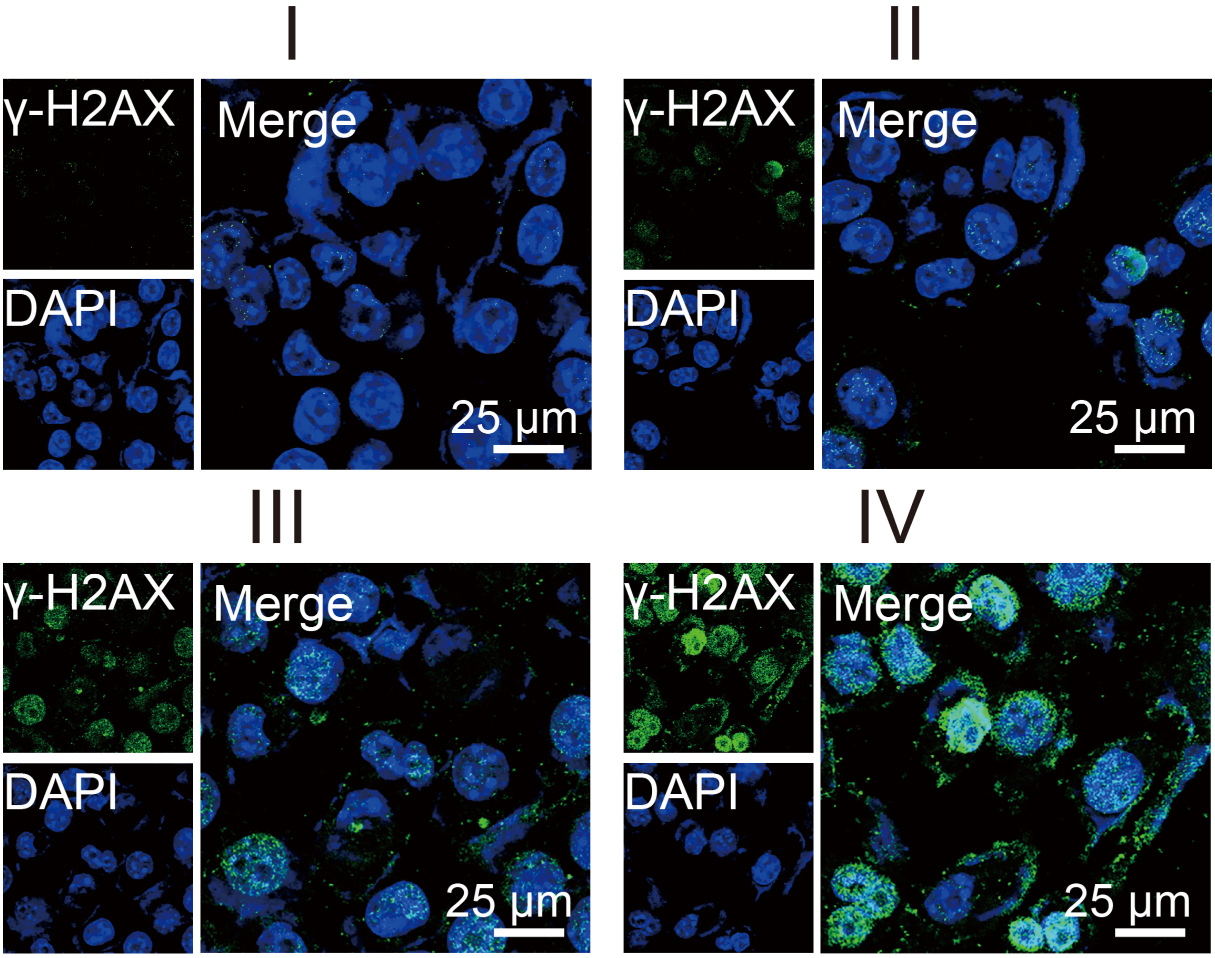

Figure S36. LSCM images of 4T1 cells after incubation with PBS (I), HMON-SNO (II), FeGP4@HMON (III), or FeGP4@HMON-SNO (IV), and staining with γ-H2AX probe, showing the DNA damage level.

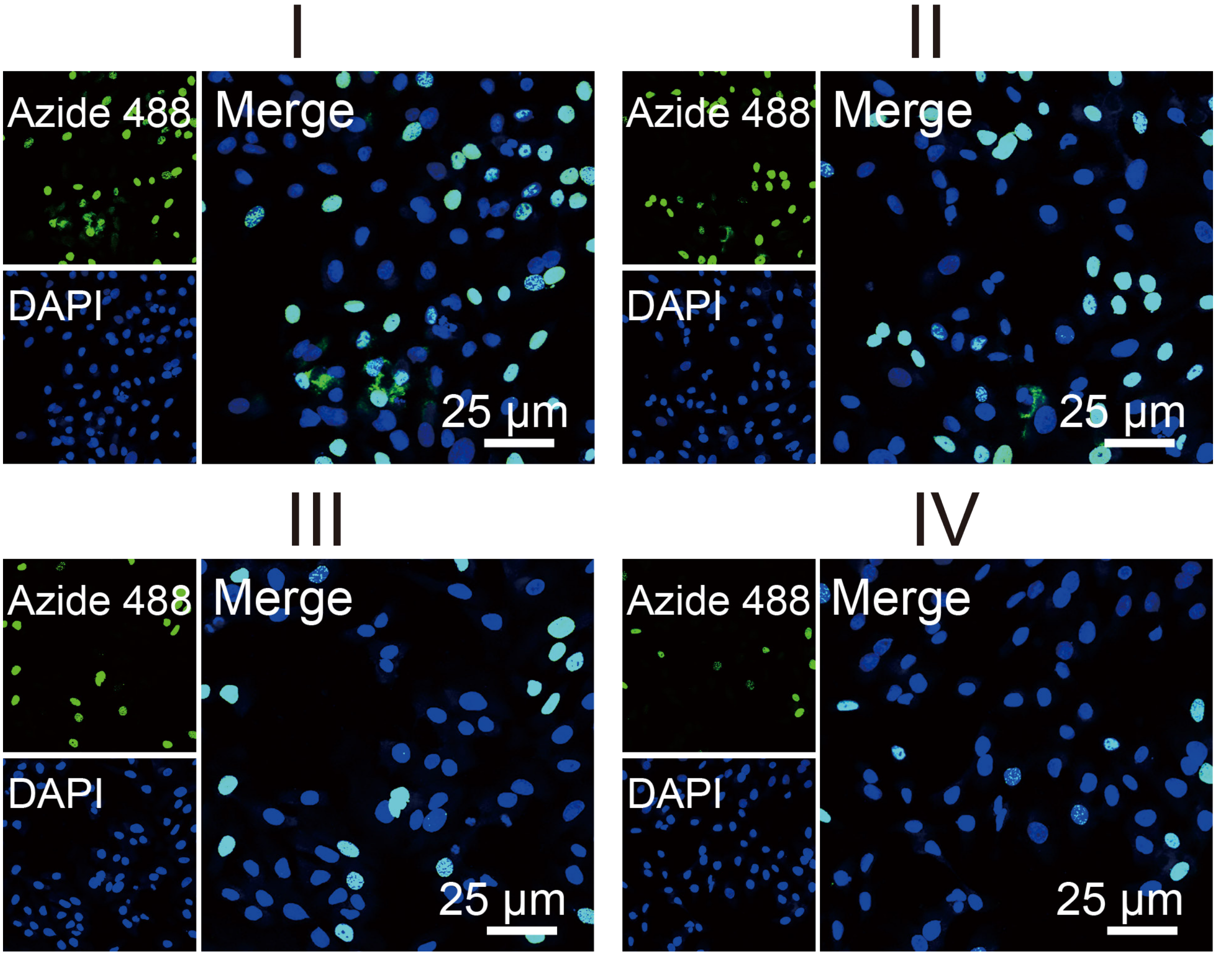

Figure S37. LSCM images of 4T1 cells after incubation with PBS (control), HMON-SNO (II), FeGP4@HMON (III), or FeGP4@HMON-SNO (IV), and staining with Azide 488 probe, indicating the level of cell proliferation.

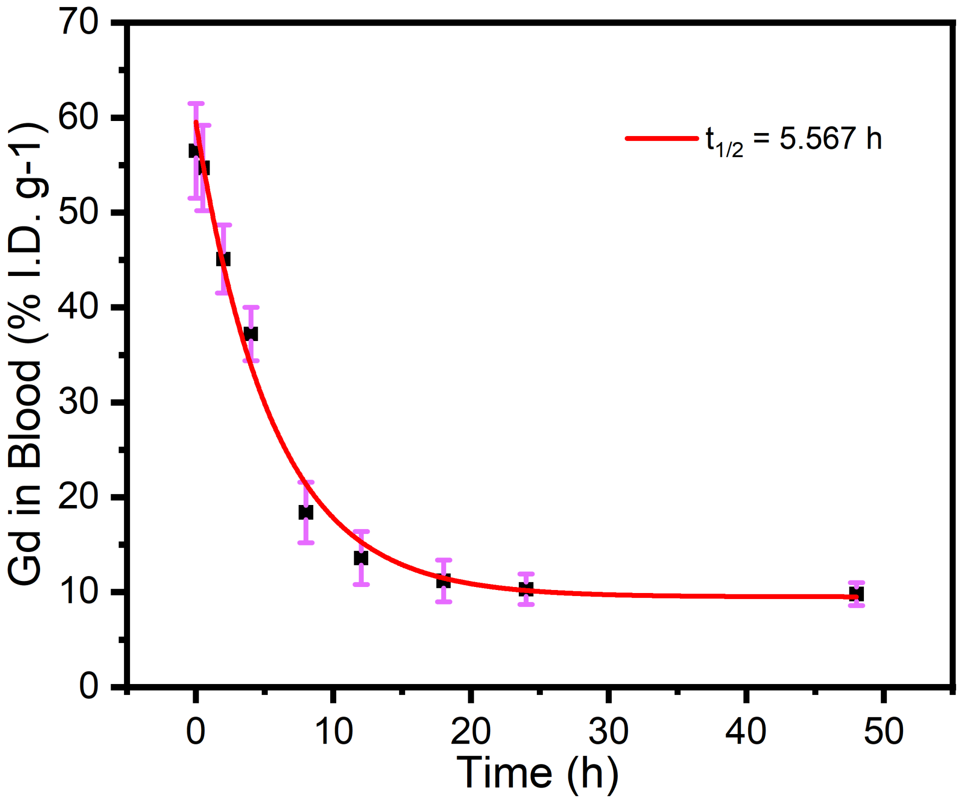

Figure S38. Pharmacokinetic profile of FeGP4@HMON-SNO intravenously injected into mice (C_{Gd} = 5.0 mg/kg).

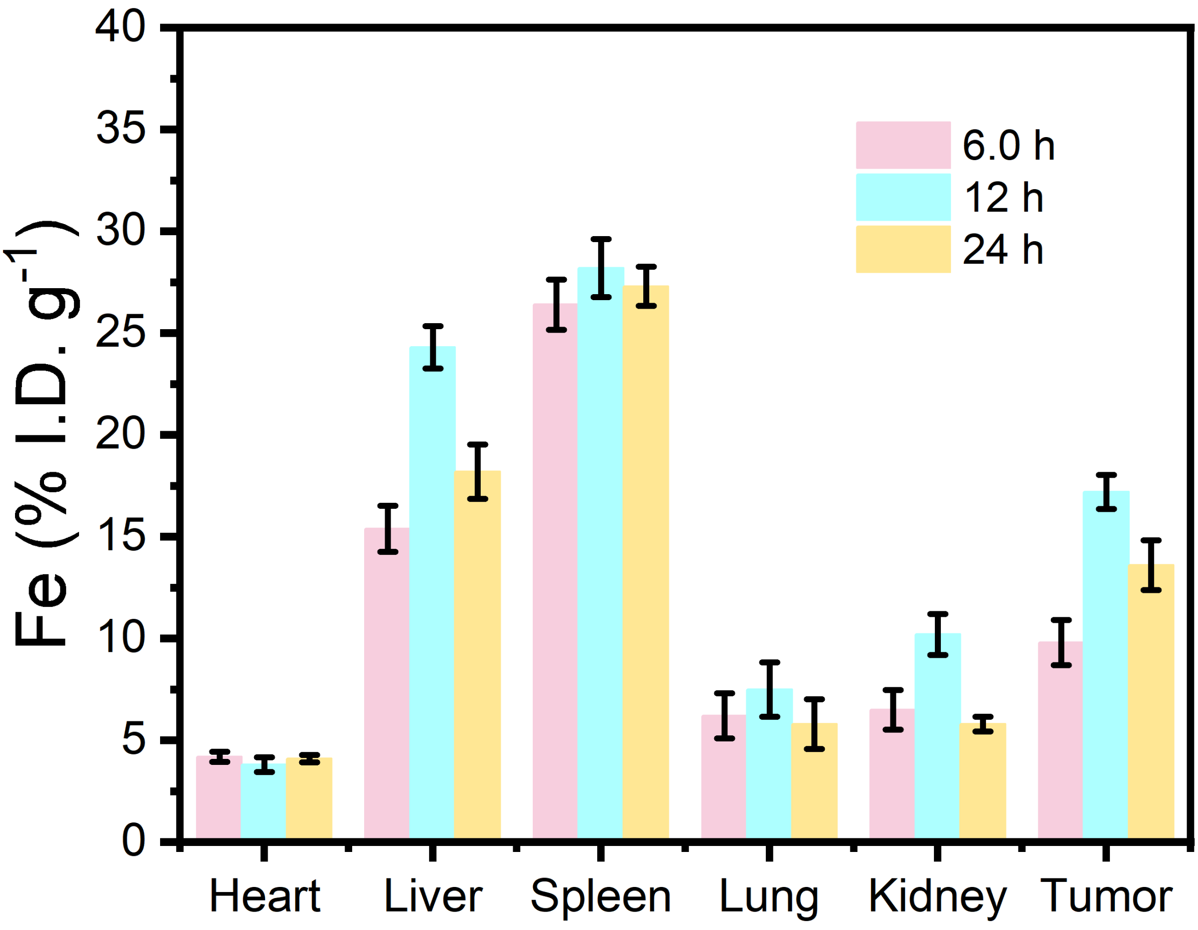

Figure S39. Biodistributions of Fe in major organs and tumors of mice at 6.0-24 h post-injection (*i.v.*) of FeGP4@HMON-SNO (Fe dosage = 5.0 mg/kg). Data are presented as means ± SD (n = 3).

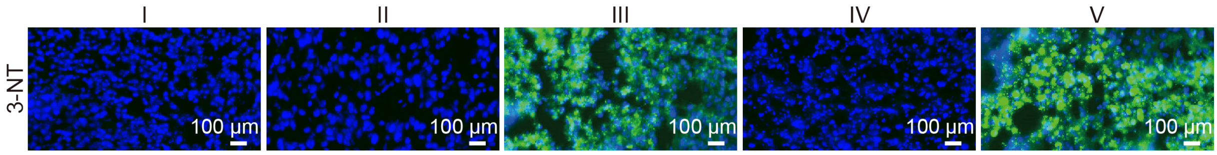

Figure S40. Histological observation of the tumors with staining of 3-NT treated with saline (I), HMON (II), HMON-SNO (III), FeGP4@HMON (IV), or FeGP4@HMON-SNO (V).

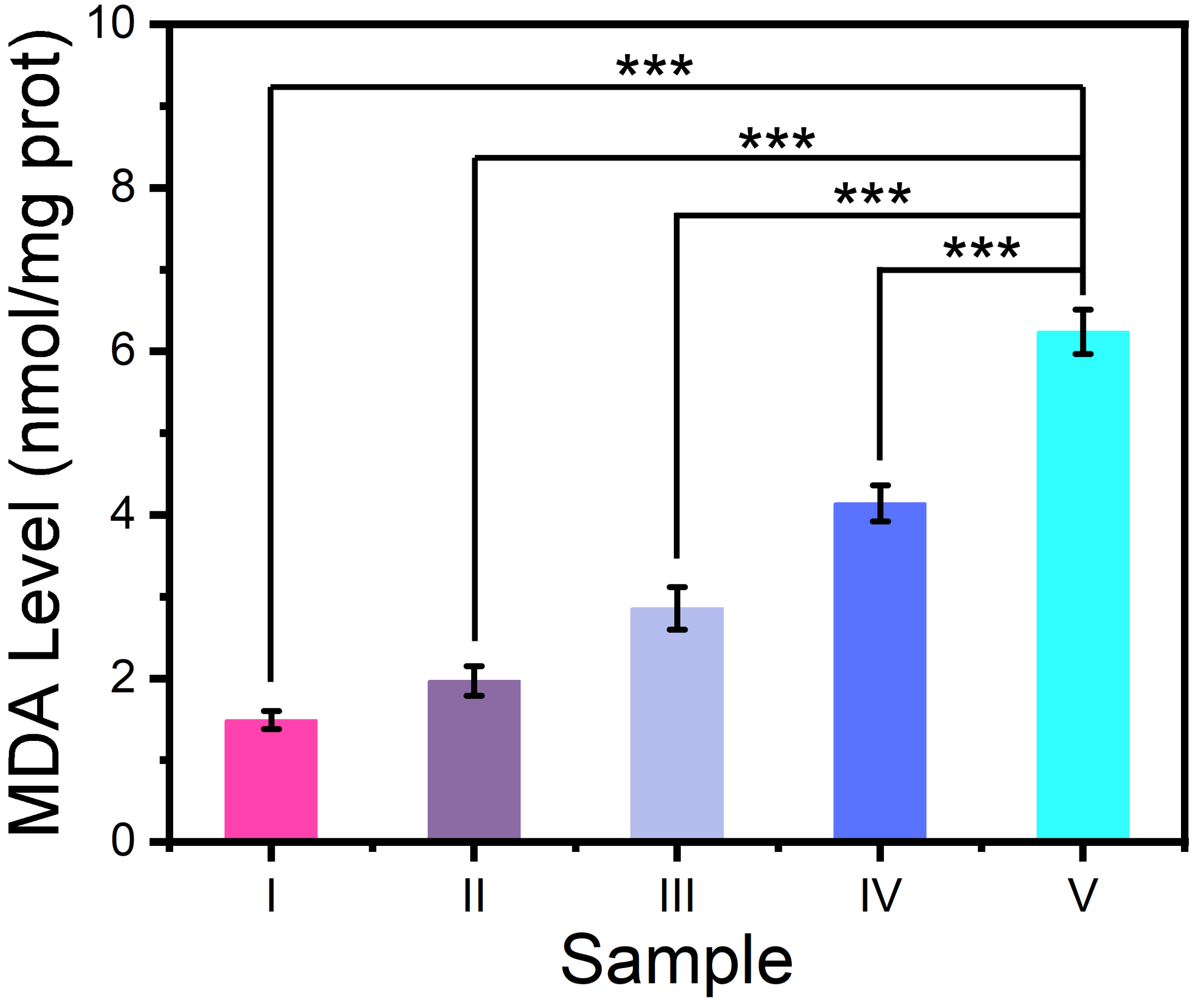

Figure S41. MDA levels in tumor tissues collected from 4T1-bearing mice treated with saline (I), HMON (II), HMON-SNO (III), FeGP4@HMON (IV) and FeGP4@HMON-SNO (V).

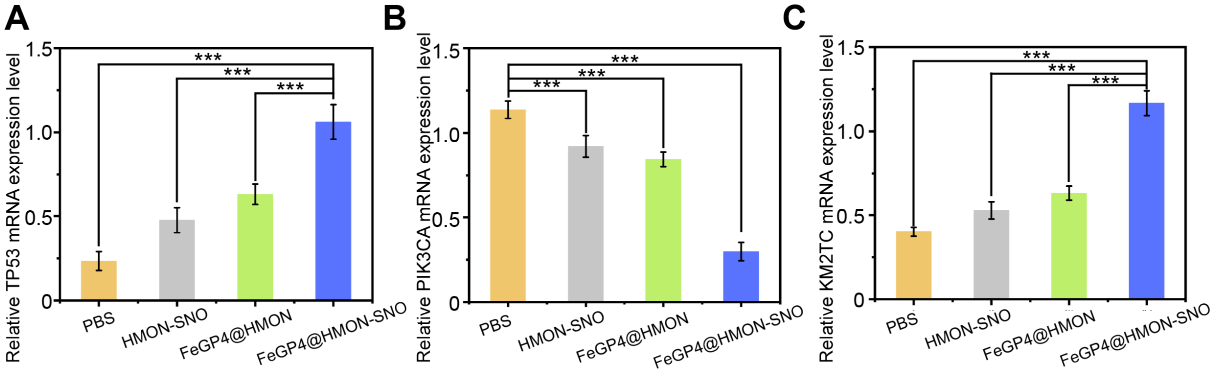

Figure S42. mRNA expression levels of the tumor suppressor genes TP53 and KMT2C, as well as the tumor-promoting gene PIK3CA, in collected tumor tissues following treatment with saline, HMON, HMON-SNO, FeGP4@HMON, or FeGP4@HMON-SNO, as assessed by qPCR.

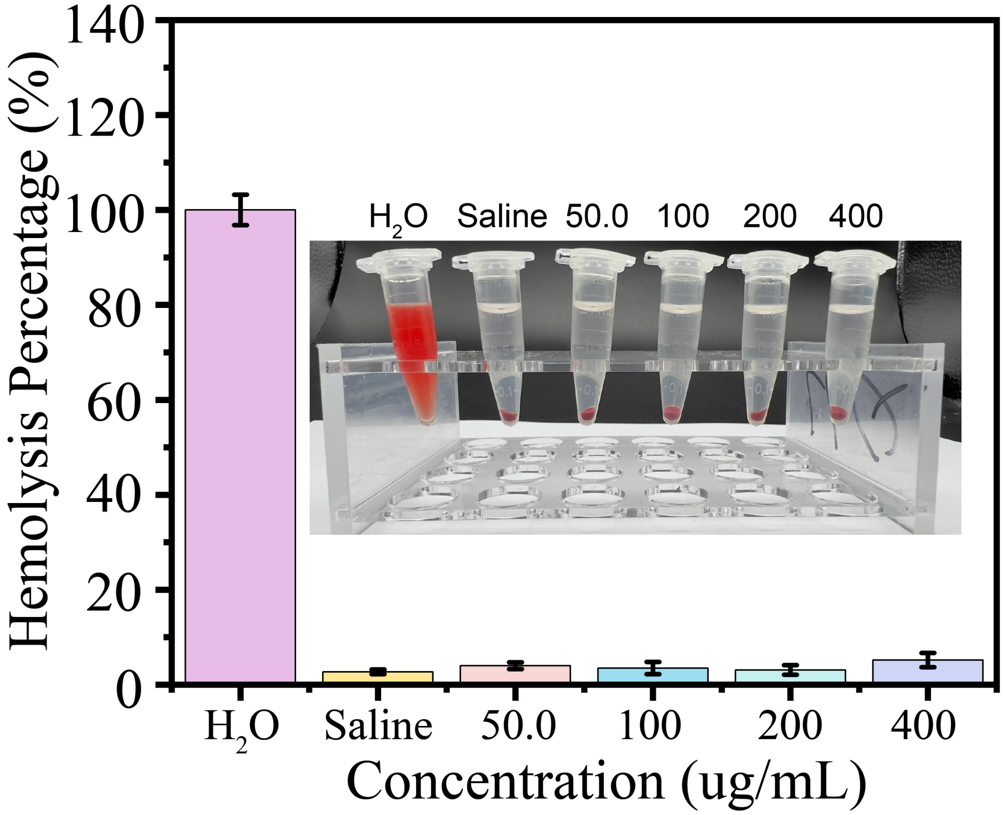

Figure S43. Hemolysis analysis of FeGP4@HMON-SNO at different concentrations. Mean ± SD, $n = 3$.

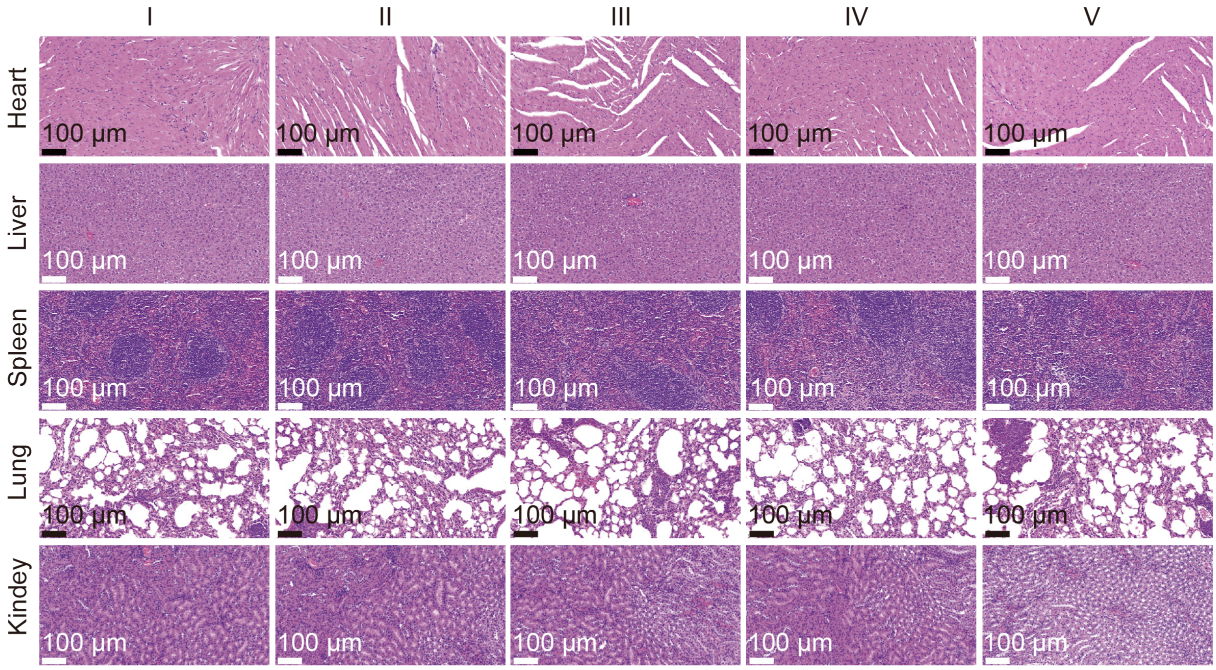

Figure S44. Representative optical microscopic images of the H&E-stained heart, liver, spleen, lung, and kidney from 4T1 tumor-bearing mice after treatments with saline (I), HMON (II), HMON-SNO (III), FeGP4@HMON (IV), or FeGP4@HMON-SNO (V).
